# Ventral body wall closure: Mechanistic insights from mouse models and translation to human pathology

**DOI:** 10.1002/dvdy.735

**Published:** 2024-09-25

**Authors:** Caroline Formstone, Bashar Aldeiri, Mark Davenport, Philippa Francis‐West

**Affiliations:** ^1^ Department of Clinical, Pharmaceutical and Biological Sciences University of Hertfordshire Hatfield UK; ^2^ Department of Paediatric Surgery Chelsea and Westminster Hospital London UK; ^3^ Department of Paediatric Surgery King's College Hospital London UK; ^4^ Centre for Craniofacial and Regenerative Biology, King's College London London UK

**Keywords:** bladder or cloacal exstrophy, ectopia cordis, exomphalos, myofibroblasts, thoracoabdominoschisis, ventral body wall development

## Abstract

The ventral body wall (VBW) that encloses the thoracic and abdominal cavities arises by extensive cell movements and morphogenetic changes during embryonic development. These morphogenetic processes include embryonic folding generating the primary body wall; the initial ventral cover of the embryo, followed by directed mesodermal cell migrations, contributing to the secondary body wall. Clinical anomalies in VBW development affect approximately 1 in 3000 live births. However, the cell interactions and critical cellular behaviors that control VBW development remain little understood. Here, we describe the embryonic origins of the VBW, the cellular and morphogenetic processes, and key genes, that are essential for VBW development. We also provide a clinical overview of VBW anomalies, together with environmental and genetic influences, and discuss the insight gained from over 70 mouse models that exhibit VBW defects, and their relevance, with respect to human pathology. In doing so we propose a phenotypic framework for researchers in the field which takes into account the clinical picture. We also highlight cases where there is a current paucity of mouse models for particular clinical defects and key gaps in knowledge about embryonic VBW development that need to be addressed to further understand mechanisms of human VBW pathologies.

## Key Findings

- Unique synthesis of information, combining developmental biology knowledge with a clinical perspective.
- Embryonic origins of the ventral body wall together with key genes which drive cellular and morphogenetic processes involved in ventral body wall development.
- Defines more than 80 mouse models that exhibit ventral body wall defects and discusses insight gained from these models and their relevance with respect to human pathology.

## 1 INTRODUCTION AND AIMS

The ventral body wall (VBW) consists of the skin, dermis, sternum, ribs intercostal and abdominal musculature that enclose the thoracic and abdominal cavities. The VBW arises during embryonic development in two key steps. First, the primary body wall (PBW), consisting of a thin mesodermal/ectodermal layer, covering most of the ventral embryo, except at the level of the umbilicus, is generated during embryonic folding. Second, the PBW is invaded by the secondary body wall (SBW) comprising of lateral plate and paraxial‐mesoderm‐derived tissues adjacent to the PBW. “Closure” refers to the process whereby the PBW has been infiltrated by the two opposing SBWs which then meet at the ventral midline of the embryo. The initially large umbilicus or “umbilical ring” (UR) has narrowed to contribute to the umbilical cord. At this point, the VBW is “closed.” Anomalies in VBW development include thoracoabdominoschisis (TAS), ectopia cordis, exomphalos, and bladder exstrophy which can accompany pelvic and genital defects (see definition box, Table 1). The most severe of these is TAS, the complete failure to form the VBW, which is incompatible with life. Most VBW defects are sporadic, and the etiology is multifactorial.

**TABLE 1:** Definition box.

Term	Definition
Abdominal bands	Heterogeneous cell population at leading (most ventral) edge of abdominal SBW
Axes of embryonic development	
Cranial‐caudal	Head to tail. Equivalent to anterior–posterior in embryology
Dorso‐ventral	“Back to belly.” Equivalent to posterior–anterior in clinical definitions
Bladder exstrophy	The bladder is not covered by the VBW
Cloacal exstrophy	The cloaca, the progenitor of the bladder and colon, has not separated and is also not covered by the VBW
Cordis ectopia	The heart is external to the thoracic VBW
Exomphalos	Also known as omphalocele. The midgut (and sometimes the liver) are outside the VBW but contained within the umbilicus
Gastroschisis (GS)	Protrusion of the gastrointestinal tract which is not contained within the umbilicus
Leading edge of VBW	The most ventral edge of the SBW, containing the sternal and abdominal bands
Pentalogy of Cantrell	A group of three to five anomalies that can occur together affecting the heart, diaphragm, and VBW
Planar cell polarity (PCP)	A term used to describe the co‐ordinated polarity of cells or co‐ordinated collective cell movements within a plane of tissue, that is, convergent‐extension and orientated cell divisions.
Primary body wall (PBW)	The first tissue cover, consisting of a thin mesodermal and ectoderm layer, over the ventral surface of the embryo
Secondary body wall (SBW)	The invading paraxial and LPM that will form the definitive VBW
Sternal bands	Heterogeneous cell population at leading (most ventral) edge of thoracic SBW
Thoracoabdominoschisis	An anomaly where the thoracic and abdominal organs are not covered by the VBW
Umbilical ring (UR)	The ectoderm at the junction of the amnion and embryo proper

This review outlines VBW development and discusses the insight gained from animal models into clinical defects. The review is in four sections: (1) the embryonic origin and morphogenesis of the VBW; (2) the molecular regulation of VBW closure; (3) a clinical description of the different types of body wall closure anomalies, the genetic, and environmental influences where known; and (4) an overview of some mouse models that give insights into the genetic and cellular mechanisms of the various VBW anomalies.

## 2 ORIGIN AND MORPHOGENESIS OF THE PRIMARY VBW

### 2.1 Definition of the primary VBW

The PBW is an ambiguous term originally introduced to label the VBW in the mouse embryo immediately post‐turning.^1^ This anatomical description refers to a mesodermal and ectodermal layer that provides ventral cover to the intraembryonic coelom in early organogenesis. Nevertheless, VBW closure is a dynamic process, and different cellular arrangements cover the ventral body organs at different stages during embryogenesis. We will use the term “PBW” to refer to the first mesodermal‐ectodermal tissue cover to the intraembryonic coelom in the post‐turning embryo.

### 2.2 The embryogenesis of the primary VBW

**FIGURE 1:**
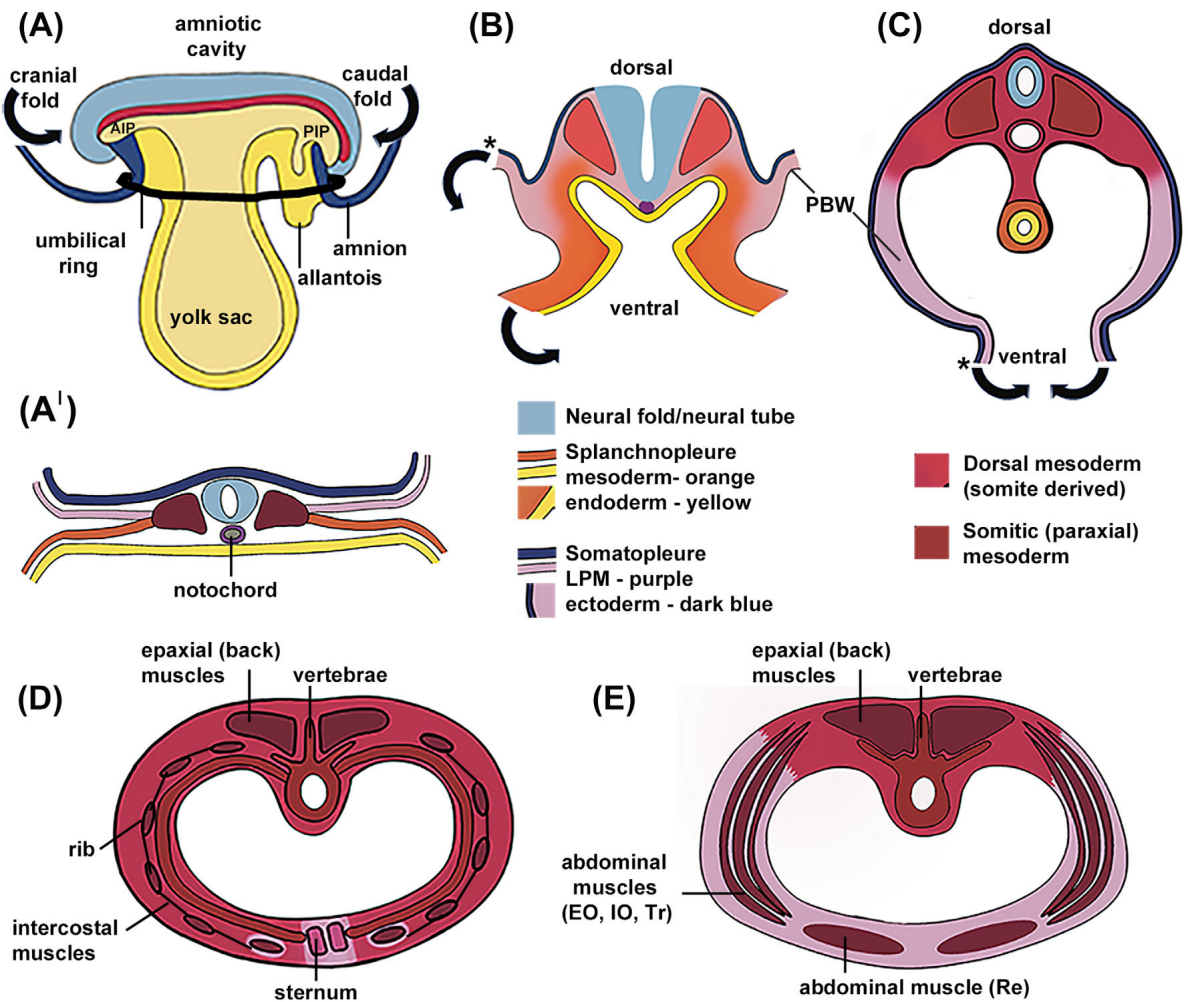
Ventral body wall folding and the contribution of the paraxial and lateral plate mesoderm. (A–C) Progression of embryonic folding to generate the primary body wall from the early gastrula stage (A, A′), to later developmental stages, B, and then C. The stages shown are after embryo turning and model VBW closure in humans, mouse and chicks. In embryonic mice, Gata4, Furin, Hand1/2 control, the step shown in (A), Pitx2 the step shown in (B), and Six5/6 the stage shown in (C). (A) A lateral view while (A′, B, C) are transverse sections. The ectoderm is continuous with the amnion and the endoderm is continuous with the yolk sac. The amniotic sac/cavity initially lies above the embryo (A). The embryo folds laterally and along the cranial‐caudal axes such that the splanchnopleure is ultimately located in the center of the embryo as a blind ended tube. The somatopleure folds around to enclose the VBW. This somatopleure folding also brings the amnion around the embryo such that the amniotic sac now surrounds the entire embryo. (B, C) * demarcates the position of connection to amnion. (D, E) Schematics of the contribution of the paraxial mesoderm and LPM to the thoracic and abdominal ventral body wall, respectively, based on fate mapping studies in the mouse.^3^ The paraxial/LPM contribution varies between species (see Section 3.5) and although not proven, the mouse embryo is anticipated to more closely model human development compared to the chick. The distal intercostal muscles move just ahead of the ribs and become encapsulated by LPM.^3^, ^4^, ^5^ Cranial‐caudal and dorso‐ventral axes are indicated. Block arrows indicate the direction of folding. AIP, anterior intestinal portal; EO, external oblique muscle, IO, internal oblique muscle, PIP, posterior intestinal portal. Tr, transversus abdominis muscle, Re, rectus abdominis muscle. Schematic (D) shows the human anatomy, panniculus carnosus muscle of mouse embryo is not shown. Outline for (B) and (D, E) based on Beddington and Robertson^6^ and Scaal,^7^ respectively.

**FIGURE 2:**
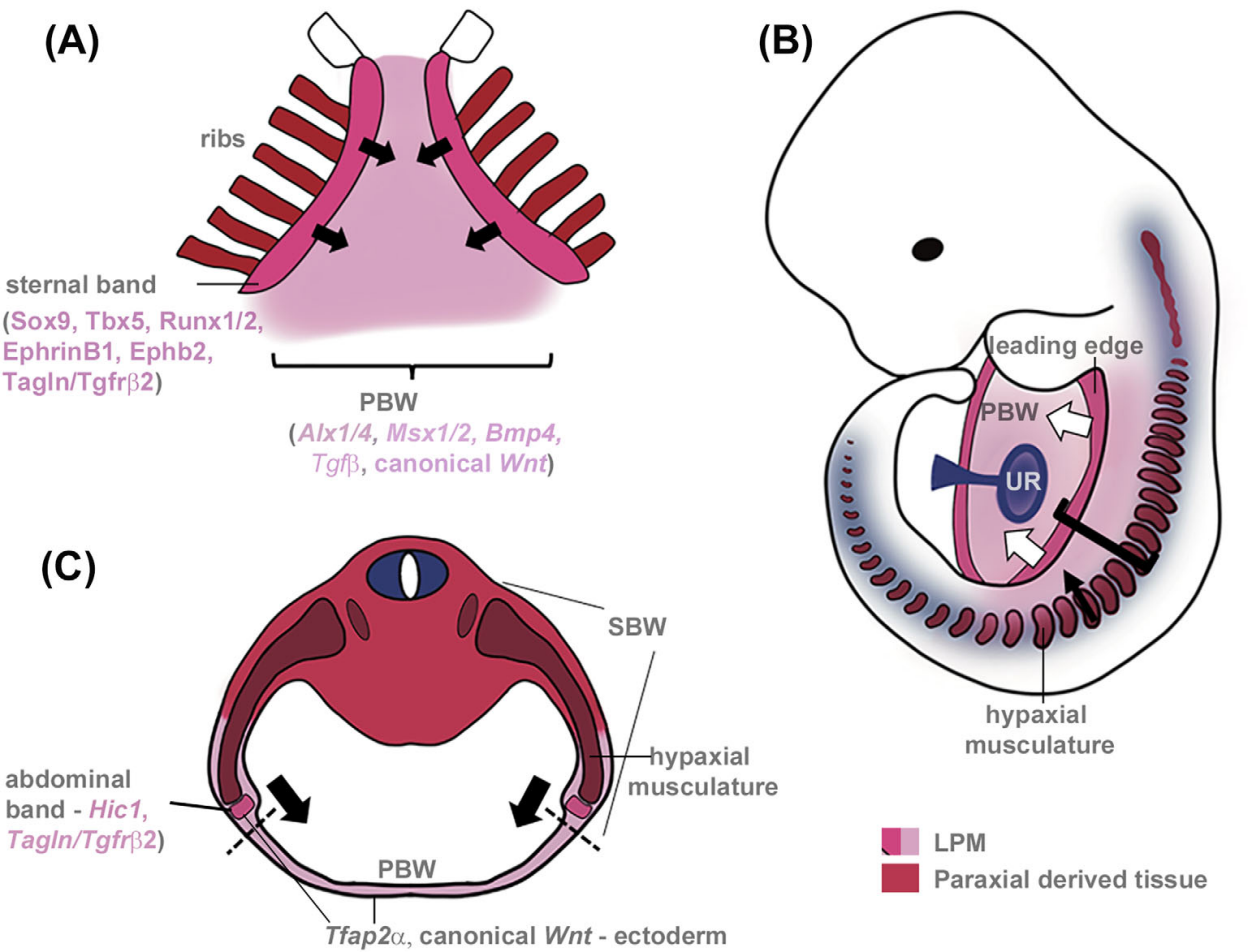
Development of the secondary body wall. (A) Frontal, (B) semi‐lateral, and (C) transverse views of the mouse developing VBW at stages E12.5 (A, B) and E13.5 (C). The leading edge of the SBW (dark pink) followed by the adjacent tissue invades the PBW. The leading edge contains myofibroblasts that are essential for VBW closure and is ahead of the developing ribs/musculature and innervation which arise from the paraxial mesoderm and neuroectoderm, respectively. In the thoracic SBW, the leading edge also contains the mesosternal precursors. Bracket in (B) indicates SBW. The umbilical ring (UR) is the point of transient physiological herniation of the midgut: Here, a mesodermal layer separates the abdominal cavity of the embryo from the umbilical cord. Block arrows indicate the direction of cell movement. Secreted signals from the PBW control development of both the PBW and SBW. Genes expressed within the leading edge are also essential for advancement of the SBW and VBW closure. Dashed line in (C) indicates boundary between PBW and SBW. PBW, primary body wall, SBW secondary body. Outline for (A) and (C) based on Mao et al.^8^ and Nichol et al.,^9^ respectively.

Understanding the embryogenesis of the “primary VBW” in vertebrates requires concurrent understanding of how the discoid shaped embryo folds to take the embryo proper “bean‐shaped” configuration. In rodents, but not in human or chick embryos which are flat, this folding is linked to a simultaneous 180‐degree embryo rotation around the dorso‐ventral axis. Although there is huge variability in the degree to which different vertebrate embryos fold (and turn), the overall process is comparable and results in the creation of body cavities and a PBW (reviewed in Reference 2). The midline is one of the first distinctive regions to form in the developing embryo with the formation of the primitive streak. The dorso‐ventral and cranial‐caudal axes are defined in the trilaminar discoid‐shaped embryo which consists of the three embryonic germ layers, ectoderm, mesoderm, and endoderm (Figure 1A′). Of particular importance for VBW development, the mesoderm starts to form its definitive components, the lateral plate mesoderm (LPM), intermediate mesoderm and paraxial mesoderm (Figure 1A′). Of these mesodermal components, the LPM plays an essential role in the creation of the first mesodermal cover to ventral embryonic structures (Figures 1A′–E and 2A–C). The LPM divides into a dorsally located somatic leaflet underlying the surface ectoderm (these two layers are referred to as somatopleure) and a ventrally located splanchnic leaf that lines the endoderm (these two layers are referred to as splanchnopleure) (Figure 1A′).

Next, the committed germinal layers organize into definitive organs. In preparation for organogenesis, the embryo is required to provide an enclosed and shielded intraembryonic space to allow for the growth and development of the future organs while separated from the extraembryonic space. The intraembryonic coelom, the space between the splanchnic and somatopleure divisions of the LPM that is allocated for future viscera, is at this stage thin and lies in continuity with the extraembryonic coelom laterally. In addition, the right and left, cranial and caudal portions of this space are not yet in continuity. To accommodate the next stage in development the vertebrate embryo folds along the cranial‐caudal and dorso‐ventral axes (Figure 1A–C). Folding around the cranial‐caudal axis occurs at the anterior (cranial) intestinal portal (AIP) and posterior (caudal) intestinal portal (PIP), respectively (Figure 1A). In rodents and chicks, it is thought that the higher proliferation in the left somatopleure and splanchnopleure (compared to the right side) together with the attachment to the extraembryonic membrane is essential for dorso‐ventral folding.^10^, ^11^, ^12^

At the end of the folding process, the endoderm sits within the cavity of the intraembryonic coelom, covered by the splanchnic mesoderm (Figure 1C), and the various elements of the intraembryonic coelom are now adjacent to one another. The amniotic membrane, which is continuous with the somatopleure, now surrounds the embryo on both its dorsal and ventral surfaces. The four folds of the somatopleure form the PBW, the first cover of the intraembryonic coelom, separating it from the amniotic cavity (Figure 1B,C). The only connection between the intraembryonic coelom and the extraembryonic coelom is at the edges of the yolk sac, and this will in turn completely separate when full ventral midline closure ensues. The folding somatopleure edges of the embryo where they connect with the amniotic membranes is called the UR (Figures 1A and 2B). The umbilical ring  (UR) is the site of a thickened domain of ectoderm, which undergoes ectodermal apoptosis, epithelial‐mesenchymal transformation (EMT), and cell deposition.

## 3 ORIGIN AND MORPHOGENESIS OF THE SECONDARY VBW

### 3.1 Definitions and origin of the secondary VBW

The SBW constitutes the skin, ribs, sternum as well as the thoracic, abdominal and pelvic musculature and connective tissues. During development, the SBW is defined as the tissues, including the musculoskeletal components, that will invade the PBW. The SBW has three embryonic origins: the paraxial (somitic) mesoderm and LPM (somatopleure, also called somatic layer) together with the overlying ectoderm. The paraxial mesoderm forms the ribs, hypaxial musculature (the abdominal and intercostal muscles), and contributes to the dermis of the skin (Figure 1D,E).^13^, ^14^, ^15^, ^16^, ^17^, ^18^ The LPM gives rise to the sternum, and also contributes to the dermis of the VBW (Figure 1D)^3^, ^19^, ^20^; reviewed by.^7^ The relative contributions of the LPM and paraxial mesoderm to the VBW dermis, and connective tissues varies along the cranial–caudal axis (see Figure 1D,E and Section 3.5). The LPM also gives rise to the pelvic girdle (ilium, ischium, pubis).^21^ Although the pelvic girdle is not a VBW derivative, it can be affected by VBW anomalies.

### 3.2 Formation and morphogenesis of the VBW skeleton

The ribs develop from the sclerotome portion of the somite (paraxial mesoderm) and can be divided into two domains, the proximal (costal head, neck, and tubercles) and distal rib shaft which arise from different regions of the sclerotome (reviewed by Scaal^7^). Different developmental networks control proximal versus distal rib formation. This is clearly shown by mouse mutants; the *Pax1* mutant, *undulated*, lacks the proximal ribs whereas in *Pax3*, *Splotch*, mutants, the distal ribs fail to form.^22^, ^23^, ^24^ Distal rib extension and differentiation requires signals from the PBW ectoderm and the adjacent developing intercostal musculature (Figure 2A).^18^, ^25^

The sternum develops independently of the ribs and requires signals from the overlying ectoderm.^18^, ^19^ Anatomically and developmentally the sternum is divided into three domains: the presternum (or manubrium), the mesosternum (or body of sternum), and the xiphoid process. In humans and mice the presternum arises from multiple cartilage condensations and may also receive contributions from the neural crest while the mesosternum arises from the “sternal bands” contained within the leading most ventral edge of the SBW (see Figure 2A and Section 4.2.2).^19^, ^20^, ^26^, ^27^, ^28^ In the E12.5–E13.5 mouse embryo, the sternal bands can be visualized as a condensation of densely‐packed cells which express chondrogenic and osteogenic markers.^4^, ^8^ In mice, the two opposing sternal bars meet at the midline by E14.5 after which they undergo cartilage differentiation.

### 3.3 Formation and morphogenesis of the VBW musculature

Studies in chick and mouse indicate VBW musculature arises from the hypaxial myotome in the somite and that the myotome extends into the VBW (Figures 1D,E and 2B,C).^29^, ^30^ This muscle mass becomes histologically visible around day 33 (CS15) of human development.^31^, ^32^ By day 48 (CS19) muscle progenitors have covered 50% of the distance toward the ventral midline and by day 54 (CS 21) have reached the ventral midline to surround the UR. The abdominus rectus muscle and rectus sheath clearly abut the UR by day 56 (CS23).^31^, ^32^

Studies in mouse embryos reveal a similar overall pattern of development but muscle development occurs earlier in the human embryo when compared to the mouse embryo. Myotomal extension starts at E11.5 and by E14.5 myoblasts have reached the ventral midline. Mice develop an additional abdominal muscle layer, the panniculus carnosus (PC), which lags slightly behind the other abdominal muscles, both temporally and spatially^9^ and ultimately sits directly under the skin to enable skin “twitching” (reviewed by Naldaiz‐Gastesi et al.^33^).

### 3.4 Formation and morphogenesis of the skin

In the mouse trunk, the primary establishment of the epidermis initiates around E13.5 at the mid‐flank.^34^, ^35^, ^36^ Just prior to this, the mid‐flank surface ectoderm thickens and consists of flattened squamous‐like cells, which form a ridge.^34^, ^35^, ^37^ The disorganized, thickened, mid‐flank ectoderm then undergoes an “outside‐in” (radial) intercalation generating an organized epidermis, consisting of cuboidal cells, which spread dorsally and ventrally fully enclosing the abdomen by E16.^36^ Explant experiments demonstrated that this radial intercalation is tissue intrinsic.^36^ While the signals responsible for epidermis induction are unknown, the site of the future mid‐flank ectodermal thickening at E13.25 is labeled at E12.5 by *Tbx15* expression in the loose mesenchyme underlying the dermis and the most ventral position of the extending myotome. Notably, *Tbx15* expression also expands both dorsally and ventrally in a pattern resembling the formation of the epidermis.^38^ Munger and Munger have proposed that initiation of ectodermal differentiation coincides with the migration of sternal and abdominal bands, that is, the leading ventral edge of the SBW and subcutaneous innervation^35^ (also see Section 4.2.2). In the mouse VBW, dermal differentiation begins at E11.5 at the same time as myotomal extension and the first switch in ectodermal keratin expression, and is dependent upon Wnt/β‐catenin signaling.^39^, ^40^, ^41^

### 3.5 Differential contribution of the paraxial and LPM to the thoracic and abdominal VBW

The VBW consists of both paraxial mesoderm and LPM‐derived tissues. To define the spatial relationship between these two mesodermal populations and to determine the differential contribution to growth, Nowicki and colleagues (2003) performed fate mapping of somitic (paraxial) mesoderm precursors within the thoracic body wall using orthotopic chick‐quail chimaeras.^15^ The epaxial (back) muscle progenitors and proximal hypaxial (intercostal) musculature, together with proximal rib precursors, were visualized as a collective dense population of labeled quail somitic precursors showing that they, together with their connective tissues, are paraxial‐mesoderm‐derived tissues. This is in contrast to the dilution of the ventral most labeled quail somitic precursors marking the distal developing hypaxial (intercostal) muscles and sternal ribs as they intermingled with unlabeled LPM of chick origin. As labeled quail somitic cells encroach into the LPM, the muscle precursors begin to differentiate within the LPM environment.

In the mouse, the relative contribution of the paraxial mesoderm versus LPM has been determined using a *Prx1* reporter mouse which labels cells of the LPM lineage.^3^ This shows that the paraxial mesoderm and LPM contributions are slightly different to that of the chick. In the mouse thorax, only the distal part of the first rib and the distal intercostal muscles are surrounded by LPM‐derived connective tissues (Figure 1D). This study also showed different relative contributions of the LPM to the thoracic versus the abdominal VBW. In the mouse abdomen, the hypaxial musculature is surrounded by LPM except in the most proximal (dorsal) region^3^ (Figure 1E). Thus, the LPM expands significantly in the abdomen compared to its more limited contribution to the thoracic VBW. As the LPM can influence gene expression of the invading paraxial mesoderm cells,^42^ the LPM would be predicted to play a more significant role in the patterning and morphogenesis of the abdominal musculature compared to the musculature of the thorax. For example, gene mutations that affect LPM would not only influence LPM‐derived tissues, such as the sternum, but can impact the development of adjacent paraxial‐mesoderm‐derived tissues such as the distal ribs (reviewed by Burke et al.^43^).

### 3.6 Closure of the VBW

Closure is defined as when the two opposing ventral SBWs have migrated into the PBW to meet at the midline and the UR has narrowed to contribute to the definitive umbilical cord (Figures 1D,E and 2A–C). Abdominal VBW closure, occurs after the midgut has undergone a transient physiological herniation into the umbilicus (between E13.5 and E15.5 in mice and weeks 5–10 in humans). The first point of contact of the opposing ventral SBW leading edges is in the most cranial region of the thoracic wall at the level of the clavicles (E12.5 in mice). Fate mapping studies of the closing secondary thoracic body wall in in vitro explant culture have shown that the ectoderm and mesenchyme move together.^26^ VBW closure proceeds in a caudal direction and, in mice, the thoracic wall is completely closed by E14.5. Closure then also proceeds cranially from the infra‐umbilical region such that closure is now bidirectional. The final point of closure of the VBW is at the level of the umbilicus, that is, the UR occurring at E16.5 in mice and week 10 in humans (Figure 2B). Filamentous actin present in the cells of the UR may be essential for this final closure: in *Rock1* ^*−/−*^ mutants in which the VBW does not close, the thickened ridge of ectoderm characteristic of the UR is absent and there is decreased actin staining.^9^, ^44^, ^45^

The VBW can close in vivo in the absence of the ribs or a differentiated body wall musculature.^46^, ^47^, ^48^ For example, hypoplasia of the ribs, such as in Campomelic dysplasia does not prevent thoracic body wall closure^23^, ^49^ (OMIM 114290). This indicates that the ribs and body wall musculature per se are not necessary, suggesting that another cell population may be required. One candidate is the myofibroblasts, as discussed in Section 4.2.3.

It is also important to note that while many processes may be identical during closure of the thoracic and abdominal wall (such as the requirement for Tgfβ and Wnt signaling), the relative contribution of the paraxial mesoderm and LPM differs (see Section 3.5 and Figure 1D,E), collective cell movements vary and there are molecular differences. As a consequence, abdominal versus thoracic VBW closure is differentially affected by different gene mutations (see Tables 2 and 3). Closure of the abdominal and thoracic VBW also can occur independently.

**TABLE 2:** Summary of genes/pathways implicated in VBW development.

Disrupted gene/pathway	VBW phenotype	Gene/pathway information and mechanisms
**Transcription/DNA binding factors**		
***Alx4***	Exomphalos, pubic diastasis, BE‐like	Paired type homeodomain, expressed in PBW at E9.5‐E12.5, decreasing later. 100% penetrance exomphalos.^50^, ^51^, ^52^ Mechanism: increase in HH signaling in abdominal VBW and decreased mesenchymal migration caudally into the pelvic region.^50^ Also see Increased Shh signaling in Table and discussion in Sections 3.7 and 6.5.
***Alx1*(*Cart1*)*/Alx4***	Exomphalos, Split sternum	Alx1 and Alx4 functionally overlap, both are expressed in PBW mesenchyme.^52^, ^53^ *Alx1* nulls do not have an exomphalos or a split sternum phenotype.^54^, ^55^ Exomphalos phenotype of *Alx4* null is not modified by additional *Alx1* null alleles but thoracic wall closure affected indicated by a split sternum. No evidence of disrupted hedgehog signaling in VBW.^53^
***AP2α* (*Tfap2α*)**	TAS	Expressed in PBW and SBW ectoderm. PBW ruptures by E13.5 resulting in lack of cover for ventral body, SBW formation fails.^56^, ^57^, ^58^, ^59^ See Sections 4, 4.2.2, and 6.1.
***Ataxin1/Ataxin‐1‐like* (*Atxn1/1 L*)**	Exomphalos	Binds DNA/RNA. May interact with Capicua (cic) in nucleus, see below.^60^
***Barx1***	GS	Model: overexpression of Barx1 in mesenchyme/gut sub‐mucosa leads to 100% penetrance of persistent intestinal herniation. Potential mechanism: failure of appropriate mid‐gut morphogenesis and rotation.^61^
***Capicua* (*cic*)**	Exomphalos	HMG‐box containing transcriptional repressor. May interact with **Atxn1/1 L** in nucleus, see above.^62^
***Gli3***	Split sternum, Exomphalos, pubic diastasis	Hedgehog signaling transcriptional component, inhibits Shh signaling.^63^, ^64^ Mechanism: ectopic/increased HH signaling in VBW from E9.5 to E12.5 and increased apoptosis.^63^ Also see “Increased Shh signaling” in Table and Section 6.5.
***AHDC1* (Gibbin protein)**	Exomphalos	AT‐hook DNA binding protein involved in mesoderm and ectoderm patterning.^65^
***Glucocorticoid receptor***	Exomphalos	Nuclear hormone receptor, essential for metabolism and growth. Model: mesenchymal loss of Glucocorticoid receptor expression leads to 100% penetrance of phenotype. Potential mechanism: mechanical defect as elastin and collagen levels and organization are disrupted in umbilical area.^66^
***Grainyhead‐like 2* (*Grhl2*)**	Open thoracic and abdominal body wall	Expressed in non‐neural ectoderm. Potential mechanism: modulation of epithelial cell shape, junctions and tissue mechanics.^67^, ^68^ Also see Section 6.1.
***Hand1***	GS	Basic helix–loop–helix (bHLH) type transcription factor, expressed in extra‐embryonic mesoderm and entire PBW but later restricts to umbilical ring. Model: conditional deletion in LPM. Proposed mechanism: disrupted development of LPM derivatives.^69^ See Sections 4.1 and 6.3
***Hic1*, *hypermethylated in cancer***	Exomphalos	Pox virus zinc‐finger (POZ) domain containing protein. Expressed in somites at E10.5 and at leading edge of SBW by E11.5.^70^, ^71^ Fate mapping studies reveal limb *Hic1* expressing cells are of somitic origin suggesting same origin for *Hic1*‐expressing mesenchyme within VBW.^72^ Function unknown. Also see^73^ and Sections 4.2.2 and 4.2.3.
***Hoxb2/Hoxb4***	Split sternum, PC	Homeobox. Proposed mechanism: disruption to PBW by E11 with decreased *Alx3/4* expression, a thinner PBW by E11.5 and a failure of SBW invasion.^74^, ^75^, ^76^ Also see Section 6.1 and References 74 and 76
***Msx1/Msx2***	LBWC, Exomphalos	Homeobox type, PBW mesenchymal expression and SBW musculature. Proposed mechanism: defective PBW signaling and stalling of SBW enclosure.^77^ Also see Section 6.1.
***p63***	BE	Ectoderm master regulator expressed in ectoderm, urogenital sinus and tail bud at E11.5.^78^ Strong expression of a dominant negative ∆Np63 isoform in ventral bladder epithelium where it exerts an anti‐apoptotic effect and controls development of adjacent splanchnic mesoderm.^78^ See Section 6.5 for further discussion.
***Pitx2***	LBWC	Homeobox type, participates in left–right asymmetry and tissue pattern formation. Asymmetric expression on left side of somatopleure LPM, also expressed in SBW myotome.^79^ Mechanism: failure of somatopleure folding and PBW formation with altered expression of *Hox* and *Tbx* family members.^80^, ^81^, ^82^, ^83^, ^84^, ^85^ See discussions 4.1, 6.1, and 6.2.
***Runx1/Runx2***	Open thoracic wall/EC	*Runx1* and *‐2* highly expressed at E12.5 and E13.5 in sternal band of SBW.^4^ Proposed mechanism: failure of thoracic SBW to invade thoracic PBW. PBW ruptures. Also see Section 4.2.2.
***Six4/Six5***	Exomphalos	Homeobox type, expressed in PBW ectoderm, LPM and mesothelial precursor cells. **100%** penetrance exomphalos exhibited by double homozygote null. Mechanism: defective somatopleure/PBW morphogenesis with decreased proliferation in ectoderm (both sides) and mesoderm (right hand side only) at E10.5 as well as decreased cell survival overall.^86^ Also see Section 4.1.
***Sox C* subfamily** ***Sox4*, *Sox11*, *and Sox12***	Split sternum, Exomphalos	HMG‐type. *Sox11* is expressed early in somitic mesoderm (E10.5–E11.5).^87^ *Sox11* mutants have exomphalos.^88^ There is redundancy with Sox4 and Sox12: *Sox11* ^*+/−*^ */Sox4* ^*+/−*^ embryos have exomphalos while additional loss of *Sox12*, also results in a split sternum.^89^ Proposed mechanism within VBW: decreased cell survival and proliferation or loss of somitic contribution to VBW.
***Zic3***	LBWC (low penetrance)	Zinc finger type transcription factor deleted in *Bent tail* mouse mutant. Zic3 determines L‐R asymmetry.^90^ Potential turning defect, very low penetrance of VBW defect at E15.5.^91^ Note *Zic3* nulls do not have a VBW defect^90^ indicating Zic3 may be a modifier gene increasing the risk of a VBW defect when together with other gene mutations and /or environmental factors.
**Extracellular signaling ligands and receptors**		
**BMP signaling** ***Alk3* (receptor)** ***Bmp2* and *Bmp4* (ligands)**	Split sternum, Open thoracic wall Exomphalos	Cardinal signaling pathway for embryonic growth and differentiation. Redundancy between Bmp2 and Bmp4 with dosage‐dependent phenotypes ranging from a Split Xiphoid (*Bmp2* or Bmp4 heterozygotes) to exomphalos (*Bmp2/Bmp4* double heterozygotes; hypomorphic *Bmp2* mutant) to an open thoracic and abdominal wall (*Bmp4* deficient embryos).^92^, ^93^, ^94^ Potential mechanism: PBW forms normally but *Alk3* mesoderm specific null suggests BMP signaling role from PBW to drive SBW development.^95^ Reduction in Bmp signaling in VBW and in gastrointestinal tract affecting gut looping in chick also results in VBW defects.^96^ Bmp2/4 may also regulate *Msx1*/*Msx2* expression in PBW. Also see Section 6.6.
**TGFβ signaling** ***Tgfβr2*, *Alk5* (receptors)** ***Tgfβ2/Tgfβ3* (ligands)**	Open thoracic and abdominal wall: Failure of entire SBW development	Cardinal signaling pathway for embryonic growth and differentiation. Failure of thoracic and abdominal wall SBW development. 100% penetrance in *Tgfβ2*/*Tgfβ3* double null embryos,^97^ *Alk5* (*Tgfβr1*) ‐mesenchyme specific deletion^98^ and *Tgfβr2* conditional null in ventral myofibroblasts.^99^, ^100^ *Tgfβ2* null—split sternum.^101^ Potential mechanism: Tgfβ signaling from PBW is essential for function of a population of SBW leading edge myofibroblasts.^99^, ^100^ See Sections 4.2.2, 4.2.3, and 6.1 for further discussion.
**EphrinB and EphB signaling** ***Ephrin B1 and EphB2/EphB3***	Delayed fusion of sternum Exomphalos	Membrane anchored signaling at cell–cell contacts. Role in cell adhesion/cell sorting/migration via regulation of cytoskeletal dynamics. In VBW EphB transmembrane proteins act as ligands to activate reverse signaling by Ephrin B transmembrane proteins.^102^, ^103^ *EphB2*, *EphB3 and EphrinB1* expression observed at the leading edge of SBW.^102^, ^104^ Knockout studies; phenotype in *EphrinB1* ^*+/−*^ females but not males.^104^ *EphB2/EphB3* double mutants.^17^, ^102^ Cre inactivation studies indicate likely origin of essential EphrinB1 cells is somitic mesoderm.^103^, ^104^ See Sections 4.2.2 and 4.2.3 for critical roles of somitic‐derived cells at VBW leading edge.
**FGF signaling** ***Fgf8/9/17/18* (ligands)** ***Fgfr1 and Fgfr2* (*receptors*)**	Exomphalos	Cardinal cell signaling pathway for embryonic growth and tissue differentiation. *Fgfr1* and *Fgfr2* expression: somites, abdominal PBW and SBW.^105^ Proposed mechanism for *Fgfr1*/*Fgfr2*: Defective signal from PBW resulting in thickening of dermis and underlying connective tissues within SBW as a consequence of stalled ventral‐ward movement of SBW.^105^ Phenotype resembles *Hoxb4* mutants. See also MAPK signaling Fgf8,17,18 ligand inactivation show key functions in presomitic and somitic mesoderm necessary for VBW closure. It is proposed that the smaller somites contribute fewer cells (muscle, migratory cells) to the SBW.^106^
***Furin***	Open ventral body	Serine protease involved in processing of secreted proteins and membrane proteins including **Tgfβ/Bmp** family members. Expressed in AIP and PIP at E8.5. Mechanism: Failure of somatopleure folding to form PBW.^107^ Does not recapitulate recognized human pathology. See Section 4.1.
***IGFII* over‐expression**	Split sternum, Exomphalos	Ligand required for embryonic growth. 91% penetrance of exomphalos. Also see Section 6.1. Proposed mechanism: organ over‐growth e.g. enlarged liver and heart (organomegaly) disrupts body wall morphogenesis. However, also disrupted eyelid and palate fusion suggesting possible underlying defect in tissue movement/tissue fusion processes.^108^
***Pdgf*** **Pdgfrα (receptor)**	Short split sternum, Exomphalos	Tyrosine kinase receptor. PDGFRα expressed in PBW and somitic derivatives.^109^, ^110^, ^111^ Proposed mechanism: stalled SBW differentiation and ventral‐ward movement^111^, ^112^, ^113^ linked to increased cell death in somitic mesoderm and E9.5–E11.5 VBW.^112^, ^114^ Activates PI3K intracellular pathway to control VBW development.^115^
**Increased SHH signaling**	Exomphalos, pubic diastasis	Cardinal cell signaling pathway for embryonic growth and tissue differentiation. Proposed mechanism: graded increase in SHH signaling as reported for compound allelic series of *Gli3* coupled to ***Alx4*** mutant alleles—observe increased penetrance of phenotype.^63^ Recovery of phenotype achieved through additional removal of one allele of *Shh*. Ectopic induction of SHH signaling also reported in R*26‐SmoM2*:*CAGG cre‐ER* mice (constitutively active Smoothened) which led to disorganized hypoplastic abdominal muscles and excessive cell death in VBW at E12.5 associated with an enlarged umbilical ring.^63^ Also see SuFu and Section 6.5.
**Wnt Signaling** **Wls** **Lrp5/6 receptors** **Porcupine**	Ectopia cordis, Split sternum, Exomphalos, Bladder exstrophy‐like	Cardinal cell signaling pathway for embryonic growth and tissue differentiation. Mouse models; Wls, essential for Wnt ligand secretion; Lrp5/Lrp6, core obligatory membrane proteins for canonical Wnt signaling; Porcupine (Porcn), acyltransferase enzyme, essential for Wnt ligand palmitoylation and function. A variety of Wnts (canonical and non‐canonical) are expressed within the mesenchyme and ectoderm of PBW and SBW.^49^, ^116^ Canonical Wnt signaling is active in the PBW and SBW midline.^39^, ^49^ Ectodermal and mesenchymal Wnt ligands control PBW morphogenesis and SBW mesenchymal cell survival, proliferation, migration and dermal/myogenic differentiation.^49^, ^116^, ^117^, ^118^ Also see Sections 4.2.4, 6.1, and 6.2 together with Wnt5a and β‐catenin in Table.
**Tissue polarity and non‐canonical Wnt**		
***Fat4*, *Dchs1***	Wider, thinner and shorter sternum	Protocadherins Fat4 and Dchs1 are a receptor‐ligand pair expressed in sternal bands. Mechanism: control of collective cell intercalation behaviors.^8^ See Section 3.7.
***Celsr1***	Exomphalos	Adhesion‐GPCR, seven‐pass transmembrane protein with key role in core Frizzled‐PCP. Expressed in ectoderm and epidermis throughout development.^119^ Disruption to epidermal morphogenesis.^36^ Genetic interaction with ***Vangl2*** (*loop‐tail*) and ***Scribble*.** ^120^ See Sections 4.2.4 and 6.1.
***Scribble***	LBWC, Exomphalos	Apico‐basal polarity determinant associated with Frizzled‐PCP signaling in mammals. Expressed in somitic mesoderm, PBW mesenchyme, ectoderm/epidermis of VBW at E13.5.^121^ Range of phenotypes from exomphalos to shortened and skewed body axis with open abdominal VBW.^120^, ^121^, ^122^, ^123^, ^124^ Genetic interaction with ***Vangl2*** (*loop‐tail*) and ***Celsr1*.** ^120^, ^123^ See Sections 4.2.4 and 6.1.
***Ptk7***	LBWC	Unusual receptor protein tyrosine kinase associated with Frizzled‐PCP signaling in mammals but also regulates canonical Wnt signaling. Shortened and skewed body axis and truncated hindlimbs associated with open abdominal VBW.^125^, ^126^ See Sections 4.2.4 and 6.1.
***Vangl2***	LBWC	Key component of Frizzled‐PCP, four‐pass transmembrane protein. Shortened and skewed body axis and truncated limbs associated with open abdominal VBW. Genetic interaction with ***Ryk*,** ^127^ ***Celsrl*** and ***Scribble*.** ^120^, ^123^ See Sections 4.2.4 and 6.1.
***Ryk***	LBWC	Receptor tyrosine kinase type but catalytically inactive Wnt receptor, part of Wnt‐PCP pathway. *Ryk* null‐no phenotype but loss of *Ryk* enhances severity of *Vangl2* heterozygous or *Vangl2* null phenotype, that is, the shortening of body axis, limb truncation and exomphalos.^127^ See Sections 4.2.4 and 6.1.
***Ror1/Ror2***	Delayed SBW closure	Receptor tyrosine kinases and Wnt receptors, part of Wnt‐PCP pathway. Truncated cranial‐caudal axis and limb anomalies in *Ror1*/*Ror2* double mutants, resembling *Wnt5a* null mutants.^128^, ^129^ Evidence of delayed VBW closure. Mechanism unknown but **Wnt5a**‐**Ror** signaling drives coordinated tissue movements. See Sections 4.2.4 and 6.1.
***Wnt5a***	Delay in SBW formation Split sternum	Proposed non‐canonical Wnt. Expressed at leading edge of SBW at E11.5–E12.5.^116^, ^130^ Truncated cranial‐caudal axis, shortened limbs, split sternum.^127^, ^130^ Evidence of altered migration and delayed SBW morphogenesis.^49^, ^128^ See Sections 4.2.4 and 6.1.
**Other membrane proteins**		
***Podocalyxin*** (PODXL) mPCLP‐1	Exomphalos, delayed closure of PUH	Heavily sialylated/sulfated membrane protein, downstream target of **Pitx2.** ^82^ 93% penetrance exomphalos up to E17 reducing to 30% for newborn pups. *Podxl* ^+/−^ embryos also show delayed closure of physiological hernia but this is resolved before birth. Proposed mechanism: authors speculate Podxl function in mesothelial cells of the peritoneal lining is antiadhesive facilitating midgut retraction into abdominal cavity.^131^
***Tmem67*** (Meckelin)	Exomphalos	Frizzled‐like protein, integral part of the primary cilium. Proposed mechanism: plays a role in **Wnt5a/Ror2** signaling.^132^
**Extracellular matrix remodeling**		
***Aortic carboxypeptidase‐like protein* (*ACLP*)**	GS	Secreted protein that interacts with ECM, expressed in dermis, increases tensile strength of collagen fibers. 100% penetrance of abdominal wall defect reported by References 133, 134. Potential mechanism: mechanical defect of dermis. Defect at midline—does not model asymmetric GS phenotype in humans. See Section 6.3.
***Bmp1*** (mammalian Tolloid‐like)	GS	Metalloproteinase. Expressed in LPM of somatopleure at E11.5. Potential mechanism: disruption to **TGFβ** signaling and ECM/collagen formation in dermis/connective tissues of PBW. Amnion “loop” that folds around herniated midgut is not present‐ therefore defect arises early, is not asymmetrical and does not model GS in humans.^135^ See Section 6.3.
**Intracellular proteins**		
***β‐catenin* (*core canonical Wnt component*)**	Delayed closure of PUH	***β‐catenin*:** Signaling hub for canonical Wnt signaling as well as cadherin‐based cell adhesion. Canonical Wnt signaling is active in PBW and SBW.^39^, ^49^ Mechanism: required for proliferation, differentiation, and survival of VBW mesenchyme.^39^, ^40^ Zhu et al.^136^ also suggest that *β‐catenin* regulates contribution of LPM to limbs versus abdomen which impacts rate of SBW closure.
***Calreticulin***	Exomphalos	ER Ca2+ binding multifunctional protein.^137^ Also can impact on growth factor signaling such as Wnt and Bmps.^138^, ^139^ Increased expression of pro‐apoptotic proteins p53 and Bax within umbilical membrane at E14.5.^140^ Proposed mechanism: Increased cell death and altered cell migration
***Dermatan Sulphate Epimerase 1* (*Dse1*)**	Exomphalos	Enzyme that converts Glucaronic Acid to Iduronic Acid (IdoA) in Dermatan Sulphate (DS). DS is mainly found in the extracellular matrix as a component of proteoglycans. IdoA containing ECM induces cell migration and proliferation.^141^, ^142^ Proposed mechanism: disruption to dermal collagen maturation^143^, ^144^ and/or epidermal morphogenesis.^143^
***Filamin A* (*Flna*)**	Split sternum, Exomphalos	Actin binding and scaffolding protein, X‐linked and widely expressed. Phenotype present in male *Flna* hemizygous mutant; sternal defect also seen in *Flna* mutant females.^145^, ^146^ Underdevelopment of dermis and muscle, decreased proliferation of VBW leading to a thinner VBW.^145^
***Filamin A/Formin2* (*fmn2*)**	Complete failure of SBW development	Formin‐2 is an actin nucleating protein which can function with Flna. *Filamin* null phenotype more severe in simultaneous absence of *Fmn2* resulting in Thoracoabdominoschisis phenotype.^145^ Decreased proliferation and malformations in skeleton and musculature. Proposed mechanism: Defect in midline tissue fusion and defective growth and differentiation of the SBW. See Section 6.6.
***Folate binding protein‐1***	GS	Folate receptor. Proposed mechanism: Defect in folate metabolism. High dietary folate supplementation in mutant reduces penetrance of VBW defect.^147^ See Section 6.3.
***Hrs***	Open ventral body	Vesicular transport protein, ubiquitously expressed. Mechanism: failure of somatopleure folding. Embryo remains outside yolk sac. Increased cell death primarily in definitive endoderm at E8.5, the time of embryonic folding. Embryonic lethal by E11.^148^
***Trip11* (GMAP‐210)**	Exomphalos	Golgi microtubule associated protein −210. Unknown role in VBW.^149^, ^150^
***Male‐abnormal 21 like2*** ***Mab21l2***	Complete failure of SBW development	Cell fate determinant. *Mab21l2* is expressed in PBW at E9.5–E11.5.^151^ Interacts with **Tgfβ/Bmp** signaling.^152^ Reduced cell number in PBW from E10.5, thinner PBW linked to decreased proliferation at E11.5. Embryonic lethal by E14.5.^151^
***Marcks***	Exomphalos	Calmodulin and actin binding protein.^153^
***Mek1/Mek2***	Exomphalos	MAPK signaling pathway. Model: *Mek1* (*Map2k1*) mesenchymal inactivation in a *Mek2* (*Map2k2*) null background;100% penetrance, PBW forms but SBW does not develop appropriately.^154^
***Mekk4***	GS	MAPK signaling pathway. Mekk4 (Mapk3) signals to p38 and JNK to regulate cell signaling and actin dynamics. Kinase inactive Mekk4 with dominant‐negative activity results in GS.^155^ GS not observed in *Mekk4* null embryos.^156^
***p57kip2* (*CDKN1C*)**	Split Sternum Exomphalos, Umbilical hernia	Negative regulator of cell cycle. Delayed thoracic SBW closure, abdominal muscle migration, and skin differentiation.^157^ See Section 6.1.
***Presenilin‐1***	Umbilical hernia	Multi‐pass ER/Golgi transmembrane protein. *Presenilin‐1* mouse mutants reported to exhibit intestinal herniation.^158^
***SuFu***	Split Sternum, Exomphalos	Negative regulator of **HH** signaling. Hypomorphic allele, 96% penetrant Exomphalos at E18.5. Proposed mechanism: Disruption in HH signaling.^159^
**Cytoskeletal components**		
***Caldesmon*** ***Smooth muscle caldesmon isoform* (*h‐Cad*)**	Split Xiphoid of sternum, Exomphalos	Actomyosin and calmodulin binding protein, potential regulator of acto‐myosin function. 97% penetrance of both phenotypes. Proposed mechanism: mechanical disruption due to changes in actomyosin contractility of smooth muscle cells within VBW or gut mesentery. Abdominal muscle layers present but hypoplastic.^160^, ^161^
***Non‐muscle myosin heavy chain II* (NMHCII)** ***Myh10* (*non‐muscle myosin heavy chain B*)**	PC spectrum	Heavy chain sub‐unit of non‐muscle myosin II (NMHCII) protein. Roles in cell‐adhesion, cell divisions, migration and actin cytoskeleton. Myh9 (heavy chain subunit A) and Myh10 expressed at leading edge SBW at E14.5. Model: Knock‐in of motor impaired Myh10 is a dominant‐negative gain‐of‐function interfering with both Myh9 and Myh10 function. PoC spectrum: Exomphalos (100%), ectopia cordis (50%) or split sternum, diaphragmatic hernia and cardiac defects.^162^, ^163^ *Myh10* null mice do not have VBW phenotype.^164^ See Sections 4.2.5 and 6.1.
***ROCK1/ROCK2***	Exomphalos	Serine/threonine protein kinases which regulate NMII contraction and the formation of supracellular actin cables. Functions downstream of planar cell polarity pathways. *ROCK1* and *ROCK2* expressed in VBW.^165^ Proposed mechanism: Disruption of ROCK‐dependent contraction of supracellular actin surrounding the umbilical ring. ROCK1 can compensate for ROCK2 and *vice versa*.^44^, ^165^, ^166^ Also see Section 4.2.5.
***Specc1l***	Exomphalos	Associates with actin and microtubules. Binds NMIIB. Phenotype observed in gain of function mouse models which express mutated forms of Specc1L (in frame deletions) but not in mouse models which totally lack Specc1L expression (null or out of frame deletions). Altered actin‐cytoskeletal distribution. Actin cables not formed appropriately in ectoderm at E13.5.^167^
***Shroom***	GS	F‐Actin binding protein. *Shroom* is expressed in the somitic mesoderm and PBW at E10.5.^168^ See Section 6.3.
***Nuak1*** (***Omphk1***)	Exomphalos, complete failure of SBW development	Serine/threonine protein kinase, which modulates actinocytoskeleton and inhibits Tgfβ signaling.^169^ *Nuak1* expression at E9.5 in PBW, also expressed later in epidermis, sternal and abdominal bands.^170^, ^171^
**Inhibitory Neurotransmitter system**		
***Gad1*/*Gad2***	Exomphalos	Glutamate decarboxylase (GAD), GABA synthesizing enzyme. Two isoforms: GAD67 is encoded by *Gad1* producing 90% GABA during embryonic development, and GAD65 encoded by *Gad2*. 100% penetrance *Gad1/Gad2* double knockout. Proposed mechanism: increased pressure within thoracic and abdominal cavity as a consequence of hunched posture of mice and/or increased abdominal muscle contractions but a review of GABA function in the gating of chloride channels suggests the GABA pathway may also play a role in tissue growth and tissue movement.^172^, ^173^, ^174^, ^175^, ^176^
***VGAT***	Exomphalos	Vesicular GABA transporter (VGAT) transports GABA and Glycine into synaptic vesicles. 100% penetrant.^172^, ^174^, ^177^
***KCC2***	Exomphalos	Neuronal‐specific Potassium‐chloride cotransporter, establishes chloride ion gradients necessary for GABA/glycine neurofunction.^178^

*Note*: Classification of VBW phenotypes: assessment of published reports on the mouse mutants linked to an interpretation of human VBW pathology. Full details of mouse mutants and phenotypes can be found in supplementary table. VBW is ventral body wall, LPM is lateral plate mesoderm, SBW is secondary body wall, PBW is primary body wall. Mutant phenotypes have been interpreted based on human VBW pathologies, definitions are outlined below. In the majority of mouse mutants, the phenotypes are not fully penetrant. Where a phenotype is fully penetrant, this is stated in the table. **TAS**; Thoracoabdominoschisis, defined here as fully open VBW with internal organs floating in amnion due to lack of a membrane cover. **PoC**; Pentralogy‐of‐Cantrell like, defined here as including fully open VBW with a membrane cover (ectopia cordis and omphalocele) together with anterior diaphragmatic hernia, anomaly of pericardium and structural cardiac defects. **LBWC**; limb–body‐wall complex, defined here as open ventral body wall (with or without amnion cover) with truncated limbs. **Split sternum**: defined as failure of sternal bands to close and/or fuse. **Split Xiphoid of sternum**; bifurcation of the xiphoid process (the caudal part of the sternum). **GS**; gastroschisis, defined here as intestine floating in amnion or sitting in exo‐coelomic space. **Exomphalos;** defined here as liver/intestinal herniation through the umbilical ring maintained after E16.5, with amnion covering. **BE** and **BE‐like**: bladder exstrophy, BE; defined here as an open ventral bladder muscle wall and VBW into the amnion, BE‐like; where the bladder muscular wall is closed but there is a deficient caudal ventral mesenchyme and body wall cover overlying the bladder. **Pubic diastasis**; separation of pubic bones of the pelvis. **PUH**: Physiological umbilical hernia. Also see Mouse Genome Informatics (MGI) website (https://www.informatics.jax.org/phenotypes.shtml) and International Mouse Phenotyping Consortium (IMPC) (https://www.mousephenotype.org/). Mutants of interest identified from website searches not included in Table 2 include the long noncoding RNA, *Fendrr*,^179^ NFkB regulator, *IKK*,^180^ serine protease, *Pcsk5*,^181^ two regulators of the HH pathway in cilia, *Cp110* and *Ift25*,^182^, ^183^ the Filamin A interacting protein, *Luzp1* ^184^ and the aminomethyltranferase, *Amt*, which functions in mitochondrial folate metabolism.^185^ Details of these mutants can be found in the supplementary table.

**TABLE 3:** Chromosomal abnormalities, gene associations, and human syndromes with VBW defects.

Chromosomal region	Human syndrome	Clinical presentation and gene association	Mouse mutant models	Reference
1p31.3 duplication		Exomphalos ***ROR1*, *FOXD3*, *ALG6*, *ITGB3*, *DLEU2L***	*Ror1* ^*−/−*^ */Ror2* ^*−/−*^	128, 129, 185
3q26.31‐q29 duplication	3q duplication syndrome OMIM:611936	Exomphalos ***CLDN1*** and ***CLDN16*** (CLAUDIN‐1/CLAUDIN‐16), ***EPHB3*** Often associated with second chromosomal imbalance, for example, 9q34.3^186^	*EphB2/3* double knockout	17, 104, 186, 187, 188
11p15.5 deletion	Beckwith‐Wiedemann Syndrome (BWS) OMIM: 130650	Exomphalos, prune belly ***CDKN1C* (*p57KIP2*)** has preferential maternal expression. *CDKN1C* germline mutation is associated with increased incidence of exomphalos in BWS patients. Epigenetic silencing of *CDKN1C* also implicated. ***IGF2***: variable loss of imprinting or hypermethylation	*IGF2* over‐expression *p57kip2* ^*−/−*^ Caspary et al, 1999^189^ reported a mouse model with *p57kip2* mutation and loss of *IGF* imprinting	108, 157, 189, 190, 191, 192, 193
Trisomy chromosome 13	Patau Syndrome OMIM: 264480	Exomphalos, bladder exstrophy		194, 195, 196, 197
17p13.3 deletion	Miller–Diecker Syndrome OMIM: 247200	Exomphalos **HIC1** located within the 350 kb critical region for Miller–Diecker Syndrome	*Hic1* ^*−/−*^	70, 73, 198
Trisomy chromosome 18	Edwards Syndrome OMIM: 601161	Exomphalos		196, 197, 199
18q21.3		Exomphalos, ano‐rectal malformation ***PIGN*** within a 20.43 Mb duplication. *PIGN* mutations are also associated with Fryn's Syndrome characterized by congenital diaphragmatic hernia (CDH).^200^		200, 201
19p13.3	Cardio‐facio‐cutaneous Syndrome 4 OMIM: 615280	Exomphalos ***MAP2K2*** within a 1.27 Mb deletion. Belongs to group of RASopathies, germline mutations in Ras/MAPK signaling pathway. Clinically related Noonan Syndrome (OMIM: 163950), mutation in *PTPN11* also reported in patient with exomphalos.^202^	*Mesenchyme‐cre Mek1* ^−/−^/*Mek2* ^−/−^	154, 202, 203
Trisomy chromosome 21		Exomphalos Reduced incidence compared to Trisomy 13 and 18, some studies suggest no association of exomphalos with Down's syndrome.^204^		204, 205
45, X	Turner Syndrome OMIM: 309590	Exomphalos 5.1% of patients with 45,X karyotype (*n* = 680).		206, 207
47XXY	KlineFelter Syndrome Focal Dermal Hypoplasia: Goltz‐Gorlin Syndrome OMIM: 305600	Exomphalos, covered bladder exstrophy ***PORCN***.	Porcn^‐/‐^	118, 205, 208, 209

Gene	Human syndrome	Clinical presentation and gene mutations	Mouse mutant	Reference
***ALX4***	Bladder exstrophy‐epispadias complex (BEEC)	Bladder exstrophy 41 distinct *ALX4* heterozygous missense variants from 7500 patients analyzed.	*Alx4* ^***LstJ/LstJ***^	51, 210
***FILAMIN A* (*FLNA*)**	Otopalatodigital (OPD) spectrum disorder, OMIM:311300 OPD2, OMIM: 304120 Melnick‐Needles Syndrome (MNS) OMIM: 309350	Exomphalos and prune belly *FLNA* mutations reported for all *OPD1* cases and 70% *OPD2*. *OPD2* associated with gain‐of‐function *FLNA* mutations. X‐linked, with males exhibiting the most severe exomphalos phenotypes. Four males with mutations in *FLNA* presented with Prune Belly	*Dilp2* ENU mutation, males exhibit exomphalos.	146, 211, 212, 213, 214, 215
***FREM1***	Manitoba‐oculo‐tricho‐anal (MOTA) syndrome OMIM: 248450	Exomphalos Syndrome first described in patients of Aboriginal origin in Manitoba. Identical deletions of *FREM1* were identified in families from two patients.	*Frem1* ^bat/bat^ does not exhibit exomphalos.	216, 217
***GAD1***		Wider and shorter thorax, exomphalos, rectal diastasis Exomphalos: Two siblings with bi‐allelic loss‐of‐function mutations in the *GAD1* gene, one sibling also presented with a wider and shorter thorax. Rectal diastasis: One patient with a homozygous mutation in *GAD1*, from a cohort of six families. Study of Neuray et al.,^218^ however, found no ventral body wall defects in six individuals with bi‐allelic *GAD1* mutations.	*Gad1* ^−/−^ *Gad1* ^−/−^ */Gad2* ^−/−^ *VGAT* ^−/−^	172, 218, 219
***ISL1***	Bladder exstrophy‐epispadias complex (BEEC)	Bladder exstrophy		220, 221
***LRP2* (*LDLR2*)**	Donnai Barrow Syndrome OMIM: 222448	Exomphalos 50–70% of DBS patients exhibit exomphalos Two studies associate mutations in *LRP2* with exomphalos		222, 223
***PORCN***	Focal Dermal Hypoplasia OMIM: 305600 aka Goltz‐Gorlin Syndrome	Pentralogy of Cantrell (PoC), ectopia cordis, abdominal wall defect 71 disease‐associated mutations in *PORCN*. 90% patients are female thus X‐linked transmission.^224^ PoC reported in three affected individuals. Ectopia cordis reported in two affected individuals. Abdominal wall defect reported in two affected individuals.	*Porcn* ^‐/‐^	118, 224, 225, 226, 227
***PITX2***	Axenfeld‐Rieger Syndrome OMIM: 601499	Exomphalos >40% of ARS patients found with mutations in *PITX2*, exomphalos presents in 4.3% of RS patients.	*Pitx2* deficient mice	228, 229
***SPECC1L***	Opitz G/BBB Syndrome type 2, OMIM: 145410 Teebi hypertelorism Syndrome, OMIM: 145420	Exomphalos One patient of two presented with exomphalos. 1 patient of 16 presented with exomphalos, 3 with an umbilical hernia. One patient of two presented with an umbilical hernia	*Specc1l* ^CCD2/CCD2^	167, 230, 231, 232, 233

Clinical presentation	Human syndrome(s)	Comments	Potential mouse model	Reference
Fully open ventral (anterior) body wall with or without cover Exomphalos	Thoraco‐abdominal Syndrome Pentralogy of Cantrell OMIM: 313850 Fryns Syndrome OMIM: 229850 Gershoni‐Baruch Syndrome OMIM: 609545 Lethal omphalocele‐cleft palate syndrome OMIM: 258320 VACTERL syndrome OMIM: 192350 V (vertebral anomalies), A (anal atresia), C (congenital heart disease), TE (tracheoesophageal fistula or esophageal atresia), R (reno‐urinary anomalies), and L (radial limb defect). Shprintzen‐Goldberg Exomphalos Syndrome OMIM: 182210	Linked to chromosomal abnormalities of Xq25‐q27. Exomphalos observed sufficiently frequently in study of 524 infants with VACTERL syndrome it was proposed as an extended clinical presentation. Review by Kim, Kim, and Hui (2001)^234^ implicate SHH signaling in this syndrome. Two of four family members presented with exomphalos and supported by a further patient study. Also linked to microdeletions in chromosome 22q11.^235^	*Tfap2α* null/chimeric mice, Transgelin cre‐ *Tgfr*β2 null (see Table 2) *EphB/EphrinB* Mutants *SHH/Gli3/Alx4* mutants (see Table 2) *Pcsk5* null (see supplementary table)	56, 58, 97, 99, 236 102, 181, 234, 237, 238 235, 239, 240, 241, 242
Multiple	Limb–body wall complex (LBWC), pentalogy of Cantrell (POC), Exomphalos exstrophy‐imperforate anus‐spinal defects (OEIS) complex, vertebral‐anal‐cardiac‐tracheoesophageal fistula‐renal‐limb (VACTERL) association	**Nicotinamide adenine dinucleotide (NAD)** is a cofactor for ATP production in cells and a substrate for enzymatic reactions in metabolism, generated by tryptophan amino acid catabolism or dietary niacin metabolism. Deficiency of NAD in its oxidized form (NAD+) is proposed to be associated with multiple human syndromes. *Haao* gene is involved in metabolism of NAD+	Loss of function of *Haao* gene, dietary restriction of tryptophan and niacin led to exomphalos and gastroschisis in mice.	243, 244

*Note*: Mouse mutant models are included where relevant, for further details, see Table 2 and supplementary table.

### 3.7 Cell movements and body wall closure

Growth of the SBW is predominantly ventrally but there is also expansion in the thickness of the body wall at the midline and expansion along the cranial‐caudal axes. Cell rearrangements and migrations contribute to differential growth along these axes. Analysis of cell behaviors in the embryonic mouse thorax at E12.5 reveals that presumptive sternal cells, which are located at the leading edge of the SBW (within the sternal band, see Figure 2A and Section 4.2.2), are preferentially orientated along the cranial‐caudal axis and show no polarity, that is, cell protrusions are randomly oriented.^8^ However, by E13.5, the orientation of the cell long axis has changed: now cells are orientated horizontally across the dorso‐ventral axis. Live imaging of VBW explants has shown that there is cell intercalation across the dorso‐ventral axis which narrows the presumptive sternum while increasing sternal dimensions along the other two axes.^8^ These cell intercalation movements which change the shape of a tissue are known as convergent‐extension and in the sternal mesenchyme are controlled by the protocadherins, Fat4, and Dchs1. In *Fat4* and *Dchs1* mutants, these cell movements fail to occur and the sternum is wider, thinner and shorter.^8^ Similar patterns of cell movement have been observed for myofibroblasts at the leading edge of the thoracic and abdominal SBW.^99^ Whether convergent‐extension movements occur in regions other than the leading edge of the developing SBW, and/or within the PBW, is unknown.

There is also significant growth along the cranial‐caudal axis, together with midline merging, of the infra‐umbilical region. At CS17 (6 weeks) of human development, the UR and cloacal membrane are adjacent but from CS18 to week 9 a burst of cranial‐caudal oriented growth dramatically separates the two.^245^, ^246^ This is linked to descent of the abdominal muscles such that both their cranial and caudal attachments move caudally. The oblique abdominal muscles also shift medially.^246^ These authors also report the descent of the sternum at around the same time, which may reflect a broad caudal movement of the VBW as the lower abdomen grows. In mice, fate labeling and extirpation studies have demonstrated a similar caudal movement of infra‐umbilical mesoderm at E12 which contributes to growth of the VBW and the dorsal genital tubercle (GT).^50^ In a mouse mutant (*Alx4* ^*Lst/Lst*^) where this cell migration is defective, the GT is hypoplastic by E11.5.^50^ This indicates that this caudal migration must start earlier before E11.5. As well as growth along the cranial‐caudal axis, these cell movements are also linked with the merging of paired genital swellings. The two genital swellings are generated from the LPM and are initially located on each side of the cloacal membrane following embryonic folding.^247^, ^248^ Subsequently, the two genital swellings merge at the midline to overlie the cloaca. Signals from the cloaca, such as Fgf and Shh, also control development of the genitalia.^248^, ^249^, ^250^, ^251^, ^252^ The physical proximity, the shared morphogenetic processes and signaling interactions explain why caudal VBW anomalies such as bladder and cloacal exstrophy (CE) are often associated with genital anomalies (see Sections 5.2.5 and 6.5).

There is also additional evidence of considerable cell movement along the cranial‐caudal axis at the midline. DiI labeling of the thoracic LPM in stage 20 chick embryos (equivalent to E10.5 mouse embryos) shows that LPM expands along the cranial‐caudal axis and by day 10 the labeled cells are located as a line along the fused ventral midline of the embryo.^19^ How this extensive cell movement is directed is unknown.

## 4 MOLECULAR REGULATION OF VBW CLOSURE

Several signaling pathways and cellular processes have been identified as being essential within the PBW or SBW for VBW closure. Amongst these are the Tgfβ, canonical Wnt, Rho/ROCK signaling pathways and the transcription factors, Tfap2α and Six5/6. Six5/6 is essential within the PBW while Tgfβ and the canonical Wnt signaling pathways are required for SBW closure (β‐catenin and Wnt signaling references in Table 2). Pitx2 and Tfap2α are required for both PBW and SBW development.^56^, ^80^, ^81^, ^253^ These pathways control cell proliferation, survival, migration, differential cell adhesion and differentiation. ROCK signaling, which regulates the actin cytoskeleton and presumably, cell and tissue mechanics, appears necessary for the final step of body wall closure. Additionally, there are collective organized cell behaviors which change the relative dimensions of the thoracic body wall and are determined by the planar cell polarity (PCP) pathway, Fat4/Dchs1, as previously discussed (Section 3.7). The better characterized signaling pathways involved in VBW closure are discussed below and in Section 6. Additional genes that are essential for VBW closure are listed in Table 2 together with information about their potential roles, where known. Additional information about the mouse mutants and their phenotypes is also presented in Table S1.

In the following section, PBW formation will be discussed (Section 4.1) followed by consideration of how signals from the PBW influence SBW formation and molecular mechanisms of SBW development (Section 4.2).

### 4.1 Formation of the PBW

Understanding the folding of the early LPM forms the basis of understanding PBW development. This folding initially requires the co‐ordinated interdependent morphogenesis of the LPM (somatopleure, splanchopleure), endoderm together with the extra‐embryonic tissues which must move together (Figure 1A). During the later phase of VBW closure, the somatopleure that will form the PBW develops independently of the splanchopleure (Figure 1C). In the mouse, folding starts cranial‐caudally at the AIP at approximately E8 (Figure 1A). VBW defects due to abnormal PBW development may arise by failure in folding and development of the PBW due to defective development of one of the PBW tissue layers (see below). Alternatively, VBW defects may arise due to an alteration in signals from the PBW that affect SBW development (e.g., Tgfβ2, see Sections 4.2.1 and 4.2.3). In mice, phenotypes that are due to defective development of the PBW will be apparent by E11.5. Phenotypes due to defective signaling from the PBW will be apparent later. In the following section, we discuss key genes that are essential for different phases of PBW development in mouse embryos.

One of the first transcription factors seen in the LPM around the timing of embryonic turning at E7.5/E8.5 is the zinc finger transcription factor Gata4. There are particularly high levels of *Gata4* expression in and around the AIP.^254^, ^255^, ^256^, ^257^ Within these domains, Gata4 transcriptionally activates *Bmp4* expression in the LPM, which in turn positively regulates *Gata4* expression.^258^, ^259^ Lineage tracing experiments have confirmed that LPM cells in the PBW are derived from the early Gata4 expressing cells.^254^, ^257^, ^260^ *Gata4* null mice arrest development around the time of ventral folding with the defect apparent by E8. This model clearly shows failure of the LPM to fold ventrally, such that the embryo grows outside the yolk sac.^256^ Chimeric embryos made with *Gata4* null and *Gata4*‐expressing cells have indicated that Gata4 function in the endoderm, but not the LPM, is essential for ventral folding.^261^ This conclusion is also supported by specific deletion of Gata4 in the LPM. In these mice, the PBW forms but is thinner by E13.5.^254^ As Gata4 is only transiently expressed in the somatopleure up to E8.5, this must reflect an early role of Gata4 during establishment of the PBW.^257^

The *Gata4* null mouse resembles the *Furin* null mouse where there is also arrested cranial‐caudal and lateral folding which is clearly apparent by E8.5.^107^ In *Furin* null embryos, an additional phenotype has been reported in the allantois which is highly vacuolated and fails to fuse with the chorion.^107^ Furin is an intracellular processing protein which is also expressed in the AIP, LPM and extra‐embryonic tissues at E7.5 to E8.5. One of the roles of Furin is to cleave and activate Tgfβ family members, including Bmp4.^262^ However, despite the resemblance of the *Gata4* and *Furin* null embryos, the phenotype is not the result of loss of Gata4 as a secondary consequence of decreased Bmp signaling. In *Furin* null mice, *Gata4* expression is only absent is in the allantois but other domains of *Gata4* expression persist.^107^

The basic helix–loop–helix transcription factors, *Hand1* and *Hand2*, are highly expressed in the LPM and also extraembryonic tissues between E7.5 and E9.0.^263^, ^264^, ^265^, ^266^ In the post‐turning embryo *Hand1* and *Hand2* expression in the VBW is largely lost, with *Hand1* expression becoming confined to the umbilical cord region by E11.5.^263^, ^264^, ^265^ Lineage tracing experiments have confirmed the Hand1‐expressing cells give rise to the PBW.^69^, ^263^ Embryos null for *Hand1* or *Hand2* fail to complete the folding process, and arrest development by E9.5 with a phenotype where the PBW does not form and the VBW is open, resembling TAS VBW anomaly. Selective deletion of *Hand1* in the LPM results in a spectrum of VBW phenotypes ranging from an enlarged UR in pups to a gastroschisis (GS) type of VBW anomaly with the herniating gut right of the umbilical cord, lacking any tissue cover.^69^ This indicates that expression of Hand1 within the extra‐embryonic membranes may be essential for embryonic folding while expression within the LPM is needed for later closure events.^69^

Loss of the paired‐homeodomain transcription factor *Pitx2* can also disrupt early turning in some embryos.^80^, ^81^, ^228^, ^253^ However, in all embryos, Pitx2 is essential to establish the appropriate geometry of the somatopleure once folding has started. In *Pitx2* mutants, the left forming part of the VBW is bent outward, instead of bending medially toward the opposing right body wall at E9.5.^80^ The left LPM and adjacent amnion are also thickened, possibly mechanically restricting bending.^80^ This asymmetric anomaly presumably reflects the asymmetric expression of *Pitx2* within the early LPM.^79^, ^80^, ^116^ Analysis of the LPM in E10.5 *Pitx2* mutants has identified loss of the expression of some members of *Hox* (9, 10, 11 paralog groups) and *Tbx* family members (Tbx4, ‐15), while expression of other *Tbx* genes is either increased (Tbx6) or they are ectopically expressed (Tbx1, ‐2, ‐5).^82^, ^83^ Hox and Tbx genes determine regional identity of cells within an embryo indicating that the specification of the LPM has been disrupted. *Pitx2* is a transcriptional target of canonical Wnt signaling and in turn, there are also changes in the levels of components of the canonical and Wnt PCP pathways which would be predicted to alter cell proliferation/survival and co‐ordinated cell behaviors, respectively.^82^, ^83^, ^84^, ^267^

The sine oculis homeobox transcription factors 4 (Six4) and 5 (Six5) show expression in all three layers of the PBW where they play key roles in each layer: ectoderm, mesoderm, and coelomic epithelium. *Six4* ^−/−^;*Six5* ^−/−^ mice show a remarkable “pure” exomphalos anomaly, the thoracic midline closes completely and portions of the liver and intestine can be seen herniating through the VBW covered by a thin sac that has the umbilical cord at its center.^86^ In this double mouse mutant, the anomalies in the PBW appear at approximately E9.5 of development and are restricted to the abdominal VBW.^86^ There appears to a general failure to narrow the UR which is linked to an accumulation of cells at the lateral edge of the UR, together with decreased proliferation and increased apoptosis in the ectoderm and mesoderm at the lateral edges of the PBW.^86^ Additionally, differences in the morphology of the coelomic epithelium have been observed in *Six4*/*5* double mutants. Overexpression of Six4 within the coelomic epithelium promotes epithelial‐mesenchymal transition (EMT) suggesting that Six4 and 5 may also regulate EMT and contribute to thickening of the body wall.^86^ The selective restriction of the defect to the periumbilical region and the normal closure of the thoracic midline suggest that Six4/5 are required in differential growth of the right and left hemi body walls in a restricted region in the abdomen rather than being essential in the development of the whole PBW.

In summary folding of embryo to form the PBW requires the transcription factors, Gata4, Hand1/2, Pitx2, and the metalloproteinase furin1.^56^, ^79^, ^80^, ^81^, ^107^, ^116^, ^253^, ^255^, ^256^, ^264^ As the PBW is being generated, there are cell migrations that contribute to the thickening of the PBW. These cell migrations do not occur appropriately in *Tfap2α*, *Pitx2*, and *Six4/5* mouse mutants resulting in either a failure to close the PBW (Pitx2) or a thinner PBW which is vulnerable to rupture (Tfap2α), or contributes to a failure to close the SBW (Six4/5).^56^, ^80^, ^86^

At E9.5/E10 in the mouse, the PBW is demarcated from the forming SBW by the transcription factors Agouti, Alx3/4, Msx1/2, Hand1/2, Pitx2, Six5/6, and Tbx5.^38^, ^51^, ^74^, ^77^, ^79^, ^86^, ^92^, ^263^, ^268^ The PBW provides a physical and signaling framework upon which the fully differentiated VBW will be established by infiltration of cells from the SBW (Figure 2A–C). The ultimate fate of the PBW needs to be resolved. Chen et al. reported degeneration of the PBW as the SBW invades.^269^, ^270^ However, the persistence of a thin membranous cover, in many mouse mutants, where there is a failure of SBW formation (e.g. *Tgfβ*, see Table 2) indicates at least part of the PBW may normally persist.

### 4.2 The SBW

#### 4.2.1 Signals from the PBW control SBW development

Signals from the midline control development of the SBW (Figure 2A). First, signals from the LPM, including Bmp4, help establish the hypaxial musculature, and timing of differentiation, within the early somite.^271^, ^272^ Subsequently, myotomal extension is controlled by Wnt signaling from the PBW (together with signals from the SBW ectoderm).^39^, ^116^ Tgfβ signaling from the PBW ectoderm is also required for function of myofibroblasts, a critical cell population within the closing body wall^99^ (and see Sections 4.2.2 and 4.2.3). In the chick, Bmp signals from the PBW ectoderm and mesoderm also control rib extension.^18^

#### 4.2.2 The leading edge of the SBW is essential for VBW closure and is unique

In the thoracic VBW, the ventral edge of the SBW is demarcated by a band of *Sox9*, *Runx1 & 2*, *Tbx5* and *ephrinB1/B2* expression which in mice probably demarcates all the LPM (Figure 2A–C).^4^, ^19^, ^102^, ^104^ This is histologically visible as a denser group of cells at E12 and has been termed the developing sternal band.^273^ This domain moves ventrally as the SBW expands (Figure 2A–C). *Tbx* gene family members (*Tbx1*, *‐2*, *‐4*, *‐5*, *‐6*, and *‐15*), *Hic1* and *ephrin B1/B2* also demarcate the leading ventral edge of the abdominal SBW and this domain is known as the abdominal band and is a subdivision of the LPM (Figure 2C).^73^, ^84^, ^102^, ^104^ These ventral leading edges consist of a heterogeneous cell population of different cell origins which includes myofibroblasts and are associated with the cutaneous nerves.^35^, ^99^ Based on their histological spatial relationship ahead of other differentiating components of the VBW, the sternal and abdominal bands are proposed to be critical regulators of VBW closure.^20^, ^26^, ^35^ Indeed, some of the genes (*Runx1/2*, *Hic1*, *ephrin B1/B2*) within this leading edge are essential for VBW closure (Figure 2A,C; Tables 2 and S1). The myofibroblasts within this domain are also essential for body wall closure^99^ (and see Section 4.2.3). *Tfap2α* is highly expressed in the ectoderm overlying these mesenchymal domains (Figure 2C).^56^ Curiously, histological analysis has shown that the abdominal band, and associated cutaneous nerves, are absent in *Tfap2α* mutants which are characterized by an open body wall with disorganized connective tissue and delayed muscle differentiation.^56^

#### 4.2.3 TGF‐beta signaling and the role of myofibroblasts

Aldeiri et al. reported a migrating population of Transgelin (Tagln, also known as SM22α)‐expressing myofibroblasts within the thoracic and abdominal VBW.^99^, ^100^ The myofibroblasts appear in the dorsal body wall by E10.5 and then advance toward the ventral midline. By E11.5, they fill the breadth of the PBW and are also found at the leading edge of the SBW ahead of the developing sternum, ribs, and hypaxial musculature.^99^ By E15.5, they are positioned at the ventral midline, staying in this location into the post‐natal period.^99^ These are presumably the same cells as the αSMA populations identified between the two opposing sternal bars as they meet at the midline at E14.5.^49^ Aldeiri et al. proposed that they are a pioneering cell population instrumental for VBW closure.^99^ Whether myofibroblasts function by paracrine signaling to adjacent tissues or act mechanically is still unclear.

The myofibroblasts express Tgfβr2 and migrate ventrally in response to Tgfβ2 from the PBW ectoderm (Figure 2A).^99^ Conditional inactivation of Tgfβr2 within the Tagln‐expressing cells (but not myogenic or chondrogenic cells) results in a Pentalogy of Cantrell (PC) phenotype which is characterized, in part, by the failure to close both the thoracic and abdominal VBW (see Section 6.1). This clearly demonstrates that Tgfβr2 signaling within the myofibroblasts is essential for VBW closure.^99^, ^100^ However, the phenotype does not include bladder exstrophy indicating there are different cellular and/or molecular mechanisms of closure of the lower (caudal) abdominal VBW. It is currently unclear if this population of cells is the same or includes the population of invading “undifferentiated spindle‐shaped cells” identified at the forefront, that is, leading edge of the SBW in both mouse and chick embryos.^20^, ^269^, ^270^ Chen hypothesized that this cell population drives VBW closure and demonstrated that the leading edge of the SBW is able to close in an ex‐vivo explant culture in the absence of adjacent tissues.^26^ The Tagln migratory fibroblasts have been proposed to arise from the somite and might also overlap with the somite (sclerotome and syndetome) derived *Hic1*‐expressing cells.^72^ *Hic1* expressing cells are found at the leading edge of the SBW, spatially overlapping with Tagln (Figure 2C).^70^, ^72^, ^99^ *HIC1* is deleted in Miller‐Dieker Syndrome, a contiguous gene disorder, where exomphalos can occur (OMIM 247200). Moreover, the *Hic1* ^*−/−*^ mouse mutant can exhibit exomphalos, although this phenotype is not fully penetrant.^73^

#### 4.2.4 The Wnt pathways are key regulators of SBW closure

Wnt signaling in both the ectoderm and mesenchyme is required for appropriate VBW closure.^49^, ^116^, ^117^ Wnt signaling includes a canonical pathway, which controls cell proliferation, survival or differentiation, and PCP pathway components which control co‐ordinated cellular behaviors or cellular characteristics across a plane of tissue.^274^, ^275^ Core components of the canonical pathway include the transmembrane proteins, LRP5/6 and the intracellular effector molecule, β‐catenin. The Wnt‐PCP pathway includes Vangl1/2, Ror1/Ror, and Ryk proteins. Loss of function of both canonical and Wnt‐PCP components result in VBW closure anomalies highlighting the crucial role of both branches of the Wnt signaling network. Defects in Wnt signaling include open thoracic and abdominal VBWs, a thin tissue layer at the midline, and a split sternum.^39^, ^49^, ^116^, ^117^

Analysis of canonical Wnt reporter mice shows that canonical Wnt signaling is active within the midline of the VBW (Figure 2A).^39^, ^49^ In mouse embryos, following mesenchymal inactivation of β‐catenin or of LRP5/LRP6 co‐receptors, critical components of canonical Wnt signaling, the midline of the VBW remains thin and appears not to be populated by the SBW.^39^, ^49^ This phenotype can be explained by the three roles of β‐catenin within the VBW mesenchyme: survival, proliferation of mesenchymal cells and differentiation of the dermal fibroblasts.^39^, ^40^ Additionally, canonical Wnt signaling controls migration of the myogenic cells into the VBW.^116^ A transcriptional target of canonical Wnt signaling in both the mesoderm and developing musculature is Pitx2, also essential for VBW closure and muscle development^84^, ^267^ (see Section 4.1).

*Wnt5a* and *Wnt11*, which are typically linked with PCP pathway components are expressed in the VBW mesenchyme.^49^ In *Wnt5a* mouse mutants the thoracic VBW remains open while *Ryk* ^*−/−*^ */Vangl2* ^*−/−*^ and *Ror1* ^*−/−*^/*Ror2* ^*−/−*^ double mutants are characterized by exomphalos, where the abdominal VBW has not closed appropriately, and a lack of growth of the lower abdomen.^127^, ^128^ Coupled with limb anomalies, this phenotypic spectrum resembles limb–body wall complex (LBWC) (see Section 6.1). Based on the pathways function in other tissues,^274^ the Wnt PCP pathway is predicted to regulate convergent‐extension and oriented cell divisions but this has not yet been demonstrated in the VBW. Loss of Wnt‐PCP signaling results in a decreased rate of migration and ectopic exposure of Wnt5a increases cell migration suggesting Wnt5a may control collective cell migrations/movements in vivo.^49^

#### 4.2.5 Mechanical forces may pull the SBW closed

Mechanical forces include tensions and forces from neighboring cells and the matrix environment which acting together can influence cell behaviors such as orientated cell divisions, migratory behaviors and cell intercalations in addition to cell differentiation. For example, myofibroblast migration will be determined by mechanical factors, such as matrix organization and stiffness, in addition to chemotactic cues. Alterations in the matrix organization, and presumably stiffness/tension are also linked to VBW anomalies as illustrated by the *Bmp1*, *glucocorticoid receptor*, and *Alcp* mutants (see Tables 2 and S1). Intracellularly, these mechanical forces are mediated by alterations in the actin cytoskeleton via alterations in the activity of ROCK kinases and non‐muscle myosins, together with changes in transcriptional responses mediated, via, for example, Yap/Taz, canonical Wnt, and EGR pathways. Candidate intracellular mechano‐effectors that have a VBW phenotype when mutated are listed in the “cytoskeletal components” in Table 2. For example, loss of the actin cytoskeleton, through inhibition of ROCK, would decrease the ability of cells to respond to mechanical cues. *ROCK1* ^*−/−*^ and *ROCK2* ^*−/−*^ mouse mutants are characterized by exomphalos but the penetrance of the phenotype is dependent on genetic background,^44^, ^165^, ^166^, ^276^ In wild‐type E16.5 embryos, filamentous actin is present at the UR, a region of thickened ectoderm between the body wall ectoderm and amnion. In *ROCK1* ^*−/−*^ mutants, this thickened ridge of ectoderm is absent and there is decreased actin staining.^44^ As the actin cytoskeleton responds to, and can drive, mechanical changes this suggests that the actin cytoskeleton narrows and closes the umbilicus. Chemical inhibition of ROCK activity in early chick embryos at the stage of PBW formation results in body wall defects but the precise role of mechanical forces, and the actin cytoskeleton at this early stage of development is unknown.^277^ A mutation in non‐muscle myosin heavy chain B (NMHCII‐B^R709C^; NMHCII‐B also known as Myh10) that impairs motor activity results in VBW defects.^162^, ^163^ Again, as for other signaling components, there are differential sensitivities along the cranial‐caudal axis. Heterozygous NMHCII‐B^R709C^ mice have exomphalos while homozygous NMHCII‐B^R709C^ mutant mice exhibit both thoracic and abdominal closure defects.^162^, ^163^

## 5 CLINICAL VARIANTS OF BODY WALL CLOSURE DEFECTS

### 5.1 Introduction

The clinical correlates of the embryological pathophysiology so far described are imperfect but form a necessary translational aspect to the science. We will consider this with a detailed exploration of factors that have been implicated in the clinical condition and a brief description of the various conditions broadly encompassed by the term—abdominal wall defect (AWD).

### 5.2 Clinical correlates

#### 5.2.1 Ectopia cordis

There are less than 100 cases in the literature^278^ and it can be divided into two broad categories: those where only the heart protrudes from the thoracic cavity and those where it forms part of a more syndromic entity (e.g., PC, considered below). There is no apparent racial variation, and its incidence has been quoted at 5 per 10^6^ births. Early antenatal diagnosis should be possible from the 12th week of gestation and the usual outcome would be elective termination. For those few carrying to term, a successful repair is seen to be much more likely in those forming part of a Cantrell syndrome. The first successful repair of an isolated ectopia cordis only occurred in the 1970s involving a staged process of initial skin flaps and later actual reduction into the thoracic cavity with fashioning of sternum and rib cover. There are very few long‐term survivors though.

#### 5.2.2 Syndromic AWDs

The principle syndromic midline AWDs of PC, Thoracoabdominal Syndrome (TAS), and the LBWC can be considered together. Exomphalos (omphalocele), bladder and cloacal exstrophy, imperforate anus, spina bifida (OEIS) will be considered in a later section.

James Cantrell et al. from Baltimore described 5 cases and added 21 cases from the literature^279^ of an association of five related anomalies (exomphalos, diaphragmatic defect, pericardial defect, sternal cleft, ectopic cordis [or intracardiac defects]) in 1958. Much of the subsequent literature has been based on case reports with some exceptions^280^ so there is very little actual consistent evidence for causation. The only registry‐based estimate of incidence is 1 in 200,000 live‐born infants.^281^

One does not require all five components; indeed, the ectopia cordis element is now extraordinarily rare but at the very least there should be epigastric exomphalos, central diaphragmatic defect and perhaps absence of xiphisternum/cleft sternum. Genetic factors may be important in a few, with a number of trisomies reported (21, 18) as well as Turner Syndrome, with one report identifying concurrence with the Goltz‐Gorlin syndrome (characteristic facies, anophthalmia, cleft lip and palate; limb malformations and dermal defects).^225^ This latter condition has been linked to an X‐linked (Xp11. 23) *PORCN* (Porcupine) mutation in almost all cases, that has an important role in mediating Wnt ligand palmitoylation^282^, ^283^ (see Section 6.1).

**TAS (OMIM 313850)** is characterized by X‐linked midline defects that include diaphragmatic and ventral hernias, hypoplastic lungs, and cardiac anomalies and was first identified in an Israeli family^236^ with the TAS gene mutation being mapped to the region of Xq25–q27.

**LBWC** is very rare and fatal (usually intrauterine, sometimes as a stillborn birth) with a wide range of phenotypic pathologies including exomphalos together with cranio‐facial anomalies (exencephaly, encephalocele, facial clefts); and limb defects (e.g., talipes, absent limbs, oligodactyly, sometimes with features of amniotic bands).^284^, ^285^ There have been no defined genetic or predisposing factors, beyond a report relating two LBWC fetuses to heavy use of smoked crack cocaine in the mother.^286^

#### 5.2.3 Gastroschisis

GS is probably the commonest AWD and is very familiar to all pediatric surgeons. There is a full‐thickness AWD almost always to the right of the normally‐inserted umbilicus. It is an isolated defect in the vast majority and those associated anomalies are mostly caused by the actual defect itself (e.g., midgut deletion, gastrointestinal atresias, undescended testes).

The actual timing of the defect is not known with any certainty and its cause remains entirely speculative. One recent suggestion^287^ is that the developing cord normally has two parts, a firm left‐sided part formed by the vessels and urachus, and a thin right‐sided pouch covering the intestinal loops (the “physiological umbilical hernia”), which is at risk of rupture. That is, this is a secondary event following completion of the abdominal wall occurring between 10 and 14 weeks gestation. There is then intestinal prolapse led by the midgut with increasing hernial ring dilatation. Nonetheless this ring can contract later pinching off the midgut and causing ischemic compromise and even total infarction—so‐called “closed GS”—with devasting consequences.^288^

#### 5.2.4 Exomphalos (aka omphalocele)

Exomphalos may be defined by midline absence of abdominal wall fascial integrity but with preservation of a sac covering the protrusion of the intra‐abdominal viscera. There is wide separation of the rectus muscles around the defect. Clinically we recognize a spectrum based on size of defect and intactness of sac but clinically categorize into Major (>5 cm diameter defect ± presence of liver within sac) and Minor (<5 cm diameter) with two smaller categories at either end—Giant (based more on size of sac rather than defect diameter) and the small “hernia‐in‐cord” which appears as a rather thickened insertion of the umbilicus and a visible bowel loop within. Umbilical hernias are post‐natal in origin related to failure to complete the cicatrization process at the level of the insertion of the ligated umbilicus and will not be considered further.

Exomphalos (typically major) are associated with various trisomies (mainly 18, 13) and gene mutations consistent with the Beckwith‐Wiedemann syndrome (BWS) (located on chromosome 11p15)^289^ that have an association with exomphalos minor. Associated non‐syndromic anomalies are not uncommon. The British Association of Pediatric Surgeons Congenital Anomaly Surveillance System reviewed all 162 infants with exomphalos born during the 2‐year period March 2014 to February 2016 in the United Kingdom and Ireland. Most of these had other congenital anomalies and could be classified as syndromic (e.g., trisomy 18) (*n* = 15, 9%), other structural anomalies (*n* = 72, 44%) and the BMS (*n* = 29, 18%). Only 46 (28%) infants could be described as having an isolated exomphalos (personal communication). Furthermore, lung hypoplasia can be clinically important and relates to the size of the defect and negative impact of extra‐abdominal viscera on the developing lungs.

#### 5.2.5 Classic bladder exstrophy, cloacal exstrophy (CE), and OEIS

Lower midline AWDs include **bladder exstrophy** and **CE** with the most severe defect along the spectrum being termed **OEIS complex** (OMIM 258040). Classic bladder exstrophy has a number of related features with failure of the ventral aspect of bladder and abdominal wall; a split pubic symphysis and divergent rami; and epispadias in boys. CE similarly has fusional failure of the lower abdominal wall with a small exomphalos above an opened out undifferentiated cloaca consisting of a central caecal plate with two template hemi‐bladders on either side.^290^ There is usually a prolapsed distal ileum giving a characteristic “elephant's trunk” appearance. A hindgut orifice on the caecal plate leads to a blind anorectum. Genitalia in both sexes are invariably bifid and hypoplastic with vaginal duplication/agenesis and uterine duplication in girls and a bifid phallus and undescended intrabdominal testes in boys. Indeed, in 46XY boy's phallic structures may be so hypoplastic as to preclude anatomical and functional penile reconstruction leading to gender reassignment (certainly the historical preference). OEIS involving not only ventral but dorsal midline defects was first defined by Carey et al.^291^ and with increasing appreciation of the degree of spinal involvement (dysraphism, tethered cord syndrome, myelomeningocele) nowadays is almost used synonymously with CE.^290^ CE has an estimated incidence of about 1 in 130,000 births^194^ with up to 20% of them having defined features of OEIS.

#### 5.2.6 Prune belly (aka Eagle Barrett syndrome, Prune Belly Association)

Prune belly syndrome (PBS) has been characterized by deficient or absent abdominal wall musculature, hypotonia, together with pathological dilatation of the urinary system and (in males) bilateral intra‐abdominal testes (it rarely occurs in females). Its unusual name was coined by the Canadian physician William Osler in 1901 describing its most obvious abdominal wall appearance. Most present antenatally with hydronephrosis, a low‐pressure distended bladder and oligohydramnios. Severe degrees of renal impairment and accompanying lung hypoplasia are poor prognostic features with perinatal mortality between 10 and 25%.

### 5.3 Environmental/epidemiological aspects

The epidemiology and influence of environmental factors have only really been studied in the two principle AWDs of exomphalos and GS, the others being far too rare for consideration.

#### 5.3.1 Epidemiology of AWDs

Since the 1960s, the distinction between the GS and exomphalos (synonymous with omphalocele) had been recognized and while the prevalence of exomphalos has remained static (with caveats—see below) that of GS has been consistently rising at least in Western societies^292^, ^293^ although there may be some recent evidence of a leveling off.^294^ The reasons for this remarkable observation are essentially unknown. So, the current incidence in the United Kingdom varies from 1 in 3000 to 1 in 8000 live births, with the higher values seen in urban industrial areas.^295^ The incidence of exomphalos has plateaued since the 1970s, being 1 in 2600 in one large UK study (2005–2011)^296^ with a mean maternal age at delivery being 29.2 years (same as controls). Certainly, by contrast, young maternal age does appear to be a significant factor in GS.^294^, ^297^ In one Californian study mothers <16 years of age had 4.2‐times greater prevalence of GS than mothers between the ages of 25 and 29 years.^292^

Ethnicity, likewise, appears important with white Caucasians and native Americans having a higher risk of GS than either black or those of Hispanic origin in the United States.^292^, ^297^ Finally, lower household income or social group status may also have an effect in GS.^297^

The prevalence at birth of exomphalos, however, masks a considerable loss during intrauterine life. This is not only naturally, but also iatrogenically with the advent of almost universal screening maternal ultrasound and ability to define severe genetic abnormalities at increasingly earlier gestational ages. As an illustration of this, Lakasing et al.^298^ from King's College Hospital, London reviewed the outcomes of 445 fetuses with exomphalos; 56% of which had a defined chromosomal anomaly, 30% had a normal karyotype (remainder not karyotyped due to parental request). Fetuses with chromosomal anomalies and other potentially severe anomalies were usually offered termination. Ultimately, 55 (18%) were actually live‐born.

#### 5.3.2 Environmental factors

In general, these are more well‐established albeit by no means proven for GS than for exomphalos.

##### Gastroschisis

There have been associations with maternal drug use both illegal (e.g., cocaine, heroin) and legal (e.g., aspirin, ibuprofen).^297^ Most of these studies rely on self‐administered questionnaires and recall, which clearly has its own limitations, but one study from Leicester, UK^299^ used maternal hair analysis to verify and confirmed a link with vasoactive recreational drugs (e.g., Ecstasy, cocaine; OR = 3.3, 95% CI: 1.0, 10.53) and a very marked association with 1st trimester aspirin (OR = 20.4, 95% CI: 2.2, 191.5). Nevertheless, some recent population studies from California,^297^ and London^300^ have disputed this. The latter study used the same hair analysis technique and initially showed the same relationship for recreational drug use (25% vs. 13%) but the difference became less significant when corrected for maternal age (25% vs. 21%). By contrast, the incidence in the exomphalos group was 7% throughout. There have been other environmental factors suggested as of significance in GS but most remain speculative. Thus, Torfs et al.^301^ suggested that maternal occupational exposure to solvent use (e.g., paints, glues) had an association, though this did not stand up to analysis in a large study based on the National Birth Defects Prevention database.^302^ Similarly, a small Welsh case–control study^303^ suggested that a higher intake of fruits and vegetables during the first trimester and higher body fat percentage were associated with a reduced risk of GS and confirmed an earlier link with maternal smoking. Perhaps more contentiously, some studies have suggested an association with the use of some agricultural herbicides (specifically Atrazine).^304^

##### Exomphalos

Many studies have discriminated between syndromic and non‐syndromic exomphalos implying more genetic issues in the former. So in a large American National Birth Defects Prevention Study^297^ (*n* = 168 [non‐syndromic], 4967 controls), infants tended to be male, arising as part of a multiple birth and less likely to be born to a multipara woman. Women were more likely to have consumed alcohol (OR = 1.53; 95% CI, 1.04–2.25), and to be heavy smokers (OR = 4.26; 95% CI, 1.58–11.52) than controls.

Atmospheric pollution has been suggested as a possible causative agent in the etiology of congenital anomalies^305^ and, specifically sulfur dioxide pollution has been linked in one study from Liaoning Province, China^306^ with a modest increased prevalence of exomphalos (OR = 1.39, 95% CI = 1.22–1.65).

## 6 ANIMAL MODELS OF VBW DEFECTS AND RESEMBLANCE TO HUMAN PATHOLOGY

VBW anomalies can be caused by changes in a variety of cell behaviors, as outlined in Sections 2 and 3. These defects may occur during the initial folding of the somatopleure and splanchopleure, at the lateral, cranial and caudal edges, or during subsequent growth and morphogenesis of the VBW. Most of our knowledge on VBW closure, and the mechanisms that govern its anomalies, is derived from animal models of VBW closure defects. While these models provide insight into human pathology, it is inappropriate to draw exact parallels between the anomalies in mice and humans. The more severe forms of ventral closure defects encountered in human embryos are rarely an isolated pathology and are generally associated with other genetic or structural abnormalities. These cases do not survive to a live birth or undergo termination of pregnancy. Hence, the clinical phenotype is not well understood and it is estimated that at most 10% of embryos identified to have an AWD will survive to birth.^298^ Hence, a viable human VBW pathology represents the milder forms of VBW closure defects in comparison to animal models. Nevertheless, the process of VBW closure is largely preserved in mammals, and animal models of closure defects provide a platform to study these anomalies in humans. This section will focus on animal models of VBW closure defects analyzed from a human pathology narrative. We will discuss key pathways that are linked to VBW closure anomalies in mice, crucial cellular and molecular mechanisms, and their relevance and representation in human pathology. The following discussion refers to mouse models unless stated otherwise. Additional animal models are listed in Table 2 (also see Table S1 for details of each mouse mutant and their phenotypes). Human gene associations and chromosomal changes frequently linked with clinical VBW pathologies are listed in Table 3.

### 6.1 Syndromic AWDs

Exomphalos (omphalocele) is a known association in many human syndromes, the most common are BMS, trisomy 13 (Patau syndrome), trisomy 18 (Edwards syndrome), and PC. It is also reported, to a lesser extent, in focal dermal hypoplasia (FDH), Shprintzen Goldberg Syndrome, and many others. Animal models recapitulating some of these syndromes demonstrate a high degree of overlap in developmental phenotypes including VBW closure defects and are discussed below.

BWS is the most recognized human syndrome associated with exomphalos. Two specific genetic defects in the imprinting cluster regions 1 and 2 on chromosome 11p.15.5, lead to improper expression of different genes and result in BWS. These genes include IGF2 (insulin‐like growth factor II), H19, KCNQ10T1 (LIT1), and CDKN1C (p57[KIP2]). The H19/IGF2 and CDKN1C/KCNQ1OT1 clusters and their regulatory mechanisms are conserved in the mouse and are located at distal chromosome 7.^307^ Modeling BWS using a paternal uniparental disomy of chromosome 7 (PatDp(dist7)T9H‐0*/Tg*) or in mice lacking the imprinted Cdk inhibitor p57^KIP2^ resulted in VBW closure defect. These mutants display a spectrum of secondary VBW closure defects ranging from an exomphalos‐like anomaly to a thinned ventral skin and musculature, together with other developmental elements resembling BWS in humans.^157^, ^308^

PC is a constellation of ventral anomalies including the heart, diaphragm and the VBW. The exact etiology is unknown, and the majority of cases are sporadic. Genetic clues to the pathogenesis of PC are implied in familial cases through chromosomal abnormalities, such as Turner Syndrome, and in some reports of FDH (Goltz‐Gorlin syndrome).^225^, ^226^, ^236^, ^281^, ^309^ The full phenotypic spectrum of PC is emulated in a number of mouse models targeting the Tgfβ pathway, homeobox genes and non‐muscle myosin heavy chain type II (NMHCII encoded by the *Myh10* gene)^74^, ^97^, ^100^, ^162^, ^163^, ^310^ The Transgelin (Tagln)‐Cre Tgfβr2 null mouse, in which Tgfβ is inactivated within a SBW leading edge myofibroblast population, recapitulates all aspects of PC. These mice are characterized by ectopia cordis, anterior diaphragmatic hernia, a single outflow tract (truncus arteriosus) and ventricular septal defect, an absence of the diaphragmatic pericardium and finally exomphalos.^100^ The exact role(s) of Tgfβ signaling within the myofibroblasts is yet to be established. Similar constellation of the congenital defect can be observed when Homeobox genes are disturbed. The *Hoxb4* ^*PolII*^ homozygotes demonstrate ectopia cordis, failure of ventral abdominal wall closure, herniation of the liver to a thoracic position, failure of anterior pericardial sac formation and a varied spectrum of structural cardiac defects.^74^ The defect in the ventral midline appears to occur at early stages in SBW development, however, the exact mechanism affecting cardiac or diaphragmatic development remains unclear. Similarly, mice harboring a targeted mutation in an amino acid (Arg709 to Cys) in the motor domain of NMHCII‐B (*Myh10*) *B* ^*R709C*^ */B* ^*R709C*^ exhibit a number of midline closure defects, including of both the thoracic and abdominal ventral midline, anterior (ventral) diaphragm, and the palate.^163^ Yet, the cranial and infraumbilical VBW midline do develop fully. These mutants also display restricted cardiac atrioventricular cushion development resulting in a double outlet right ventricle. It is suggested that the *Myh10* gene defect results in reduced apoptosis, abnormal cell adhesion and ultimately failure of cells assembling at the midline.^163^

Murine models recapitulating FDH display some of the phenotypic elements of PC. In humans, loss of function mutations in porcupine (*PORCN*), an ER membrane O‐acyl transferase results in FDH.^311^ The key clinical features of skin hypoplasia, dental, and ocular anomalies, are seen in the null *Porcn* mutant.^118^ This model displays, to a variable degree, exomphalos and sternal hypoplasia, but not other features of PC. *Porcn* is dedicated to Wnt palmitoylation and secretion demonstrating the critical role of Wnt signaling during body wall closure. Moreover, secondary thoracic and abdominal ventral midline development fails when Wnt is disrupted in the ventral mesoderm or ectoderm.^49^, ^57^, ^117^

TAS and LBWC are fatal embryonic syndromes where the ventral midline lacks any tissue cover, and the thoracic and/or abdominal organs are exposed in the amnion. They are generally associated with a wide array of congenital anomalies that include the limb, neural tube and craniofacial defects. Given their rarity and lethality no study to date has identified a specific genetic causation in humans. Yet, TAS can readily be observed in the *Tfap2α* null mouse, *Grhl2* mutant, and *Pitx2* null mice.^56^, ^57^, ^58^, ^67^, ^81^ The expression of these genes in the ectoderm and LPM suggest their involvement in an essential epithelial‐mesenchymal pathway, including regulation of canonical Wnt and Wnt‐PCP signaling, that when lost prevents or halts SBW development at a very early stage^82^, ^83^ (also see Sections 4, 4.1, and 4.2.2). An interesting outstanding question raised by the *Tfap2α* and *Grhl2* models relates to the identity of the components of the proposed epithelial‐mesenchymal signaling pathway.

Interestingly, a phenotypic spectrum representative of LBWC is present in various murine models of disturbed PCP protein function most likely linked to non‐canonical Wnt signaling (Wnt‐PCP). This is most pronounced in the *Vangl2* ^*Lp*/+^; *Scribble* ^*Crc*/+^double heterozygote, and *Ryk* ^*−/−*^; *Vangl2* ^*−/−*^double null but also presents with very low penetrance in *Scrib* ^*crc/crc*^, *Ror1* ^−/−^; *Ror2* ^−/−^ double homozygote, and *Ptk7* ^*chz/chz*^. The constellation of developmental anomalies displayed by these mutants include a ventral midline closure defect, neural tube closure defect, structural cardiac anomalies, anorectal malformation, and a variable degree of skeletal and hind limb deformities.^120^, ^121^, ^127^, ^128^, ^312^ The pattern of congenital defects and the overall topographical appearance of these mutants is highly similar to reported miscarried human fetuses with severe forms of LBWC with the exception of the neural tube defect, exencephaly.^285^, ^313^ It is proposed that these various components of Wnt‐PCP signaling contribute to collective tissue movements, including convergent extension, which impact on embryonic turning and posterior axis elongation, possibly at an early gastrulation phase and also during later cranial‐caudal extension (see Section 3.7), leading to this unique pathology. A milder form of LBWC can be observed in the *Msx1‐Msx2* double knockout and the *Msx2‐Cre*; *Wls* ^*c/c*^ which prevents Wnt ligand secretion from the ectoderm. In both models, the secondary VBW fails to develop to a variable degree which is associated with fore‐ and hind limbs malformations.^77^, ^116^ As reported in human cases of LBWC, the VBW anomaly spares the thorax midline and is associated with bifid genitalia. However, the ventral organs in these murine models maintain a degree of mesenchymal‐epithelial tissue cover as opposed to the complete schisis nature of the anomaly in humans. Moreover, *Msx1/2*, and *Msx2‐cre*; *Wls* murine models are viable in late embryonic stages and fetal life, a rather unusual characteristic in human reports of LBWC.

One of the synonyms of LBWC is short umbilical cord syndrome, emphasizing that this constellation of defects is unique to the lower torso which again suggests an independent developmental mechanism such as disturbed mesenchymal cell migration restricted to the infraumbilical mesoderm (see Section 3.7).

### 6.2 Ectopia cordis and sternal cleft

Ectopia cordis is a rare and lethal embryonic malformation usually encountered as a component in PC, or rarely as an isolated pathology. The exact mechanism leading to this unique defect is rather obscure, however it has been suggested that failure of cranial body folding or decreased growth of the thoracic VBW, leads to herniation of the heart outside the pericardial cavity. There is no animal model to date for pure ectopia cordis, and most of our knowledge on the pathology is derived from models of severe ventral midline closure defects or those mimicking PC.^49^, ^74^, ^100^, ^117^, ^163^ Canonical Wnt signaling is essential for thoracic VBW closure and has been implicated in isolated ectopia cordis but this pathway is also essential for abdominal VBW closure.^49^, ^117^ The *Wls* ^*f/f*^; *Dermo1* ^*Cre/+*^ and *Lrp5/6* ^*f/f*^; *Dermo1* ^*Cre/+*^ mutants, which lack Wnt ligand expression (*Wls* ^*f/f*^; *Dermo1* ^*Cre/+*^) and intracellular canonical Wnt signaling (*Lrp5/6* ^*f/f*^; *Dermo1* ^*Cre/+*^) in the mesoderm, demonstrate failure of mesenchymal cell migration to the ventral midline from as early as E11.5.^49^ In the absence of Wnt signaling, there is decreased cell survival and proliferation. The targets of Wnt signaling include the *Pitx2*‐expressing and dermal cell populations (see Section 4.2.4). An additional question is whether myofibroblasts, a crucial cell population, also require Wnt signaling.

An isolated split sternum is not classified, at least clinically, under VBW closure defects. Nevertheless, there are many murine models displaying this defect, and they provide crucial mechanistic insight into sternum and thoracic VBW development and closure. A list of these models is provided in Table 2.

### 6.3 Gastroschsisis

GS is the most commonly encountered VBW closure defect and carries the best clinical outcomes, yet its etiology is not at all well understood. Despite the mechanistic insight obtained from a handful of murine models of GS, it is essential to note that the generation of a true and representative animal model of the pathology is yet to be seen. The sporadic, isolated and low complexity nature of the pathology, coupled with defined environmental associations renders it difficult to predict target genetic or mechanistic pathways. Some genetic mouse models show features of VBW closure defects suggestive of GS, as discussed below.^121^, ^133^, ^134^ The main debate regarding these models is that in human GS the eviscerated organs are free floating in the amniotic fluid. In murine models, the eviscerated organs sit in an exocoelomic space separated from the amnion, while the amnion maintains its attachment to the edges of the umbilical cord.^122^ This unique configuration could be explained by the fact that following completion of turning in the murine embryo, the intra‐ and extraembryonic coeloms are continuous at the edges of the umbilical cord. Here, the PBW is deficient and a mesenchymal failure, restricted to a susceptible portion of the mesenchyme, could lead to gut herniation into the extraembryonic coelom rather into the amniotic cavity. Hence, the herniated gut contents are covered by the thin membranous visceral yolk sac and are not in direct contact with the amniotic fluid, contrary to what is seen in human GS. Despite this dilemma, these models of VBW closure defects are more in keeping phenotypically with GS rather than an exomphalos.

Mouse mutant models presenting a GS‐like phenotype are scarce and can be observed mainly in the *Aclp* ^*−/−*^, *Bmp1* ^*−/−*^, *Barx1* ^*−/−*^, and to some extent in the *Hand1* ^*f/f*^:*Tlx2‐Cre*, *Shroom* ^*−/−*^ and *Folbp* ^*−/−*^ mouse mutants.^61^, ^69^, ^133^, ^134^, ^135^, ^147^, ^168^ *Bmp1* is expressed in the somatopleure of the VBW and in the mesoderm of the UR.^135^ The *Bmp1* mouse mutant lacks the ventral mesoderm within the umbilicus that is normally continuous with the peritoneal mesoderm.^135^ Similarly, selective deletion of *Hand1* within the PBW and LPM of the UR results in a GS phenotype.^69^ These studies suggest that a cell population in the UR mesoderm is involved in the pathogenesis of GS. Furthermore, *Aclp* and *Bmp1* both regulate collagen structure.^135^, ^314^, ^315^, ^316^ ACLP increases the tensile strength of collagen fibrils while Bmp1 cleaves the C‐terminus of collagen fibrils and also activates lysyl oxidase precursor that cross links collagen fibers.^135^, ^314^, ^315^, ^316^ In the amnion and VBW of E13.5 *Bmp1* mutants, the collagen fibers are defective and histologically resemble “barbed‐wires” rather than straight fibrils.^135^ Similarly, a small proportion of *Shroom* ^*−/−*^ mutants display a GS anomaly.^168^ Shroom is an F‐Actin binding protein and it is strongly expressed in the PBW from E9.5. *Shroom* mutants demonstrate defective actin sub‐cellular localization and irregular cytoskeletal architecture. All these data indicate that mechanical changes within the UR tissue may be a contributory factor in the pathology of GS.

Mammalian models of GS have also been obtained by exposure to teratogens including carbon monoxide, cadmium and retinoic acid (reviewed by^317^). The susceptibility to teratogens indicates there is a more sensitive tissue/domain within the developing VBW. The presence of a GS phenotype in the folate binding protein‐1 (Folbp‐1) model, and the partial recovery of the phenotype with folate supplementation further supports the presence of a susceptible region within the UR mesenchyme.^147^ This would also explain the high prevalence of GS in pregnancies exposed to vasoactive recreational drugs. This vulnerability may be due to a structural weakness such as a thinner tissue, regions of higher ROS activity with different metabolic activity, and/or be due to tissue remodeling. Indeed, apoptosis is high within the UR and adjacent body wall mesenchyme^56^ and there is also the regression of the right umbilical vein; this asymmetric regression has been proposed to make the right side weaker and be the reason why GS occurs predominantly on the right side.

While *Bmp1* and *Aclp* mutants provide insight into mechanisms of GS in the mouse, they do not phenotypically model *BMP1* and *AEPB1* (*ACLP*) autosomal recessive mutations in humans, which cause Ehlers‐Danlos syndrome, classic‐like 2 and osteogenesis imperfecta, type XIII, respectively. Neither syndrome is characterized by GS, although minor ventral wall anomalies (umbilical hernia, ventral hernia) have been reported in some patients with classic‐like 2 Ehlers‐Danlos syndrome.^318^, ^319^

### 6.4 Exomphalos

Exomphalos can be caused by defects at a number of stages of body wall closure e.g. failure in lateral body folding (Pitx2, Six5/6), delayed or stalled enclosure of the SBW (Tgfβ), failure of the umbilicus to close at E16.5 (Rock1/2). It is also possible that exomphalos results from a failure of gut rotation back into the abdominal cavity which physically obstructs VBW closure. Mutation in many genes have been shown to result in exomphalos (Table 2), a subset of which have been linked to exomphalos in humans (Table 3). The following discussion will focus on this latter group and highlight insights gained from these mouse models albeit with potential caveats.

Pitx2, is of interest, as it is a direct transcriptional target of canonical Wnt signaling and because heterozygous loss of function PITX2 mutations in humans results in Riegers‐Axenfeld syndrome which can include VBW anomalies ranging from exomphalos to excessive umbilical skin.^228^, ^267^, ^320^ While the *Pitx2* ^*−/−*^ mouse model can be used to understand mechanisms of PITX2 function in humans, there are, however, key differences. First, in humans the VBW anomaly is typically due to heterozygous loss of function mutations in *PITX2* whereas heterozygous *Pitx2* ^+/−^ mouse mutants are either normal or are characterized by a thinning of the VBW.^81^, ^253^ Thus, the homozygous *Pitx2* ^*−/−*^ mouse mutants which have a more extensive VBW closure phenotype encompassing both the thoracic and abdominal cavities, must be used for analysis.^80^, ^81^, ^85^, ^253^ Finally, in a subset of *Pitx2* ^*−/−*^ mouse mutants, body rotation is arrested which may contribute to the phenotype.^81^, ^253^ As the first step of embryo turning differs between mice and humans, mouse mutants with turning defects will not completely model the early steps of VBW development in humans. However, despite these differences, the *Pitx2* ^*−/−*^ mouse model can give insight into the mechanisms of VBW closure at later stages of development and how, the defect arises. Analysis of *Pitx2* ^*−/−*^ mutants has shown that there are defects in both the PBW and SBW (see Section 4.1; Tables 2 and S1). Reflecting the changes in the LPM and an intrinsic role of *Pitx2* in the hypaxial muscles, the musculature within the SBW is hypoplastic and disorganized.^82^ A direct transcriptional target of Pitx2 in fetal musculature is procollagen lysyl hydroxylase 1 (Plod1) which may contribute to the phenotype.^321^ Homozygous loss of PLOD1 in humans results in Ehlers‐Danlos syndrome Kyphoscoliosis type IV, which has some overlapping features with Riegers‐Axenfeld syndrome, including umbilical hernia.

### 6.5 Bladder/CE

In animal models, bladder and CE is associated with decreased development of the infraumbilical mesenchyme (reviewed by Ludwig et al.^322^). In both disorders, the umbilicus is positioned more inferiorly. Additionally, the external genitalia, which arise from the LPM,^247^ are often bifid. The bladder/CE may be due to insufficient mesenchyme to meet at the midline during the early lateral and caudal folding to form the PBW. Alternatively, the exstrophy may be due to insufficient merging of the mesenchyme along the midline at later stages of development due to decreased migration of mesenchymal cells from the umbilical area to the infraumbilical mesenchyme (see Section 3.7). Either deficit would render the VBW more prone to rupture or a failure to close.

Genetic profiling has identified many causative candidate genes (reviewed by^323^, ^324^). The strongest candidate is ∆NTAF63, an anti‐apoptotic factor, and one of the p63 isoforms. Decreased levels of ∆NTAF63 or changes in the p63 isoform ratios have been linked to bladder exstrophy in humans.^325^, ^326^ This bladder exstrophy phenotype is recapitulated in *p63* mouse mutants and analysis has focused on the alterations in bladder development.^78^ The midline defect is apparent by E11.5 indicative of an early change in PBW formation. ∆NTAF63 is expressed within the ventral bladder epithelium and the mouse mutants are characterized by increased cell death and decreased proliferation within the bladder epithelium. Additionally, the surrounding splanchnic mesoderm is hypoplastic which is linked to decreased expression of the *Fgf8* growth factor and *Msx1* transcription factor. Whether these alterations in bladder development are causative for the bladder exstrophy, is unclear. The bladder is an endodermal organ surrounded by splanchnic mesoderm and would not necessarily impact on the VBW, comprised of somatic lateral plate and paraxial mesoderm. P63 and its isoforms are also expressed in the epidermis where they control epithelial differentiation and epithelial‐mesenchymal interactions (reviewed by Li et al.^327^). It is therefore possible that hypoplasia of the VBW is responsible for the bladder exstrophy in *p63* mouse mutants, or concurrent ∆NTAF63 function in both the bladder and VBW ectoderm is essential for VBW closure. Conditional knockout of ∆NTAF63 or p63 within different cell populations in mice will be required to resolve this question.

A putative model of bladder exstrophy can also be observed in the *Alx4* mouse mutant.^50^, ^51^ The infraumbilical ventral mesenchyme fails to develop resulting in hypoplastic lower VBW, anterior bladder wall and hypoplasia in the dorsal GT. In addition, this mutant demonstrates a SBW closure defect restricted to the abdominal region similar to that seen in an exomphalos major anomaly.^50^, ^51^ However, this model lacks the characteristic exteriorization (schisis) of the bladder or the associated epispadias anomaly, and the urethra shows complete anatomical development at E18.5. Nevertheless, mutations in the ALX4 gene have been identified as a potentially causative factor in human bladder exstrophy‐epispadias complex.^210^ One suggested mechanism, leading to this constellation of anomalies in the lower ventral midline, is ectopic Shh signaling within the caudal VBW. A dose‐dependent increase in Shh signaling in the dorsal GT in the *Alx4* ^*Lst/+*^; *Gli3* ^*Xt/Xt*^; and *Alx4* ^*Lst/Lst*^; *Gli3* ^*Xt/Xt*^ double mutants also dictated the severity of dorsal GT hypoplasia. Nevertheless, the urethra demonstrated full anatomical development, excluding an epispadias component. These models similarly had the hypoplastic infraumbilical VBW, anterior bladder wall and the exomphalos type of anomaly that is seen in the *Alx4* ^*Lst/Lst*^ mutant.^50^, ^51^

### 6.6 Prune belly syndrome (PBS)

PBS is characterized in newborns/infants, predominantly male, by a thin and transparent ventral abdominal skin which has a “dried plum” appearance.^328^ Hypoplasia of the abdominal straited musculature and of the smooth muscles around the bladder together with kidney anomalies are also common in PBS.^329^ As these structures are derived from mesoderm^29^ (reviewed in Reference 330), one hypothesis regarding its etiology highlights a defect of mesenchymal/mesodermal development.^329^ Gene associations with PBS include mutation of the mesenchymal regulator HNF1β^331^ as well as copy number variations in Bmp signaling components^332^ known to drive muscle differentiation^333^ and kidney morphogenesis^334^ as well as urogenital development: urogenital defects are also a common feature of PBS.^329^ Males with PBS can harbor mutations in X‐linked FLNA, an actin binding protein that also engages with extracellular matrix receptors.^211^ Mouse mutants for these candidate genes in humans, however, are either early embryonic lethal (*Hnf1β* ^145^) or exhibit TAS (reduced dose of Bmps^93^) or exomphalos (*Bmp* ^93^ and *flna* ^145^). *Bmp* mutants and the *Flna* mouse mutant *Dilp2* do, however, exhibit defects in mesodermal and mesenchymal growth^93^, ^145^ which may enable mechanistic insight into PBS. It will also be important to understand whether disruption to mesenchymal/mesodermal development leads to the type of ventral skin defects characteristic of PBS.

## 7 FUTURE DIRECTIONS

An understanding of how the VBW develops gives insight into mechanisms of VBW anomalies which can arise due to failure in either PBW or SBW development. TAS, where all the thoracic and abdominal organs are exposed to the amnion, is the result of the failure of the PBW to form (e.g., Gata4, Furin) or the failure of invasion of SBW into the PBW (Tfap2α). Ectopia Cordis can arise due to a failure in the cranial fold or a defect in thoracic SBW closure while bladder and CE can be due to a failure in the caudal fold or in the anterior–posterior growth of lower abdominal VBW. Finally, exomphalos is due to an anomaly in the abdominal PBW or SBW or closure of the UR. While numerous mouse models have been generated that give molecular and cellular insight into mechanisms of VBW development, there are still significant gaps in our understanding. Particular challenges include that the genetic mouse models do not always recapitulate anomalies that occur in humans due to the same gene mutation. A fully representative model for GS in humans is still unavailable. In humans, VBW anomalies are often multifactorial making it difficult to pinpoint the genetic and environmental causes and although not yet clinically and genetically demonstrated may also arise by somatic mutations that result in mosaicism where the VBW consists of some cells carrying a gene mutation. As the VBW arises by large‐scale tissue movements which involve collective cell movements, mosaic inactivation of gene function may be particularly disruptive. Mosaic disruption in PCP genes that control large‐scale tissue behaviors can be particularly impactful as demonstrated in the early thoracic wall and also, another tissue, the closing neural tube.^8^, ^335^

Despite these reservations, an understanding of embryonic development will shed insight into mechanisms of VBW development and how anomalies may arise. Current gaps in our knowledge include:

1. The interplay between different cell populations during morphogenesis and cell differentiation within the VBW needs to be resolved. This includes an understanding of the role of Tgf‐β signaling within the myofibroblasts which are proposed to be pioneering cells during VBW closure and of the PBW in promoting growth of the SBW. The potential role of coelomic cells, and physical contribution to VBW, also needs to be resolved.
2. A further understanding of cell movements, including large‐scale tissue movements, during VBW closure is required. Growth of the body wall is predominantly ventral ward. However, studies in animal models have indicated considerable cell movements during VBW closure that contribute to growth along the other axes. These include convergent‐extension movements at the leading edge of the thoracic body wall, extensive anterior–posterior movements of the thoracic LPM and mesenchymal migration inferiorly into the infraumbilical region.
3. Fate mapping studies indicate that the ectoderm and mesoderm move together in the thoracic SBW. The fate of the PBW mesoderm and ectoderm needs to be determined. Does the PBW undergo apoptosis or migration to contribute to other regions of the VBW.
4. Merging of epithelia: The epithelial behaviors during folding and merging in PBW development need to be analyzed. Do these merging processes create “weak points” that are vulnerable to rupture?
5. Analysis of p53/ROS activity/production, respectively, would identify cell populations that are particularly vulnerable to teratogens. If p53 or ROS activity is endogenously higher in a particular cell population, these cells would be expected to be more susceptible to teratogens which would increase p53/ROS activity above a particular threshold driving apoptosis.

## Supporting information


**TABLE S1:** Details of mouse mutants and VBW phenotypes.
