# Supplementary material for: Ventral body wall closure: Mechanistic insights from mouse models and translation to human pathology
Source: Dev Dyn. 2024 Sep 25;254(2):102–41. doi: 10.1002/dvdy.735 (PMC11809137; doi:10.1002/dvdy.735)
Supplement: Supplementary file 1 — TABLE S1: Details of mouse mutants and VBW phenotypes. [file DVDY-254-102-s001.docx]

**Table S1: Details of mouse mutants and VBW phenotypes**

| **Mouse mutant information** | **Details of VBW phenotypes for each mouse mutant** |
| --- | --- |
| **Transcription Factors/DNA binding factors** |  |
| ***Alx4***  *Alx4tm1qw* null  *Alx4****^Lst/Lst^*** (Strong Luxoid mouse) – 16bp deletion in exon 1 encoding DNA binding domain: potential frameshift | • *Alx4tm1qw* (129/Sv background) exhibits **98%-100% penetrant** exomphalos. Failure of abdominal but not thoracic SBW enclosure, thin PBW structure retained. Phenotype apparent by E15.5. Decreased muscle and dermal development but no change in *Pax3* or *MyoD* expression at E10.5 SBW leading (medial) edge indicating early muscle development is normal (Qu et al. 1997).  • *Alx4****^Lst/ Lst^***: Matsumaru et al (2014) report on pelvic girdle and ano-rectal malformations, lack of growth of lower abdomen, genital hypoplasia. Decreased migration of mesenchymal cells from infra-umbilical region leading to reduced separation of the umbilical ring and genital tubercle, n=5/5 *Alx4****^Lst/ Lst^*** embryos. Authors report decreased expression of fibronectin, ***Pitx1****,* ***AP2α, Mab21l2*** in umbilical ring and dorsal genital mesenchyme. |
| ***Alx1(Cart1)/Alx4***  *Alx1^-/-^* neo insertion ∆ exon 1-3  *Alx4tm1qw* | • Only double homozygotes exhibit split sternum (Qu et al, 1999). *Alx1* is expressed in lateral mesoderm in the rat (Zhao et al, 1994). |
| ***AP2α* (Tfap2α)**  Neo insert deletes exons 5-7  LacZ knock-in into exon 7  *AP2α* ^flox/flox^ : *Foxg1-cre*  AP2α ^hygro/hygro^/ WT chimaeras  *AP2α* ^flox/flox^ : *keratin14-cre*  *AP2α* ^flox/flox :^*Crect-cre* | Primary body wall is thinner by E11.5, ruptures by E13.5 and is lost by E15.5. Thereafter, ventral organs lack a surface covering. (Schorle et al, 1996; Zhang et al, 1996, Brewer and Williams, 2004, Nottoli et al, 1998). In addition, there is reduced growth of lower abdomen and genital tubercules fail to fuse (Brewer and Williams, 2004). AP2α is also expressed in the ectoderm/epidermis of the SBW (Leask, Byrne and Fuchs, 1991).  • *AP2α*^neo/neo^ – null exhibits thoracoabdominoschisis (Schorle et al, 1996; Zhang et al, 1996).  • *AP2α* ^LaczKI/LacZKI^ – primary body wall defects reported, sternal bands and abdominal musculature are absent. Skin fails to move ventral-ward. LacZ expression observed at leading edge of ventral SBW. Other defects included absence of growth of lower abdomen and failure of genital tubercule fusion. Abnormally large umbilical ring at E15.5 linked to increased cell death in this area and across ventral SBW, proposed to reduce deposition and growth of infra-umbilical mesoderm implicated in lower abdomen and genital tubercule development (Brewer and Williams, 2004).  • *AP2α^flox/flox^* *Foxg1-Cre* (incomplete ectodermal Cre expression?) E15.5 embryos exhibit enlarged umbilical ring with variable levels of extrusion of internal organs, defective fusion of genital tubercules but thin ventral membrane covering is intact. At E18.5 epidermis formation is apparent and slit-like openings remain in thorax and abdomen (Brewer and Williams, 2004).  • *AP2α* ^hygro/hygro^ / WT chimaeras, 67% of those with mutant phenotypes exhibited body closure defects. Some chimaeras exhibited fully open ventral body wall whereas others exhibited abdominal wall defects only (Nottoli et al, 1998).  • *AP2α* ^flox/flox :^*keratin14-cre* epidermal specific knock-out, no body wall defect (Wang et al, 2006).  • *AP2α* ^flox/flox :^ *Crect-cre* early ectoderm-specific (from E8.5) does exhibit a ventral body wall defect, predicted transcription factor profiles of wild-type versus mutant reveals reduced Wnt signalling from craniofacial ectoderm, also reduced Krt5/14 but increased Krt8/18 expression suggesting defect in ectodermal differentiation (Van Otterloo et al, 2022). |
| ***Ataxin1/Ataxin-1-like***  *Atxn1-/-/Atxn1L-/-* | • 45% penetrance in *Atxn1^-/-^/Atxn1L^-/-^* double homozygote embryos, 17% in *Atxn1^+/-^/Atxn1L^-/-^* ,8% in *Atxn1^-/-^/Atxn1L^-/+^* and 4% in *Atxn1L^-/-^* (E18.5) (Lee et al, 2011). Atxn1 and Atxn1L interact with and stabilise Capicua (Cic), a DNA binding protein. Studies performed in lung suggest that an *Atxn1* and *Cic* interaction is important for ECM remodelling and authors propose a similar function for these genes in the VBW (Lee et al, 2011). |
| ***Barx1***  *Barx1^Int^* mouse | • Forced *Barx1* expression in intestinal mesenchyme/gut sub-mucosa from E9.5 via *Bapx1*-cre, *Barx1* reported as 100X higher in intestinal mucosa in mutant compared to WT.  • Intestine has not returned to the body cavity by E17.5, **100% penetrance**. Lack of regionalised intestinal tissue, instead intestine exhibits gastric-type throughout. Thinner smooth muscle layers and disruption of intestinal asymmetry in dorsal mesentery linked to failure of mid-gut rotation (Jayewickreme and Shivdasani, 2015). |
| ***Capicua (cic)***  *Cic^∆2-6^* gene knockout | • *Cic^∆2-6^* allele lacks DNA Binding and Repressor domains. 70% penetrance of exomphalos at E18.5 (Simón-Carrasco et al. 2017). Authors report disruption to *Pea3* family of transcription factors in *Cic^∆2-6^* / *Cic^∆2-6^* fibroblasts. |
| ***Fendrr***  *Fendrr*^3xpA/3xpA^ : exon 2 replaced with 3xpA stop cassette | Long non-coding RNA exhibiting restricted expression to caudal LPM. Open ventral body wall, embryonic lethal at E13.5 (Grote et al, 2013). Proposed mechanism: Epigenetic disturbances lead to aberrant LPM differentiation and reduced thickness of the VBW (Grote et al, 2013). |
| ***Gli3***  *Gli3^Xt/Xt^ extra-toes* mutant  intragenic deletion of *Gli3* | • *Gli3^Xt/Xt^* mutant: split sternum (Johnson, 1967; Mo et al. 1997; Hoelzl et al. 2017), exomphalos (Matsumaru et al, 2011), penetrance not reported. See also SHH signalling below for genetic interactions with other key players. |
| ***AHDC1 (Gibbin)***  mosaic CRISPR-generated gene knockout | • Exomphalos (reported at E18) in group of mosaic knock-out embryos with most severe phenotype. Immature dermis was associated with disruption to skin differentiation and skin attachment to dermis. In hES CRISPR knock down cells, ***Pitx2, GATA4, Hand2, Prx1*** and ***Pdgfrα*** expression is disrupted. Gibbin interactome also linked to **BMP** and **Retinoic Acid** signalling**.** Gibbin proposed to function alongside GATA3 to modify chromatin and therefore impact transcriptional profiles within tissues (Collier et al, 2022). |
| ***Glucocorticoid receptor***  *GR*^flox/flox^ with Dermo-1 cre  *GR*^neo/neo^ ∆ exon 2 | • *Dermo-1 cre* generates mesenchymal specific knock-out on C57BL6 background. Histology at E16.5 indicates normal differentiation of SBW and suggests stalling of SBW closure (Li et al, 2013). Genetic background variations involved as an independent mouse Glucocorticoid receptor knock-out, GR^neo/neo^ on C57BL6/129J background- no exomphalos (Cole et al, 1995). |
| ***Grainyhead-like 2 (Grhl2)***  *Grhl2^Nisw^, nonsense mutation, ENU chemically induced* | On a 129S1/SvImJ background, there is a high percentage of embryonic lethality from E9.5 and only 5% of *Grhl2^Nisw/Nisw^* embryos survive beyond this stage. Some embryos exhibit a more severe phenotype where embryo turning has failed. On a C3H background 50% of *Grhl2^Nisw/Nisw^* embryos exhibit a VBW phenotype. Double heterozygotes between **AP2α^-/+^ a**nd *Grhl2^Nisw^* did not generate a VBW defect despite their both exhibiting the TAS phenotype (Pyrgaki & Niswander, 2011).  *Grhl2^-/-^* embryos on a C57BL6 background do not exhibit VBW defects which is also the case for *Grhl2^-/+^ Grhl3^-/+^* double heterozygote embryos (Rifat et al, 2010).  Studies in the neural tube suggest a role for *Grhl2* in the modulation of epithelial cell shape, cell junctions and tissue mechanics |
| ***Hand1***  *Hand1^β-gal/fx^-* βgal-neo insertion into 5’ region.  *Tlx2*-Cre deleter | Herniating gut to the right of the umbilical cord suggests gastroschisis. *Hand1*^-/-^ is embryonic lethal at E9.5.  • Deletion of *Hand1* in LPM using *Tlx2*-cre deleter which was generated using 3Kb regulatory region of *Tlx2* reported to not drive gene expression in extraembryonic tissues (Maska et al, 2010). |
| ***Hic1*, hypermethylated in cancer 1**  *Hic1*^neo/neo^ - entire coding sequence deleted  *Hic1^flox^*^neo/floxneo^  *Hic1*-citrine reporter | • 50% of *Hic1*^neo/neo^ embryos exhibited exomphalos at E18.5 (Carter et al, 2000). *Hic1*-citrine mouse confirms expression in somites and LPM (Pospichalova et al, 2011). Using single cell-omics and fate mapping Arostegui et al. (2022) reveal that Hic1-expressing cells generate a distinct sub-set of mesenchymal cells in the limb which contribute to multiple limb lineages. |
| ***Hoxb2 and Hoxb4***  Generation of knockout mice involved insertion of PolII Neo cassette into *Hoxb4* or *Hoxb2* loci which disrupts neighbouring *Hoxb* gene expression. | • *Hoxb4* PolII Neo insertion: failure of SBW migration in thorax leads to 37% lethality with 100% split sternum. Thinner thoracic PBW and loss of ***Alx3*** and ***Alx4*** expression in thoracic PBW at E11 but not at E10.5. Shape of sternum along D-V and M-L axes normal indicative of correct convergence and extension movements. Cell survival unchanged at E12.5 and E13.5. Thoracic SBW mesoderm in newborn pups is less differentiated than WT. Anteriorly displaced and enlarged umbilical ring with shortening of the anterior-posterior length of the abdominal wall: structural cardiac defects also reported suggesting Pentalogy of Cantrell-like (Manley et al, 2001)  • *Hoxb2* PolII Neo insertion: similar phenotypes to *Hoxb4* but less severe and more variable (Barrow and Capecchi, 1996; Manley et al, 2001). Neo cassette insertion into *Hoxb2* and *Hoxb4* gene loci also disrupts neighbouring *Hoxb* gene expression (Barrow and Capecchi, 1996; Manley et al, 2001; Ramirez-Solis et al, 1993). |
| ***Msx1 & Msx2***  Double homozygote mutant  *Msx1* ^neo/neo^- ∆ homeobox  *Msx2*^neo/neo^- insertion exon 2 | *Msx1* and *Msx2* expression is regulated by **BMP** signalling in other tissues. Defect apparent by E12.5. Disorganisation of muscles and connective tissue in abdominal SBW of double homozygote mutant (Ogi et al, 2005). |
| ***p63***  2 null alleles: integration of pTV12E(60) vector with a gap-repair mechanism process which generated two mutations independently in exon 6 and 10 which truncate protein prior to DNA binding region and conserved C-terminus respectively | • Both null alleles generate same phenotype.  • Null alleles on 129/C57BL6 background exhibit limb defects but not VBW defects indicating genetic background effects (Mills et al, 1999).  • *p63*^-/-^ -C57BL6 background, 33% penetrance of bladder exstrophy, an umbilical hernia (with and without membrane cover) and bifid genitalia (latter evident at E11.5), remaining littermates exhibit a thin bladder wall and dilated bladders (Cheng et al, 2006). Bladder wall ectoderm does not develop into a stratified urothelium and remains as a monolayer of squamous epithelium retaining K18 expression (early ectoderm keratin marker). Apoptosis is elevated in ventral PBW with increases in prop-apoptotic mediators e.g. *Bax*. Failure of mesenchymal growth in urogenital sinus and loss of bladder smooth muscle (Cheng et al, 2006). Ching et al (2010) identify bladder expression of anti-apoptotic p63 RNA isoforms and dysregulation of their expression in 11/15 patients with bladder exstrophy but do not identify any p63 mutations in the same patients |
| ***Pitx2***  *Pitx2*^flox/flox^ and *Pitx2*^neo/neo^  *Pitx2*^LacZ/LacZ^ - insertion of LACZ disrupts either exon 4 or exon 3 and 4  *Pitx2*^hd-/-^ - deletion of exon 5 and part of exon 6 including homeobox | • *Pitx2*^flox/flox^ exhibits turning defects and a completely open ventral body wall due to failure of PBW formation (Gage et al, 1999).  • Pitx2^neo/neo^ - hypomorphic mouse, does not exhibit VBW defects (Gage et al, 1999)  • *Pitx2*^LacZ/LacZ^ : disruption of exon 4, embryos exhibit failure of ventral closure at E10.5 associated with a failure of the left lateral body wall to tun inwards, authors also describe extrusion of visceral organs to the left side and an anti-clockwise bending of the body axis at a site close to the thoracic/abdominal junction at E13.5, survival to E14.5. No changes in expression of **BMP**s and Prx1 (Kitamura et al, 1999).  • *Pitx2*^LacZ/LacZ^ exhibits defects in abdominal somite formation and abnormal muscle development in SBW, shows loss of **Hox** gene expression in abdominal body wall (Eng et al, 2012, Shih et al, 2007).  • *Pitx2*^LacZ/LacZ^ – also see Lin et al, 1999 where LacZ insertion disrupts exon 3 and exon 4.  • *Pitx2*^hd-/-^ - Lu et al. 1999 report failure or delay in caudal embryonic turning. Embryos that had undergone turning exhibit exomphalos.  • *Pitx2*^LacZ/LacZ^ (129/Sv, Lin et al, 1999): decreased proliferation in VBW (E10.5; Kioussi et al, 2002)  • Hilton et al, 2010 report that Pitx2 regulates Tbx family members in abdominal body wall. Other Pitx2 target genes have been identified by Eng et al, 2010. Kioussi et al, 2002 report *Pitx2* transcriptional regulation by canonical Wnt/Dvl signalling in musculature, eye and heart. VBW was not analysed. |
| ***Runx1 & Runx2***  *Runx1*^flox/flox^ ∆ exon 4 DNA binding  *Runx2^-/-^ LacZ/neo* insertion into Q/A repeat containing exon | • *Runx1*^flox/flox :^ *Prx1*-cre: 100% delay in sternal development in 2 weeks old pups (Kimura et al. 2010).  Runx1 and 2 control thoracic body wall closure by unknown mechanisms and also sternal differentiation.  • *Runx1*^flox/flox :^ *Prx1*-cre: *Runx2^-/-^* double homozygote newborn pups: **100% penetrance** absence of sternum and open thoracic wall with organ protrusion i.e. ectopia cordis. *Runx2^-/-^* newborn pups: normal sternum development. Loss of *Runx1/2* in LPM before E13.5, but not after, results in VBW closure defect. (Kimura et al. 2010). *Sox5* and *Sox6* expression, which are downstream of *Sox9*, is reduced in the thoracic mesenchymal condensations of the double homozygote as well as a*1(II) collagen* expression*.* Cell survival unaffected in mutants at E14.5 (Kimura et al. 2010). |
| ***Six4 & Six5***  Double homozygote mutant  Six4-/- : ∆homeobox/SixHR  Six5-/- insertion LacZ/Neo into exon 1 | • Few *Six4-/+:Six5-/-* E18.5 embryos exhibited exomphalos compared to 31% of *Six4-/-:Six5+/-* and **100%** of double homozygotes. MicroCT reveals failure of somatopleure bending by E11.5. Decreased cell proliferation and survival within PBW at E10.5 (Takahashi et al. 2018).  • E13 umbilical ring was larger and positioned more anteriorly in double homozygotes. Ventral midline tissue (PBW) above umbilicus was thinner than WT but thicker more laterally both above umbilicus and at level of umbilicus suggesting defect in cell migration during morphogenesis of PBW. Normal differentiation of abdominal musculature (Takahashi et al. 2018).  • Impaired differentiation of coelomic epithelium into mesothelial progenitors. Apical **PODXL** distribution was disturbed in E11.5-E12.5 double homozygote coelomic precursors whilst basal laminin expression became fragmented (Takahashi et al. 2018). |
| ***Sox C subfamily***  Sox11-/- : ∆all exons LacZ knock-in.  Sox4^+/flox^/Sox11^+/flox^  Sox4^+/flox^/Sox11^+/flox^ / Sox12^+/flox^  Sox4^+/flox^/Sox11^+/flox^  Prx1-cre (LPM) | *Sox11* also expressed in the epidermis (E13.5, Miao et al, 2019)  • 35% *Sox11^LacZKI^/^LacZKI^* embryos exhibit exomphalos (Sock et al, 2004). 50% of *Sox11^-/-^* and *Sox4^+/-^/Sox11^+/-^* exhibit exomphalos, *Sox4^+/-^/Sox11^-/-^* exhibit turning defects and arrested development at E8.5. (Bhattaram et al. 2010).  •*Sox4^+/-^/Sox11^+/-^ Prx1*-cre was shown to exhibit reduced cell survival in limb mesenchyme and somites, cell proliferation was unaffected (Bhattaram et al, 2010). In other organs, cell survival and cell proliferation function is mediated via Tead2, a direct transcriptional target and co-factor (Tsang et al. 2020; Angelozzi et al. 2022). *Prx1*-Cre inactivation of *Sox4, 11* and *12* with homozygous deletion of at least 2 of these genes – leads to open thoracic body wall (Angelozzi et al. 2022). Transcriptional targets in other tissues include **Wnt** and **TGFβ** pathway components and myofibroblast components (Tsang et al. 2020; Angelozzi et al. 2022). |
| ***Zic3***  Spontaneous Bent-tail mouse  Deletion of entire *Zic3* coding domain plus other genes  *Zic3*^-/-^ : replacement of coding region with LacZ | • Only 3.5% of embryos exhibit an open ventral body at E15.5 (Klootwijk et al, 2000).  • *Zic3* null does not have a VBW defect (Purandare et al. 2002). |
| **Extracellular signalling ligands & Receptors** |  |
| **BMP signalling**  ALK3- BMPR1A type 1 (receptor)  *Alk3*^flox/flox^ and Dermo-1-cre mesoderm -specific  BMP2 (ligand)  *Bmp2^fn^* : flox/flox plus neo insertion into 3’UTR  *Bmp2*^HPRT/TK^ insertion ∆exon 2 -null allele  BMP4 (ligand)  *Bmp4*^LacZ/+^  *Bmp4^S2G/S2G^* point mutation in propeptide | • *Alk3*^flox/flox^ dermo1-cre (mesodermal knock-out), E13.5 embryos exhibit exomphalos (Sun et al, 2007).  • *Bmp2* ^null/null^ exhibited turning defects at E8.5. *Bmp2^fn^*/*Bmp2*^HPRT/TK^ (*Bmp2^fn^* assumed hypomorphic with lowered BMP levels) exhibited a ventral closure defect at low frequency but which was most penetrant (14%) when mother was *Bmp2*^HPRT/TK^/+ authors suggest some maternal influence on body wall formation (Singh et al, 2008).  • *Bmp2* ^null/+^ adults exhibit split sternum (67% penetrance) (Goldman, Donley and Christian, 2009).  • *Bmp4*^Lacz/S2G^ surviving pups exhibit split sternum (n=3/3 newborns) whilst embryos exhibit TAS (50% of embryos) (Goldman et al, 2006).  • *Bmp2* ^null/+^ with *Bmp4*^Lacz/+^ n=55% embryos exhibit exomphalos and 1 embryo of n=9 exhibit TAS (Goldman, Donley and Christian, 2009).  • Chick embryos with over-expression of the BMP inhibitor noggin also exhibit exomphalos (Nerurkar et al, 2017). This may be to do to with alterations in the looping and morphogenesis of the midgut and/or an intrinsic effect within VBW. |
| ***Tgfβ* signalling**  *Tgfβ2* (ligand) neo insertion exon 6  *Tgfβ3* (ligand) neo insertion exon 6  *Tgrβr2* (receptor) neo insertion into exon 4  *Transgelin-*1 cre specific to leading edge myofibroblasts  *Prx1*-cre specfic to LPM  *Alk5^flox/flox^* (receptor) | • E14.5 *Tgfβ2/Tgfβ3* double null embryos lack sternum, exhibit complete failure of ventral closure and do not survive beyond E15.5, **penetrance 100%**. Thorax and abdomen are both open and missing distal (ventral) part of the ribs. A thin ectoderm is found on ventral side covering the internal organs but lacks LPM, significantly reduced ventral-ward movement of myotome and skin. In dorsal body wall where skin was generated, connective tissue was defective (Dunker and Krieglstein, 2002).  • *Tgfβr2* null/*Transgelin-1* cre receptor signalling is lost in migratory VBW myofibroblasts (Aldeiri et al, 2017).  • *Alk5^flox/flox^-Dermo-1* cre: deletion in ventral mesenchyme (somitic and LPM): completely open ventral body wall covered with a thin membrane, shortened body axis, penetrance not reported but anomaly apparent from E12.5, sternum split (Matsunobu et al, 2009).  • *Tgfβr2-Prx1-*Cre knockout (*Tgfβr2^f/f^;Prx1Cre* and *Tgfβr2f/-;Prx1Cre*) – lacks sternum (LPM derived), ribs present (somite derived) but do not “meet” at midline indicating thoracic body wall SBW is “open”. *Prx1*-Cre deletes in LPM. Histology to analyse thickness of VBW not shown (Spagnoli et al. 2007).  • *Tgfβr2^f/f^;Prx1*Cre – sternum split, severe SBW closure defect in thoracic VBW (Seo & Serra, 2007). |
| ***EphrinB* and *EphB* signalling**  *EphrinB1* deficient *∆* exon 2 (ligand)  *EphrinB1^flox^* -Meox2-cre mesenchyme specific  *EphrinB2*^LacZ/LacZ^ ∆cyto tail LacZ insertion  *EphB2^-/-^  ∆amino acids 29-50*  *EphB2* ^LacZ/LacZ^ ∆cyto tail LacZ insertion*.*  *EphB3^-/-^ ∆* kinase domain | • *EphrinB1* null males (hemizygous null) exhibit delayed sternal fusion at E12.5 (Compagni et al, 2003)  • Delay in SBW enclosure observed in E14.5 *EphrinB1* deficient embryos (hemizygous null males and heterozygous females as *EphrinB1* is X-linked), **100%** penetrance in *EphrinB1^-/+^* females (Compagni et al, 2003). Authors proposed mosaic loss of function EphrinB-EphB signalling in *EphrinB* heterozygote mice. *Prx1*-Cre deletion of *EphrinB1* does not result in VBW anomalies indicating non-LPM derived origin of key EphrinB-expressing cells (Compagni et al, 2003). Furthermore, Davy et al (2004) stated that 70% of *EphrinB1^flox^* -*Meox2-*cre embryos exhibited body wall closure defect indicating a signalling role from somite derived mesoderm, embryonic stage was not reported.  • Only 40% of *EphB2^-/-:^ EphB3^-/-^* double homozygotes exhibit SBW defect (Orioli et al, 1996, Dravis and Henkemeyer, 2011) in 129 but not CD1 background (Dravis and Henkemeyer, 2011).  • Ephrin/Eph receptors signal bi-directionally. At ventral midline of body wall Eph receptors are proposed to predominantly act as ligands for Ephrin reverse signalling (Davy et al, 2004; Dravis and Henkemeyer, 2011). *EphrinB2*^LacZ/LacZ^ abolishes reverse signalling leaving forward signalling intact whilst *EphB2* ^LacZ/LacZ^ abolishes forward signalling leaving reverse signalling intact. |
| **FGF signalling:**  *Fgf8/9/17/18* (ligands)  *Fgf8* ^flox^ & *Fgf18* ^flox^  *Fgf8*^∆^ - null allele (∆ exon2,3)  *Fgf9-* null allele *Tm1 LACZ* knock-in  *Fgf18*^∆^ - null allele (∆ exon1,2)  *Fgf17^∆/∆^ -*null, insertion of neo: ∆ 5’UTR-exon 2  *Fgfr1&2* (receptors)  *Fgfr1^flox/flox^ & Fgfr2^flox/flox^*  Double *Fgfr1&2* knock-out: Rosa EsR cre induced at E8.5 | • 39% of *FgfR1/2* conditional knock-out (E8.5 onwards) exhibited exomphalos: delayed epidermal development in knock-out embryos which do not exhibit this phenotype. Embryos with exomphalos exhibit a thickened dermis with dense underlying CT and additional CT layers, also disrupted ventral translocation of the Pannus Canniculus muscle. Knock-out of *Fgfr1* or *Fgfr2* alone did not lead to exomphalos (Nichol et al, 2011).  • Triple *Fgf 8/17/18* mutants (using *Fgf8* ^flox^ & *Fgf18* ^flox^ which were conditionally inactivated in mesoderm arising from primitive streak of gastrulating embryos via *Tg(T-cre)1Lwd-*cre, with *Fgf8*^∆^ , *Fgf18*^∆^ and *Fgf17^∆/∆ :^* Tcre: *Fgf8*^flox/∆^ :*Fgf17*^∆/∆^: *Fgf18*^flox/∆^) exhibit exomphalos and reduced growth of lower abdomen (71% penetrance) at E18.5, VBW defect is apparent from E12.5 in 9% of embryos and at E13.5 in 76% of embryos (Boylan et al, 2020). TCre; *Fgf8*^flox/∆^:*Fgf17*^∆/∆^: *Fgf18*^flox/+^ and TCre; *Fgf8*^flox/+^ :*Fgf17*^∆/∆^: *Fgf18*^flox/∆^ embryos occasionally exhibited exomphalos: 15% and 52% respectively. Morphometric analysis reveals enlarged umbilical ring at E12.5-E13.5 as well as differences in the length of the PBW between the different mutant combinations (Boylan et al, 2020).  • *Cited-1* cre inactivates *Fgf8* in presomitic mesoderm (PSM) whilst *Meox-1* cre inactivates in the somite. *Cited-1* cre *Fgf8*^flox/∆^ :*Fgf17*^∆/∆^: *Fgf18*^flox/∆^ embryos exhibit 33% penetrance of exomphalos: authors propose FGF8 deficiency in the PSM associated with reduced rostro-caudal length of somites that contribute to thorax and abdomen which may impact on somitic mesoderm movements. Somitic inactivation of *Fgf* ligands had no effect suggesting no requirement in dermamyotome or myotome (Boylan et al, 2020). Overall *Fgf* mutants suggest effects of FGF signalling on PBW and umbilical ring morphogenesis.  *Fgf9* null exhibits small exomphalos, *Fgf9* is expressed in the mesothelium (Patzek et al, 2023). |
| ***Furin***  *Fur*^-/-^ : PGKhygro insertion into exon 4 | • Failure of embryo turning, the gut endoderm and the PBW fail to properly fuse at the ventral midline. Severe disorganization of all ventral tissues, phenotype is lethal by E10.5. (Roebroek et al, 1998). Chimeras of mutant and wildtype cells indicate furin is required in the mesoderm (Constam and Robertson, 2014). |
| ***IGFII* overexpression**  maternal deletion of IGFIIR and deletion of H19 locus [Igf2r(m−/+)/DH19(m−/+)] | *Igf2* locus and *H19* locus reside within imprinted area of mouse chromosome 7, linkage is conserved in humans. Mouse mutant ∆H19, where H19 and upstream sequence is deleted, exhibits increased transcription of *IGFII* leading to increased birth weight, named the H-phenotype. Mice lacking IGFIIR on maternal allele (IGFIIR^-/+^) exhibit reduced birth weight and other severe defects (named R-phenotype). Eggenschwiler et al (1997) used both these mutants to generate a double heterozygote mutant known as R/H. Lethality observed from E12.5 with no embryos surviving beyond E17.5 where embryos were 200% of normal weight, also organomegaly. |
| ***Pcsk5***  *Vcc* ENU mouse mutant  *Pcsk5*^-/-^ : deletion exon 1  *Pcsk5*^flox/flox^: *Meox2*-cre | Serine protease involved in processing of secreted proteins and TM proteins including **TGFβ** family members. Meox-2 conditional knockout in epiblast and somitic mesoderm recapitulated exomphalos phenotype of *Vcc* ENU mutant (RNA transcript generated but no protein) and *Pcsk5* null (Essalmani et al, 2008) but with reduced penetrance. Proposed mechanism: regulation of **TGFβ (**GDF-11) signalling (Szumska et al, 2008). |
| ***Pdgfr*α**  *Pdgfrα* (receptor)  *Patch (Ph/Ph)* mutant  *Pdgfrα* ^flox/flox^ with c*re/ERT*  *Pdgfrα^-/-^* neo insertion into signal peptide & Ig domains 1,2  *Pdgfa and pdgfc* (ligands)  *Pdgfc^-/-^ SA-IRES-βgeopA* insertion into exon 2 | • *Ph/Ph* mutants have a split sternum (Payne et al. 1997;) and exomphalos (Schatteman et al, 1992). *Ph/Ph* mutant exhibits thin epidermis with no dermis and lack of connective tissue.  • Soriano, 1997 reported failure to close sternal bands and exomphalos at E12.5 in *Pdgfrα^-/-^* embryos and increased apoptosis in the somites, particularly the dermomyotome, which is the progenitor of VBW musculature.  • Conditional deletion of *Pdgfr*α with cre/ERT activation from E9.5 and E10.5 resulted in thoracic and abdominal wall defects (27% of all E18.5 embryos). Deletion from E11.5 only affected abdominal wall closure where the VBW was thin with lack of VBW musculature (Qian et al, 2017). Increased apoptosis in somites and VBW was observed at E12.5 (Qian et al, 2017).  • *Pdgfa*/*pdgfc* double knockout: exomphalos or sternal defects not reported. E10.5 ES cell derived *Pdgfc^−/−^: Pdgfa^−/−^* embryos exhibited disrupted metameric organization of myotome (Ding et al, 2004). |
| **Increased SHH signalling**  Compound *Gli3^Xt/Xt^*: Alx4^Lst^ alleles  *R26-SmoM2:CAGG cre-ER*  Generates a constitutively active form of Smoothened | • *Gli3^Xt/Xt^* exomphalos phenotype is progressively exacerbated by additional alleles of *Alx4*^Lst^, penetrance not reported. Exomphalos and pubic diastasis phenotypes observed in *Gli3*^Xt/+;^ *Alx4*^Lst/Lst^ mutant were restored by removal of one allele of *Shh* i.e. *Gli3*^Xt/+;^ *Alx4*^Lst/Lst^; *Shh*^+/-^.  • Genital hypoplasia is mild in *Gli3^Xt/Xt^* mutant but severe in *Alx4*^Lst/Lst^:*Gli3^Xt/Xt^* double homozygotes (Matsumara et al, 2011 & 2014).  • Ectopic/increased induction of SHH signalling also achieved through R*26-SmoM2:CAGG cre-ER* mice. Exomphalos was observed but only when cre-ER induction occurred at E9.5, E10.5 or E11.5, pubic diastasis phenotype observed when cre-ER induction occurred at E10.5 (Matsumara et al, 2011). In *R26-SmoM2:CAGG cre-ER* mice embryos, there is increased cell death within the VBW, midline musculature also did not form, this could be a result of ectopic Shh signalling in VBW between E9.5 to E12.5? |
| **Wnt signalling**  *Wntless^flox/flox^*  *Dermo-1*-cre mesoderm specific (E11.5 onwards)  *Msx2*-cre ectoderm specific  *Porcn*^-/-^ : ∆ exon 2,3  *Lrp5 ^flox/flox^ and Lrp6 ^flox/flox^*  *Dermo*-1-cre mesoderm specific  *Gsk3*^-/-^: neo insertion ∆ ATP-binding loop | Wntless is a cargo protein which functions in Wnt ligand secretion and is expressed in mesenchyme and ectoderm tissue compartments (Zhu et al, 2012)  • In *Dermo-1-*cre conditional *Wntless* is lost from the LPM and somitic mesoderm, embryos exhibit ectopia cordis at E14 and failure of sternum fusion suggesting disruption of Wnt/β-catenin signalling at the ventral midline. There is an absence of cell death at the ventral midline where the sternum is forming, which occurs in wild-type, authors suggest this involves SMA-positive myofibroblast cells and that Wntless is important for cell death at the thoracic ventral midline between the sternal bars as they close. *Dermo-1-*cre *Wntless* is embryonic lethal at E14.5 (Snowball et al, 2015). *Dermo-1* cre is active from E11.5 onwards (Ohtola et al, 2008).  • *Msx2-cre: Wntless* is lost specifically from ventral body wall ectoderm from E12.5: associated with loss of *Lef1* and *Axin2* RNA expression which are effectors of canonical Wnt signalling. Exomphalos reported at E18.5: severe phenotypes show open body wall with a ventral cover of thin ectoderm and mesoderm whilst medium-to-mild phenotypes exhibit increasing coverage of ventral body wall by SBW, genito-urinary defects also apparent in lower abdomen including absence of bladder wall. Somite expression of *MyoD* and *myogenin* at E11.5 was similar to controls. BRDU labelling and apoptosis was also unchanged in the E13.5 ventral body wall, whereas ***Pitx2*** expression was lost (Zhang et al, 2014). Exomphalos not observed in some E18.5 embryos (Zhu et al, 2012).  • *Prx1-cre: Wntless* deletion from ventral body wall LPM leads to a larger abdomen but no exomphalos by E16.5 (Zhu et al, 2012).  • *Sox9-cre: Wntless* removed from somitic mesoderm, no ventral body wall defects reported although photograph of mutant at E16.5 suggests enlarged umbilical ring which has resolved by E18.5 (Snowball et al, 2015).  • *Msx2-cre: catnb* β-catenin is deleted from ventral body wall ectoderm from E12.5, this is a different phenotype to *Wntless* deletion leading authors to propose a block in Wnt secretion from ectoderm in *Msx-2-cre: Wntless* mutant rather than Wnt signalling from the ectoderm *per se.* However abdominal wall defect is observed with no ventral cover and lower abdomenal structures are missing (Zhang et al, 2014).  • *Dermo-1 cre: Lrp5*/*Lrp6* partially recapitulated the *Wntless* phenotype but embryos additionally exhibited an exomphalos with a thinner PBW and severely reduced LPM in the thorax compared to *dermo-1-cre* *Wntless* (Snowball et al, 2015). Signalling downstream is to intracellular effector Dvl (Etheridge et al, 2008).  Porcupine is located in the endoplasmic reticulum and regulates Wnt ligand secretion and activity.  • *Porcn*^-/-^ cell autonomous defect in Wnt ligand secretion. Ectopia cordis and exomphalos at E17.5, large areas of dermal atrophy overlain with thin ectodermal layer in thorax of a subset of null embryos, failure of sternum fusion. Also, thin transparent cover over abdomen associated with exomphalos phenotype (Barrott et al, 2011).  • *Lrp5*^-/-^: *Lrp6*^-/-^ double null specifically in mesoderm: suggests role(s) for canonical Wnt signalling in the closure of the entire body wall as embryos exhibit ectopia cordis as well as exomphalos (Joeng et al, 2011; Snowball et al, 2015).  • *Gsk3*^-/-^: partial split sternum phenotype (Liu et al. 2007), also impacts HH signalling. |
| **Tissue polarity and non-canonical Wnt** |  |
| ***Fat4, Dchs1***  *Fat 4^-/-^ : ∆ exon 3*  *Dchs1^-/-^ : ∆ exon 2* | • Planar polarity pathway, heterophilic protocadherin interactions, gradients of Fat4 and Dchs1 protein polarises cells within the sternal mesenchyme. Where mosaic of *Dchs1* null generated, mosaic of 3% of null mesenchymal cells can lead to a 30% wider sternum (Mao et al, 2016). |
| ***Celsr1***  *Crash (Crsh)* missense mutation in protocadherin repeat, ENU chemically induced | • Exomphalos at E16.5 axis in Balb/C background (66% penetrance), C. Formstone, personal communication.  • Low penetrance of shortened and skewed body axis associated with open ventral abdominal wall in double heterozygotes of *Crash^-/+^* with either Scribble *Circle-tail* ^-/+^ (10%) or Vangl2 *Loop-tail*^-/+^ (11%) C3H-HeH background, no limb defects (Murdoch et al, 2014). |
| ***Scribble***  *Circletail (Crc)* nonsense mutation, premature truncation of protein  *Scribble* ^flox/flox^ ∆ exon 4-13 | • Carnaghan and colleagues (2013) reported that VBW defect was exomphalos.  • 30% *Scribble* *circle-tail* ^-/+^ : *Vangl2 Loop-tail* ^-/+^ exhibit abdominal VBW defect with shortened and skewed body axis and hindlimb truncations (Murdoch et al, 2014), potential interaction with Wnt-PCP pathway.  • *Scribble* ^flox/flox^ : 54% exhibit exomphalos with germline cre deleter (Pearson et al, 2011). |
| ***Ptk7***  *Chuzhoi* mutant Splice mutation  ENU chemically induced | • 48% of homozygotes at E16.5 or older exhibited abdominal VBW defect with shortened and skewed body axis and truncated hindlimbs. Reduced levels of mutated Chuzhoi in membrane (Paudyal et al, 2010).  • Phenotype also seen in independent Ptk7 mouse mutant generated in gene-trapping screen (Lu et al. 2004). |
| ***Vangl2***  *Vangl1^-/-^ gene trap*  *Vangl2^fllox/flox^ Δ exon2-4*  *Vangl2 Loop-tail (Lp)* missense mutation in exon 8 | • Embryo turning fails in *Vangl1*^-/-^:*Vangl2*^-/-^ embryos with loss of asymmetric **Pitx2** expression, which plays a role in breaking left-right asymmetry in the node (Song et al, 2010). E18.5 Vangl2^-/-^ - shortened body (Andre et al, 2012). Double heterozygote phenotypes, *Celsr1 or Scribble* heterozygotes crossed with *Vangl2* *loop-tail* heterozygote - low penetrance of shortened and skewed body axis associated with open ventral abdominal wall *(*Murdoch et al, 2014). |
| **Ryk**  *Ryk^-/-^ -* Bgeo insert ∆ Ex-TM domains  *Ryk^-/+^ Wnt5a^-/-^*  *Ryk^-/-^/Vangl2^-/-^* | Functions with Frizzled/Vangl2 in limb extension (Green, Nusse, van Amerongen, 2014).  • Delay in SBW enclosure in *Ryk*^-/+^:*Wnt5a*^-/-^ mutant (E14.5) (Andre et al, 2012, Supplementary Figures).  • Open ventral body in Vangl2^-/-^:Ryk^-/-^ with shortened body, possible failure of embryo turning. Vangl2^-/-^ : Ryk^-/-^ double mutants sternum is shorter and wider and not fully closed (Andre et al, 2012). |
| ***Ror1, Ror2***  *Ror1^-/-^ neo insertion (*C57BL6)  *Ror2^-/-^* : neo insertion exon including Ig-like domain  *(*C57BL6)  *Ror1^flox/flox^ EIIA-cre* (Sv129/C57BL6)  *Ror2^flox/flox^ EIIA-cre* (Sv129/C57BL6) | Functions with Frizzled/Vangl2 in limb extension (Green, Nusse,van Amerongen, 2014).  • *Ror1*^-/-^ *Ror2*^-/-^ (C57BL6) exhibits sternal shortening and differentiation defects in all 4 pups analysed. Pubic narrowing in 25% of pups, n=4, authors suggest the latter might be linked to *Hoxd12* function, no exomphalos observed (Nomi et al, 2001).  • *Ror1^flox/flox^ Ror2^flox/flox^* ubiquitous *EIIA-cre* (Sv129/C57BL6) most embryos die at E15.5. Delayed SBW enclosure at E12.5 and E13.5 as well as posterior truncations. Phenotypes reported as similar to *Wnt5a* null (Ho et al, 2012).  • *Ror1^flox/flox^ Ror2^flox/flox^* ubiquitous *EIIA-cre* double knock-out fibroblasts and Wnt5a knock-out fibroblasts show deficits in Dvl2 phosphorylation, blocking antibodies against Ror1 and Ror2 also blocked Dvl2 phosphorylation (Ho et al, 2012).  • *ROR2* mutations associated with Robinow Syndrome involving disproportionate limb and short stature as well as genital hypoplasia (Lima et al, 2022). |
| ***Wnt5a***  *Wnt5a*^-/-^ neo insertion into exon 2  *Wnt5a ^tm1.1Homy^* (RBRCo4609) crossed with Rosa Cre-ERT2 | • Small abdominal herniation at E16.5 and E18.5 (Zhu et al, 2012)  • Split sternum in *Wnt5a^-/-^* embryos (Andre et al, 2012)  • *Wnt5a*^-/- (C57BL6)^ exhibit truncated posterior regions including genital tubercule and tail but VBW defect not reported (Yamaguchi et al, 1999), delayed SBW closure suggested in photographs presented by Ho et al, 2012.  • *Wnt5a*^-/-^  creER: cre activation from E8-E11, truncated limbs and possible abdominal herniation (Fig.1, Nakata et al, 2022).  • *WNT5a* mutations associated with Robinow Syndrome involving disproportionate limb and short stature as well as genital hypoplasia (Person et al, 2010). |
| **Other membrane proteins** |  |
| ***Podocalyxin*** (PODXL)  *Podxl*^-/-^ neo insertion ∆exons5-8 | •13/14 *Podxl*^-/-^ embryos exhibit exomphalos at E17, resolving to 30% incidence at birth. 8/35 heterozygous embryos have exomphalos at E17 resolving to 0% incidence at birth (Doyonnas et al, 2001). |
| ***Tmem67*** (Meckelin)  Frizzled-like 7TM protein | • Delayed SBW enclosure in 20% of E15.5 embryos, reduced A-P length at E18.5, no VBW defects presented by newborn pups. Co-localises with *Ror2*. Authors suggest plays a role in Wnt5a/Ror2 signalling which impacts on posterior tissue movements. Tmem67 binds Wnt ligands and Ror2. Needed for phosphorylation of Ror2 and Wnt-PCP function. Loss of Tmem67 linked to increased canonical Wnt signalling (Abdelhamad et al, 2015). |
| ***Extracellular matrix remodelling*** |  |
| ***ACLP***  *ACLP*^-/-^  ∆exon1 and exons 9-14 | • E13.5 *ACLP* null embryos exhibit gastroschisis, therefore, defect occurs prior to return of midgut to abdominal cavity following physiological herniation. Potential proliferation defect of fibroblasts (Layne et al, 2001, Danzer 2010). |
| ***Bmp1*** (mammalian Tolloid-like)  *Bmp1*^tm1^ TK and neo insertions | • *Bmp1*^tm1/tm1^ n=4/6 E16.5 and n=25/30 E17.5 embryos exhibit VBW defect with absence of membrane covering over intestines from E13.5.  • Mesenchymal component of amnion at E13.5 in knock-out embryos contains thin collagen fibres which are organised in a ‘barbed wire’ fashion rather than individual fibrils. *Bmp1*^tm1/tm1^ fibroblasts in culture assemble less ECM (Suzuki et al, 1996) |
| I**ntracellular proteins** |  |
| ***Amt*** | Aminomethyl transferase (Amt) is a component of an enzymatic complex which breaks down glycine to donate one-carbon units to Tetrahydrofolate (THF), this reaction is essential for folate metabolism (Kikuchi et al, 2008). Narisawa et al (2012) present one image of an *Amt* mouse mutant *Amt^-/-^,* with an obvious VBW defect. This *Amt^-/-^* mutant was generated via a gene trap leading to a truncated transcript lacking exons 3-9, C57BL6 background. Penetrance was not reported. International Mouse Phenotyping consortium (IMPC) webpage <https://www.mousephenotype.org/data/imageComparator?parameter_stable_id=IMPC_GEO_050_001&acc=MGI:3646700> shows a different *Amt* mouse mutant (homozygote) with a severe VBW defect. *Amt^em1(IMPC)Tcp^* is endonuclease-mediated (CRISPR-Cas9 edit) carrying a 1051-bp deletion of chromosome 9 at site of *Amt* gene, C57BL6 background. |
| ***β-catenin***  *β-catenin*^flox/flox^ del exon 2-6 (loss of function mouse: *β-catenin*^LOF^)  *Hoxb6*-cre; LPM-specific  En1-cre; ventral dermal precursors  *Msx2*-cre; ectoderm-specific but also tail-bud  *β-catenin*^flox/flox^ del exon 3 (gain of function mouse: *β-catenin*^GOF^) – constitutively active. | Signalling hub for canonical Wnt as well as cadherin-based cell adhesion. Nuclear fraction alters gene transcription, cytoplasmic fraction links cadherins to actin cytoskeleton.  • *Hoxb6-cre*-*β-catenin*^LOF^: active within LPM by E8, increased cell death in LPM by E10.5 (Ohtola et al, 2008).  • *En1 (engrailed1)-cre*-*β-catenin*^LOF^: active by E10.5: SBW enclosure is delayed compared to control at E14.5. Loss of *Dermo-1* expression by E11.5 which defines the sub-ectodermal flank and ventral mesenchyme. Decreased VBW proliferation at E11.5. Sternum wider and thinner at E16.5– dermal cells specified to cartilage/change in convergent-extension movements? (Ohtola et al, 2008).  • *En1-cre* - *β-catenin*^GOF^: increased *Dermo-1* expression and thickened ventral body wall at E11.5 demonstrating role of *β-catenin* in ventral mesenchyme growth and survival (Ohtola et al, 2008).  • *Msx2-cre*: - *β-catenin*^GOF^ ectopic ectodermal outgrowths observed on ventral abdomen and small ectopic limbs in flank region. Authors propose that ectopic ectodermal outgrowths/ectopic limbs sequester LPM away from ventral abdomen which impacts on the efficiency of SBW closure. This hypothesis is supported by *Msx2*-cre: *β-catenin*^LOF^ which resulted in embryos with truncated forelimbs and no hindlimbs. These embryos closed their VBW early at E14.5 (Zhu et al, 2014). |
| ***Calreticulin (Calr)***  *Calr*^-/-^ antisense insertion of neo into start site | Roles identified in other tissues: Ca2+ signalling, ER-folding of glycoproteins, canonical Wnt signalling (Groenendyk & Michalak, 2014; Serin et al. 2021), TGFβ signalling (Karimzadeh & Opas, 2017), Integrins & cell adhesion (Coppolino et al.1997), secreted BMP antagonist (De Almeida et al. 2017). Also see Rauch et al, 2000. |
| ***Dermatan Sulphate Epimerase 1 (Dse1)***  Pure NFR background  Dse^-/-^ neo insertion exon 2 | One of two enzymes (Dse1 & Dse2). Generates Iduronic Acid in extracellular matrix/proteoglycan component Dermatan Sulphate, Iduronic Acid induces migration and proliferation of epidermal keratinocytes (Radek, Taylor and Gallo, 2009) and aortic smooth muscle cells (Bartolini et al, 2013) as well as collagen maturation (Maccarana et al, 2009).  • 16% of null embryos exhibited exomphalos at E18.5, no apparent difference in the size of the umbilical ring. Epidermal thickening apparent from E16.5 onwards, Western blots showed K5 (marker of epidermal basal layer) increased significantly but not K1 (marker of epidermal suprabasal layer), (Gustafsson et al, 2014). Knockout of homologue, Dse2, does not result in VBW defects (Bartolino et al. 2012). |
| ***Filamin A (Flna)***  *Dilp2* ENU chemically induced nonsense mutation in exon 44  C57BL6 background | Has over 100 binding partners and many functions. Roles include Wnt, TGFβ signalling and ciliogenesis (Sasaki et al. 2001; Adams et al, 2012; Nishita et al. 2006). Associates with small G-proteins e.g. **RhoA** (Lian et al, 2016). *Dilp2* mouse mutant lacks the last 244 amino acids of Flna protein. Nonsense mediated decay however is proposed mechanism for low level of transcript in male mice. No filamin protein detectable in hemizygous males.  • *Dilp2* mouse (C57BL6) - no migration or growth defects observed in cultured *Dilp2* fibroblasts. Male *Dilp2* embryos exhibited VBW defect at E14.5, lethal by E15.5, possibly due to major heart defect. *Dilp2* carrier females at same stage exhibited delay in closure and fusion of the sternal bars, when fused the sternum is wider and shorter (Hart et al, 2006).  • *Dilp2* mouse (129/SvJ) - reduced cell proliferation (Ki67/BrdU staining) at ventral midline of thorax as well as reduced caspase-3 staining suggesting whilst tissue growth is reduced, ventral midline cells survive compared to wild-type (Lian et al, 2017). |
| ***Filamin A and Formin-2***  *Dilp2*  *Formin-2^-/-^ :* neo insertion ∆ proline exon | Flna and Formin-2 function together to impair **Lrp6** endocytosis and disrupt **canonical Wnt** signalling, most likely through diminished **β-catenin** transfer to the nucleus (Lian et al, 2016), but they do have other roles such as integrin signalling.  • *Flna*/*Formin-2* double mutant (Lian et al, 2017) exhibits open thorax and abdomen as well as shortening of the gut. Formin-2 mutant has no VBW phenotype (Lian et al, 2017).  • 50% reduction in cell proliferation and reduced levels of Desmin-positive myogenic/myofibroblast cells, authors suggest altered cell differentiation. Flna and Formin-2 proteins physically interact (Lian et al, 2017).  • Cell death (caspase-3 staining) at the ventral midline of the thorax in the double mutant is significantly higher than *Flna* mutant although significantly reduced compared to wild-type (Lian et al, 2017). |
| ***Folate Binding Protein 1***  *Folbp1^-/-^:* neomycin insertion into exon 2 | Survival/development may be dependent on genetic background. In studies by Piedrahita et al. 1999, *Folbp1^-/-^* embryos died by E10. |
| ***Hrs***  *Hrs^-/-^ neo insertion* ∆ exon 2-4 | • E9.5 ventral side of highly disorganised embryo was exposed outside of the yolk sac (Komada & Soriano, 1999)  Rostral and lateral-to ventral folding affected. Heart primordia do not fuse (Cardia bifida). Phenotype resembles *Gata4* mutants. *Gata4* was expressed normally in *Hrs*^-/-.^ |
| ***IKKα***  *IKK*α^-/-^: deletion of exon containing the  adenosine 59-triphosphate binding site (amino acids 192 to 212) via LacZ and Neo insertion | Regulates the Nuclear factor kB (NF-kB) transcription factor. Major defect in epidermal stratification associated with exomphalos in *IKK*^-/-^ embryos (Hu et al, 1999) was not observed in an epidermal-specific knockout (Gareus et al, 2007). Also exhibits bifurcated sternum. |
| ***Trip11*** (GMAP-210)  *Trip11*^-/-^ gene trap mouse- AJ0290 allele  *Trip11*^-/-^ ENU induced nonsense mutation | • AJ0290 gene trap allele: small umbilical hernia or exomphalos, partial penetrance reported. Interacts with IFT20, a cilia protein involved in intraflagellar transport, cilia defects but VBW not analysed (Follit et al, 2008).  • ENU nonsense mutation: also shortened body axis (Follit et al, 2008, Fig.4). |
| ***Male-abnormal 21 like 2***  *Mab21l2*^-/-^ neo insertion | • *Mab21l2^-/-^* lethal by E14.5, drastic reduction in thickness of VBW by E12.5, significantly reduced cell proliferation (BrdU) in E11.5 VBW, cell survival unchanged. Expression *of Bmp4, Dlx1,-2, -3 &-5, Msx1 & 2, Alx1 (Cart1), -3 & -4* unaffected in mutants (Yamada et al, 2004). |
| ***Mek1 and Mek2***  *Mek1^-/-^ :* Rosaβgeo insertion after exon 26  *Mek2^-/-^ :* neo insertion ∆ exon 4-6 | • Mesenchyme cre: *Mek1*^-/-^ with *Mek2*^-/-^: E14.5 **100% penetrance** (Boucherat et al, 2014). |
| ***Mekk4***  Kinase inactive mutant-K1361R | • *Mekk4*-K1361R. In codon 1361, a single-nucleotide change from AAG to AGG produced the replacement of the active site lysine by an arginine (Abell et al, 2005). Thoracic curvature defects including abnormal curvature of sternum are likely to be due to abnormal formation of SBW rather than intrinsic alterations in skeletal development. |
| ***p57kip2* (*CDKN1C*)**  *p57kip2*^-/-^ : neo insertion ∆ exon 1,2 | Paternally imprinted with expression from the maternal allele (Matsuoka et al, 1996).  • Yan et al (2007) - 40% of knock-out embryos exhibited shortening of the intestine, n=1 umbilical hernia, mixed background C57BL6-129/Sv.  • Zhang et al (1997) - a number of knock-out embryos exhibit exomphalos from E16.5 along with malrotation of the intestines, others exhibited an umbilical hernia. Knock-out embryos also had shorter bodies and abdominal muscle does not reach the ventral midline although muscle differentiation appeared normal. Skin differentiation delayed. C57BL6 background. |
| ***Presenilin-1***  Information on mouse mutant unavailable on www.med.kuleuven.ac.be | • Shen et al (1997) describe shortened abdomen/lower abdomen and tail from E9.5. Dermamyotome and sclerotome of early somite appear to develop normally.  • 30% of embryos exhibit abdominal wall herniations of various sizes filled with small intestine (Hartmann, Strooper and Saftig, 1999). |
| ***SuFu***  SuFu neo(frt)Ex456(fl)  and EIIa-cre deleter  SuFu^T396I/T396I^ | • *SuFu* ^Ex456 (fl)/Ex456(fl)^ EIIa conditional knock-out - hypomorphic allele, no evidence of ectopic HH signalling rather the observed processing defects for multiple GLI proteins indicate more complex scenario (Hoelzl et al, 2017). SuFu is an inhibitor of Shh signalling and regulates levels of Gli proteins.  • Exomphalos not observed in *SuFu*^T396I/T396I^ mice, only processing of Gli3 is disrupted in these mice (Makino et al. 2015). |
| **Cytoskeletal components:** |  |
| ***Caldesmon***  *Caldesmon*^-/-^ : neo insertion ∆exon2-stop codon | • Striated muscle migration normal but apparently thickened epidermis adjacent to the herniation. Panniculus carnosus muscle does not reach ventral midline and transversus abdominis muscle is thinner with disorganised with less pronounced connective tissues. Cultured *Caldesmon* null fibroblasts exhibited similar migration rates similar to controls. (Putz et al, 2021). |
| ***Cp110***  *Cp110*^-/-^: ablation of exon 5 (neo insertion) | Negative regulator of cilia biogenesis. Proposed mechanism: Impaired Hedgehog signalling (Yadav et al, 2016) |
| ***Ift25***  *Ift2*5^neo^: deletion of exon 3 via neo/LoxP insertion  *Ift25* ^null1^ : conversion via germline cre | Member of small G-protein family, vertebrate specific intraflagellar transport (IFT) protein. Exomphalos phenotype, proposed mechanism: disrupted transport of Hedgehog within the primary cilium (Keady et al, 2012). |
| ***NMHCII***  ***Myh10***  (*NMIIB: missense mutation*)  B^R709C^ – removal of neo that has been inserted into intron 5’ of exon 16 | B^R709C^ - neo insertion removed so mutant *Myh10* expression increased to wild-type levels.  • B^+^/B^R709C^ - 50% penetrance exomphalos, loss of skeletal muscle around the lateral areas of the diaphragm. B^R709C^ / B^R709C^ embryos: reduced apoptosis in the closing sternum at E14.5 (Ma and Adelstein, 2014).  • Tullio et al, 1997: ablation of NMIIB does not lead to VBW phenotype suggesting that NMIIA alone can support VBW closure and that B^R709C^ mutation interferes with NMIIA function. |
| ***Luzp1***  *Luzp*^Lacz/LacZ^ | Regulates the actin cytoskeleton through interactions with **Filamin A** (Wang and Nakamura, 2019). Delay in abdominal wall closure at E14.5 resolves by birth (Hsu et al, 2008). |
| ***ROCK1/2***  *ROCK1^-/-^* - gal/neo insertion ∆ exon 2 and 3  *ROCK2^-/-^* - gal/neo insertion ∆ exon 2 and 3  *ROCK* isoform kinase dead (*ROCK^kd/kd^*) | Gal/neo insertion disrupts kinase domain.  • *ROCK1^-/-^* : exomphalos and absence of epidermal ridge surrounding the umbilicus, range of severity (Shimizu et al, 2005).  • *ROCK2^-/-^* ^:^ C57BL6/129/SvJ - no exomphalos (Thumkeo et al, 2005) whereas *ROCK2^-/-^* C57BL6 exhibits exomphalos (Thumkeo et al, 2005), thus genetic background important.  •*ROCK1^-/+ :^ROCK2^-/+^* double heterozygotes exhibit exomphalos whereas *ROCK1^-/- :^ROCK2^-/-^* double homozygote mutants do not exhibit exomphalos and survive better than both double heterozygotes and individual *ROCK1* and *ROCK2* null mutants, which exhibit exomphalos ,suggesting that one *ROCK* can compensate for the other (Thumkeo et al, 2005).  • *ROCK*^kd/kd^ embryos exhibit exomphalos, suppression of pMLC and LIMK/Cofilin pathways in umbilical ring epithelial cells reported (Shi and Wei, 2022).  • ROCK inhibition in chick embryos leads to exomphalos (Duess et al, 2016, 2020). |
| ***Specc1l***  *Specc1l^CCD2/CCD2^*  CRISPR-generated deletions within CCD2 coiled coil domain predicted to interact with microtubules, gain-of-function phenotype.  *Specc1lΔ234*- CRISPR generated in-frame deletion in CCD2 domain.  *Specc1lΔ576-* CRISPR generated in-frame deletion in CCD2 domain. | • *Specc1lΔ234, Specc1lΔ576*, both led to exomphalos (70% and 50% respectively). Heterozygotes also exhibit exomphalos (3% and 22%). *Specc1L* ^+/CCD2^ also exhibit exomphalos (12%). Null allele lacking any protein (*Specc1lΔEx4*) did not exhibit exomphalos.  • *Specc1L* ^CCD2/CCD2^ embryos: cleft palate phenotype was associated with reduced acto-myosin association and abnormally bundled actin in palate mesenchymal cells. SPECC1L and MYH10 were found to exist together within a protein complex (Goering et al, 2022). Mutations alter this interaction which in turn result in altered actin cytoskeleton distribution. |
| ***Shroom*** | *Shroom****^-/-^*** - gene trap with LacZ reporter (Hildebrand and Soriano, 1999) |
| ***Nuak1/2 (Omphk1/2)***  *Nuak1^-/-^ and Nuak2^-/-^:* LacZ knock-in after start codon | • *Nuak1^-/-^* : PBW forms normally at E9.5, no defect observed at E12.5 but VBW defect exhibited by E14.5. Failure to fuse abdominal bands but no sternal defect and thorax is closed.  • *Nuak1^-/-^: Nuak2^-/-^* double mutant phenotype same as *Nuak1^-/-^* (Hirano et al, 2006; Ohmura et al, 2012) as expected as Nuak2 is not expressed in PBW or SBW. |
| **Inhibitory Neurotransmitter system** |  |
| ***Gad1, Gad2, VGAT***  *Gad 1****^-/-^ :***  GFP insertion intron 1  *Gad1* encodes Gad67.  *Gad2*^-/-^: neo insertion ∆ exon 3  *Gad2* encodes GAD65 | • *Gad1*^-/-^ : 59% penetrance exomphalos (Oh et al. 2010; Saito et al. 2010; Wojcik et al. 2006; Kakizaki, Oriuchi, Yanagawa, 2015). However, *Gad2*^-/-^ has no VBW phenotype (Asada et al. 1996; Kash et al. 1997)  • *Gad2*^-/-^:*Gad*1^-/-^ double knockout (100%) exomphalos is more severe than *Gad1*^-/-^ with shorter body axis similar to KCC2 knock-out (see below). Rat *Gad2*^-/-^:*Gad*1^-/-^ has no exomphalos phenotype (Jiang et al. 2022).  High incidence of cleft palate in these mutants also suggests a role for GABA signalling in tissue scale movements (Kakizaki, Oriuchi, Yanagawa, 2015). |
| ***VGAT***  *VGAT^-/-^* : floxneo ∆ exon 2/3 | • *VGAT^-/-^* : phenotype more severe than *Gad1/2* double knockout implicating a requirement for both GABAergic and glycinergic signalling (Kakizaki, Oriuchi, Yanagawa, 2015). |
| ***KCC2***  *KCC2^-/-^ -* neo insertion ∆exon 5 | KCC2 establishes post synaptic chloride gradients required for synaptic inhibition by GABA or glycine  • In *KCC2*^-/-^ embryos (E17.5) growth of internal organs also appears affected and body axis is shortened (Hubner et al, 2001). |

Footnote: CT is connective tissue, VBW is ventral body wall, LPM is lateral plate mesoderm, SBW is secondary body wall, PBW is primary body wall. Mutant VBW phenotypes have been interpreted based on human VBW pathologies, see also Table 2.

**References**

1. Abdelhamed ZA, Natarajan S, Wheway G, et al. The Meckel-Gruber syndrome protein TMEM67 controls basal body positioning and epithelial branching morphogenesis in mice via the non-canonical Wnt pathway. *Dis Model Mech*. Jun 2015;8(6):527-41. doi:10.1242/dmm.019083

2. Abell AN, Rivera-Perez JA, Cuevas BD, et al. Ablation of MEKK4 kinase activity causes neurulation and skeletal patterning defects in the mouse embryo. *Mol Cell Biol*. Oct 2005;25(20):8948-59. doi:10.1128/MCB.25.20.8948-8959.2005

3. Adams M, Simms RJ, Abdelhamed Z, et al. A meckelin-filamin A interaction mediates ciliogenesis. *Hum Mol Genet*. Mar 15 2012;21(6):1272-86. doi:10.1093/hmg/ddr557

4. Aldeiri B, Roostalu U, Albertini A, Wong J, Morabito A, Cossu G. Transgelin-expressing myofibroblasts orchestrate ventral midline closure through TGFβ signalling. *Development*. Sep 15 2017;144(18):3336-3348. doi:10.1242/dev.152843

5. Andre P, Wang Q, Wang N, et al. The Wnt coreceptor Ryk regulates Wnt/planar cell polarity by modulating the degradation of the core planar cell polarity component Vangl2. *J Biol Chem*. Dec 28 2012;287(53):44518-25. doi:10.1074/jbc.M112.414441

6. Angelozzi M, Pellegrino da Silva R, Gonzalez MV, Lefebvre V. Single-cell atlas of craniogenesis uncovers SOXC-dependent, highly proliferative, and myofibroblast-like osteodermal progenitors. *Cell Rep*. Jul 12 2022;40(2):111045. doi:10.1016/j.celrep.2022.111045

7. Arostegui M, Scott RW, Böse K, Underhill TM. Cellular taxonomy of Hic1. *Nat Commun*. Aug 25 2022;13(1):4989. doi:10.1038/s41467-022-32695-1

8. Asada H, Kawamura Y, Maruyama K, et al. Cleft palate and decreased brain gamma-aminobutyric acid in mice lacking the 67-kDa isoform of glutamic acid decarboxylase. *Proc Natl Acad Sci U S A*. Jun 10 1997;94(12):6496-9. doi:10.1073/pnas.94.12.6496

9. Barrott JJ, Cash GM, Smith AP, Barrow JR, Murtaugh LC. Deletion of mouse Porcn blocks Wnt ligand secretion and reveals an ectodermal etiology of human focal dermal hypoplasia/Goltz syndrome. *Proc Natl Acad Sci U S A*. Aug 02 2011;108(31):12752-7. doi:10.1073/pnas.1006437108

10. Barrow JR, Capecchi MR. Targeted disruption of the Hoxb-2 locus in mice interferes with expression of Hoxb-1 and Hoxb-4. *Development*. Dec 1996;122(12):3817-28. doi:10.1242/dev.122.12.3817

11. Bartolini B, Thelin MA, Svensson L, et al. Iduronic acid in chondroitin/dermatan sulfate affects directional migration of aortic smooth muscle cells. *PLoS One*. 2013;8(7):e66704. doi:10.1371/journal.pone.0066704

12. Bhattaram P, Penzo-Méndez A, Sock E, et al. Organogenesis relies on SoxC transcription factors for the survival of neural and mesenchymal progenitors. *Nat Commun*. Apr 12 2010;1(1):9. doi:10.1038/ncomms1008

13. Boucherat O, Nadeau V, Bérubé-Simard FA, Charron J, Jeannotte L. Crucial requirement of ERK/MAPK signaling in respiratory tract development. *Development*. Aug 2014;141(16):3197-211. doi:10.1242/dev.110254

14. Boylan M, Anderson MJ, Ornitz DM, Lewandoski M. The Fgf8 subfamily (Fgf8, Fgf17 and Fgf18) is required for closure of the embryonic ventral body wall. *Development*. Oct 19 2020;147(21)doi:10.1242/dev.189506

15. Brewer S, Williams T. Finally, a sense of closure? Animal models of human ventral body wall defects. *Bioessays*. Dec 2004;26(12):1307-21. doi:10.1002/bies.20137

16. Carnaghan H, Roberts T, Savery D, et al. Novel exomphalos genetic mouse model: the importance of accurate phenotypic classification. *J Pediatr Surg*. Oct 2013;48(10):2036-42. doi:10.1016/j.jpedsurg.2013.04.010

17. Carter MG, Johns MA, Zeng X, et al. Mice deficient in the candidate tumor suppressor gene Hic1 exhibit developmental defects of structures affected in the Miller-Dieker syndrome. *Hum Mol Genet*. Feb 12 2000;9(3):413-9. doi:10.1093/hmg/9.3.413

18. Cheng W, Jacobs WB, Zhang JJ, et al. DeltaNp63 plays an anti-apoptotic role in ventral bladder development. *Development*. Dec 2006;133(23):4783-92. doi:10.1242/dev.02621

19. Ching BJ, Wittler L, Proske J, et al. p63 (TP73L) a key player in embryonic urogenital development with significant dysregulation in human bladder exstrophy tissue. *Int J Mol Med*. Dec 2010;26(6):861-7. doi:10.3892/ijmm_00000535

20. Collier A, Liu A, Torkelson J, et al. Gibbin mesodermal regulation patterns epithelial development. *Nature*. Jun 2022;606(7912):188-196. doi:10.1038/s41586-022-04727-9

21. Compagni A, Logan M, Klein R, Adams RH. Control of skeletal patterning by ephrinB1-EphB interactions. *Dev Cell*. Aug 2003;5(2):217-30. doi:10.1016/s1534-5807(03)00198-9

22. Constam DB. Regulation of TGFβ and related signals by precursor processing. *Semin Cell Dev Biol*. Aug 2014;32:85-97. doi:10.1016/j.semcdb.2014.01.008

23. Coppolino MG, Woodside MJ, Demaurex N, Grinstein S, St-Arnaud R, Dedhar S. Calreticulin is essential for integrin-mediated calcium signalling and cell adhesion. *Nature*. Apr 24 1997;386(6627):843-7. doi:10.1038/386843a0

24. Danzer E, Layne MD, Auber F, et al. Gastroschisis in mice lacking aortic carboxypeptidase-like protein is associated with a defect in neuromuscular development of the eviscerated intestine. *Pediatr Res*. Jul 2010;68(1):23-8. doi:10.1203/PDR.0b013e3181e17c75

25. Davy A, Aubin J, Soriano P. Ephrin-B1 forward and reverse signaling are required during mouse development. *Genes Dev*. Mar 01 2004;18(5):572-83. doi:10.1101/gad.1171704

26. De Almeida I, Oliveira NMM, Randall RA, Hill CS, McCoy JM, Stern CD. Calreticulin is a secreted BMP antagonist, expressed in Hensen's node during neural induction. *Dev Biol*. Jan 15 2017;421(2):161-170. doi:10.1016/j.ydbio.2016.12.001

27. Doyonnas R, Kershaw DB, Duhme C, et al. Anuria, omphalocele, and perinatal lethality in mice lacking the CD34-related protein podocalyxin. *J Exp Med*. Jul 02 2001;194(1):13-27. doi:10.1084/jem.194.1.13

28. Dravis C, Henkemeyer M. Ephrin-B reverse signaling controls septation events at the embryonic midline through separate tyrosine phosphorylation-independent signaling avenues. *Dev Biol*. Jul 01 2011;355(1):138-51. doi:10.1016/j.ydbio.2011.04.020

29. Duess JW, Gosemann JH, Puri P, Thompson J. Teratogenesis in the chick embryo following post-gastrulation exposure to Y-27632 -effect of Y-27632 on embryonic development. *Toxicol Appl Pharmacol*. Dec 15 2020;409:115277. doi:10.1016/j.taap.2020.115277

30. Duess JW, Puri P, Thompson J. Impaired cytoskeletal arrangements and failure of ventral body wall closure in chick embryos treated with rock inhibitor (Y-27632). *Pediatr Surg Int*. Jan 2016;32(1):45-58. doi:10.1007/s00383-015-3811-z

31. Dünker N, Krieglstein K. Tgfbeta2 -/- Tgfbeta3 -/- double knockout mice display severe midline fusion defects and early embryonic lethality. *Anat Embryol (Berl)*. Dec 2002;206(1-2):73-83. doi:10.1007/s00429-002-0273-6

32. Eggenschwiler J, Ludwig T, Fisher P, Leighton PA, Tilghman SM, Efstratiadis A. Mouse mutant embryos overexpressing IGF-II exhibit phenotypic features of the Beckwith-Wiedemann and Simpson-Golabi-Behmel syndromes. *Genes Dev*. Dec 01 1997;11(23):3128-42. doi:10.1101/gad.11.23.3128

33. Eng D, Campbell A, Hilton T, Leid M, Gross MK, Kioussi C. Prediction of regulatory networks in mouse abdominal wall. *Gene*. Dec 01 2010;469(1-2):1-8. doi:10.1016/j.gene.2010.08.008

34. Eng D, Ma HY, Xu J, Shih HP, Gross MK, Kioussi C. Loss of abdominal muscle in Pitx2 mutants associated with altered axial specification of lateral plate mesoderm. *PLoS One*. 2012;7(7):e42228. doi:10.1371/journal.pone.0042228

35. Essalmani R, Zaid A, Marcinkiewicz J, et al. In vivo functions of the proprotein convertase PC5/6 during mouse development: Gdf11 is a likely substrate. *Proc Natl Acad Sci U S A*. Apr 15 2008;105(15):5750-5. doi:10.1073/pnas.0709428105

36. Follit JA, San Agustin JT, Xu F, et al. The Golgin GMAP210/TRIP11 anchors IFT20 to the Golgi complex. *PLoS Genet*. Dec 2008;4(12):e1000315. doi:10.1371/journal.pgen.1000315

37. Gage PJ, Suh H, Camper SA. Dosage requirement of Pitx2 for development of multiple organs. *Development*. Oct 1999;126(20):4643-51. doi:10.1242/dev.126.20.4643

38. Gareus R, Huth M, Breiden B, et al. Normal epidermal differentiation but impaired skin-barrier formation upon keratinocyte-restricted IKK1 ablation. *Nat Cell Biol*. Apr 2007;9(4):461-9. doi:10.1038/ncb1560

39. Goering JP, Wenger LW, Stetsiv M, et al. In-frame deletion of SPECC1L microtubule association domain results in gain-of-function phenotypes affecting embryonic tissue movement and fusion events. *Hum Mol Genet*. Dec 17 2021;31(1):18-31. doi:10.1093/hmg/ddab211

40. Goldman DC, Donley N, Christian JL. Genetic interaction between Bmp2 and Bmp4 reveals shared functions during multiple aspects of mouse organogenesis. *Mech Dev*. 2009;126(3-4):117-27. doi:10.1016/j.mod.2008.11.008

41. Goldman DC, Hackenmiller R, Nakayama T, et al. Mutation of an upstream cleavage site in the BMP4 prodomain leads to tissue-specific loss of activity. *Development*. May 2006;133(10):1933-42. doi:10.1242/dev.02368

42. Green J, Nusse R, van Amerongen R. The role of Ryk and Ror receptor tyrosine kinases in Wnt signal transduction. *Cold Spring Harb Perspect Biol*. Feb 01 2014;6(2)doi:10.1101/cshperspect.a009175

43. Groenendyk J, Michalak M. Disrupted WNT signaling in mouse embryonic stem cells in the absence of calreticulin. *Stem Cell Rev Rep*. Apr 2014;10(2):191-206. doi:10.1007/s12015-013-9488-6

44. Grote P, Wittler L, Hendrix D, et al. The tissue-specific lncRNA Fendrr is an essential regulator of heart and body wall development in the mouse. *Dev Cell*. Jan 28 2013;24(2):206-14. doi:10.1016/j.devcel.2012.12.012

45. Gustafsson R, Stachtea X, Maccarana M, et al. Dermatan sulfate epimerase 1 deficient mice as a model for human abdominal wall defects. *Birth Defects Res A Clin Mol Teratol*. Sep 2014;100(9):712-20. doi:10.1002/bdra.23300

46. Hart AW, Morgan JE, Schneider J, et al. Cardiac malformations and midline skeletal defects in mice lacking filamin A. *Hum Mol Genet*. Aug 15 2006;15(16):2457-67. doi:10.1093/hmg/ddl168

47. Hartmann D, De Strooper B, Saftig P. Presenilin-1 deficiency leads to loss of Cajal-Retzius neurons and cortical dysplasia similar to human type 2 lissencephaly. *Curr Biol*. Jul 15 1999;9(14):719-27. doi:10.1016/s0960-9822(99)80331-5

48. Hildebrand JD, Soriano P. Shroom, a PDZ domain-containing actin-binding protein, is required for neural tube morphogenesis in mice. *Cell*. Nov 24 1999;99(5):485-97. doi:10.1016/s0092-8674(00)81537-8

49. Hilton T, Gross MK, Kioussi C. Pitx2-dependent occupancy by histone deacetylases is associated with T-box gene regulation in mammalian abdominal tissue. *J Biol Chem*. Apr 09 2010;285(15):11129-42. doi:10.1074/jbc.M109.087429

50. Hirano M, Kiyonari H, Inoue A, et al. A new serine/threonine protein kinase, Omphk1, essential to ventral body wall formation. *Dev Dyn*. Aug 2006;235(8):2229-37. doi:10.1002/dvdy.20823

51. Ho HY, Susman MW, Bikoff JB, et al. Wnt5a-Ror-Dishevelled signaling constitutes a core developmental pathway that controls tissue morphogenesis. *Proc Natl Acad Sci U S A*. Mar 13 2012;109(11):4044-51. doi:10.1073/pnas.1200421109

52. Hoelzl MA, Heby-Henricson K, Gerling M, et al. Differential requirement of SUFU in tissue development discovered in a hypomorphic mouse model. *Dev Biol*. Sep 01 2017;429(1):132-146. doi:10.1016/j.ydbio.2017.06.037

53. Hsu CY, Chang NC, Lee MW, et al. LUZP deficiency affects neural tube closure during brain development. *Biochem Biophys Res Commun*. Nov 21 2008;376(3):466-71. doi:10.1016/j.bbrc.2008.08.170

54. Hu Y, Baud V, Delhase M, et al. Abnormal morphogenesis but intact IKK activation in mice lacking the IKKalpha subunit of IkappaB kinase. *Science*. Apr 09 1999;284(5412):316-20. doi:10.1126/science.284.5412.316

55. Hübner CA, Stein V, Hermans-Borgmeyer I, Meyer T, Ballanyi K, Jentsch TJ. Disruption of KCC2 reveals an essential role of K-Cl cotransport already in early synaptic inhibition. *Neuron*. May 2001;30(2):515-24. doi:10.1016/s0896-6273(01)00297-5

56. Jayewickreme CD, Shivdasani RA. Control of stomach smooth muscle development and intestinal rotation by transcription factor BARX1. *Dev Biol*. Sep 01 2015;405(1):21-32. doi:10.1016/j.ydbio.2015.05.024

57. Jiang W, Kakizaki T, Fujihara K, et al. Impact of GAD65 and/or GAD67 deficiency on perinatal development in rats. *FASEB J*. Feb 2022;36(2):e22123. doi:10.1096/fj.202101389R

58. Joeng KS, Schumacher CA, Zylstra-Diegel CR, Long F, Williams BO. Lrp5 and Lrp6 redundantly control skeletal development in the mouse embryo. *Dev Biol*. Nov 15 2011;359(2):222-9. doi:10.1016/j.ydbio.2011.08.020

59. Johnson DR. Extra-toes: anew mutant gene causing multiple abnormalities in the mouse. *J Embryol Exp Morphol*. Jun 1967;17(3):543-81.

60. Kakizaki T, Oriuchi N, Yanagawa Y. GAD65/GAD67 double knockout mice exhibit intermediate severity in both cleft palate and omphalocele compared with GAD67 knockout and VGAT knockout mice. *Neuroscience*. Mar 12 2015;288:86-93. doi:10.1016/j.neuroscience.2014.12.030

61. Karimzadeh F, Opas M. Calreticulin Is Required for TGF-β-Induced Epithelial-to-Mesenchymal Transition during Cardiogenesis in Mouse Embryonic Stem Cells. *Stem Cell Reports*. May 09 2017;8(5):1299-1311. doi:10.1016/j.stemcr.2017.03.018

62. Kash SF, Johnson RS, Tecott LH, et al. Epilepsy in mice deficient in the 65-kDa isoform of glutamic acid decarboxylase. *Proc Natl Acad Sci U S A*. Dec 09 1997;94(25):14060-5. doi:10.1073/pnas.94.25.14060

63. Keady BT, Samtani R, Tobita K, et al. IFT25 links the signal-dependent movement of Hedgehog components to intraflagellar transport. *Dev Cell*. May 15 2012;22(5):940-51. doi:10.1016/j.devcel.2012.04.009

64. Kikuchi G, Motokawa Y, Yoshida T, Hiraga K. Glycine cleavage system: reaction mechanism, physiological significance, and hyperglycinemia. *Proc Jpn Acad Ser B Phys Biol Sci*. 2008;84(7):246-63. doi:10.2183/pjab.84.246

65. Kimura A, Inose H, Yano F, et al. Runx1 and Runx2 cooperate during sternal morphogenesis. *Development*. Apr 2010;137(7):1159-67. doi:10.1242/dev.045005

66. Kioussi C, Briata P, Baek SH, et al. Identification of a Wnt/Dvl/beta-Catenin --> Pitx2 pathway mediating cell-type-specific proliferation during development. *Cell*. Nov 27 2002;111(5):673-85. doi:10.1016/s0092-8674(02)01084-x

67. Kitamura K, Miura H, Miyagawa-Tomita S, et al. Mouse Pitx2 deficiency leads to anomalies of the ventral body wall, heart, extra- and periocular mesoderm and right pulmonary isomerism. *Development*. Dec 1999;126(24):5749-58. doi:10.1242/dev.126.24.5749

68. Klootwijk R, Franke B, van der Zee CE, et al. A deletion encompassing Zic3 in bent tail, a mouse model for X-linked neural tube defects. *Hum Mol Genet*. Jul 01 2000;9(11):1615-22. doi:10.1093/hmg/9.11.1615

69. Komada M, Soriano P. Hrs, a FYVE finger protein localized to early endosomes, is implicated in vesicular traffic and required for ventral folding morphogenesis. *Genes Dev*. Jun 01 1999;13(11):1475-85. doi:10.1101/gad.13.11.1475

70. Layne MD, Yet SF, Maemura K, et al. Impaired abdominal wall development and deficient wound healing in mice lacking aortic carboxypeptidase-like protein. *Mol Cell Biol*. Aug 2001;21(15):5256-61. doi:10.1128/MCB.21.15.5256-5261.2001

71. Leask A, Byrne C, Fuchs E. Transcription factor AP2 and its role in epidermal-specific gene expression. *Proc Natl Acad Sci U S A*. Sep 15 1991;88(18):7948-52. doi:10.1073/pnas.88.18.7948

72. Lee Y, Fryer JD, Kang H, et al. ATXN1 protein family and CIC regulate extracellular matrix remodeling and lung alveolarization. *Dev Cell*. Oct 18 2011;21(4):746-57. doi:10.1016/j.devcel.2011.08.017

73. Li A, Hardy R, Stoner S, Tuckermann J, Seibel M, Zhou H. Deletion of mesenchymal glucocorticoid receptor attenuates embryonic lung development and abdominal wall closure. *PLoS One*. 2013;8(5):e63578. doi:10.1371/journal.pone.0063578

74. Lian G, Dettenhofer M, Lu J, et al. Filamin A- and formin 2-dependent endocytosis regulates proliferation via the canonical Wnt pathway. *Development*. Dec 01 2016;143(23):4509-4520. doi:10.1242/dev.139295

75. Lian G, Kanaujia S, Wong T, Sheen V. FilaminA and Formin2 regulate skeletal, muscular, and intestinal formation through mesenchymal progenitor proliferation. *PLoS One*. 2017;12(12):e0189285. doi:10.1371/journal.pone.0189285

76. Lima AR, Ferreira BM, Zhang C, et al. Phenotypic and mutational spectrum of ROR2-related Robinow syndrome. *Hum Mutat*. Jul 2022;43(7):900-918. doi:10.1002/humu.24375

77. Lin CR, Kioussi C, O'Connell S, et al. Pitx2 regulates lung asymmetry, cardiac positioning and pituitary and tooth morphogenesis. *Nature*. Sep 16 1999;401(6750):279-82. doi:10.1038/45803

78. Liu KJ, Arron JR, Stankunas K, Crabtree GR, Longaker MT. Chemical rescue of cleft palate and midline defects in conditional GSK-3beta mice. *Nature*. Mar 01 2007;446(7131):79-82. doi:10.1038/nature05557

79. Lu MF, Pressman C, Dyer R, Johnson RL, Martin JF. Function of Rieger syndrome gene in left-right asymmetry and craniofacial development. *Nature*. Sep 16 1999;401(6750):276-8. doi:10.1038/45797

80. Lu X, Borchers AG, Jolicoeur C, Rayburn H, Baker JC, Tessier-Lavigne M. PTK7/CCK-4 is a novel regulator of planar cell polarity in vertebrates. *Nature*. Jul 01 2004;430(6995):93-8. doi:10.1038/nature02677

81. Ma X, Adelstein RS. A point mutation in Myh10 causes major defects in heart development and body wall closure. *Circ Cardiovasc Genet*. Jun 2014;7(3):257-65. doi:10.1161/CIRCGENETICS.113.000455

82. Maccarana M, Kalamajski S, Kongsgaard M, Magnusson SP, Oldberg A, Malmström A. Dermatan sulfate epimerase 1-deficient mice have reduced content and changed distribution of iduronic acids in dermatan sulfate and an altered collagen structure in skin. *Mol Cell Biol*. Oct 2009;29(20):5517-28. doi:10.1128/MCB.00430-09

83. Makino S, Zhulyn O, Mo R, et al. T396I mutation of mouse Sufu reduces the stability and activity of Gli3 repressor. *PLoS One*. 2015;10(3):e0119455. doi:10.1371/journal.pone.0119455

84. Manley NR, Barrow JR, Zhang T, Capecchi MR. Hoxb2 and hoxb4 act together to specify ventral body wall formation. *Dev Biol*. Sep 01 2001;237(1):130-44. doi:10.1006/dbio.2001.0365

85. Mao Y, Kuta A, Crespo-Enriquez I, et al. Dchs1-Fat4 regulation of polarized cell behaviours during skeletal morphogenesis. *Nat Commun*. May 05 2016;7:11469. doi:10.1038/ncomms11469

86. Maska EL, Cserjesi P, Hua LL, Garstka ME, Brody HM, Morikawa Y. A Tlx2-Cre mouse line uncovers essential roles for hand1 in extraembryonic and lateral mesoderm. *Genesis*. Aug 2010;48(8):479-84. doi:10.1002/dvg.20644

87. Matsumaru D, Haraguchi R, Miyagawa S, et al. Genetic analysis of Hedgehog signaling in ventral body wall development and the onset of omphalocele formation. *PLoS One*. Jan 20 2011;6(1):e16260. doi:10.1371/journal.pone.0016260

88. Matsumaru D, Haraguchi R, Moon AM, et al. Genetic analysis of the role of Alx4 in the coordination of lower body and external genitalia formation. *Eur J Hum Genet*. Mar 2014;22(3):350-7. doi:10.1038/ejhg.2013.160

89. Matsunobu T, Torigoe K, Ishikawa M, et al. Critical roles of the TGF-beta type I receptor ALK5 in perichondrial formation and function, cartilage integrity, and osteoblast differentiation during growth plate development. *Dev Biol*. Aug 15 2009;332(2):325-38. doi:10.1016/j.ydbio.2009.06.002

90. Mills AA, Zheng B, Wang XJ, Vogel H, Roop DR, Bradley A. p63 is a p53 homologue required for limb and epidermal morphogenesis. *Nature*. Apr 22 1999;398(6729):708-13. doi:10.1038/19531

91. Mo R, Freer AM, Zinyk DL, et al. Specific and redundant functions of Gli2 and Gli3 zinc finger genes in skeletal patterning and development. *Development*. Jan 1997;124(1):113-23. doi:10.1242/dev.124.1.113

92. Murdoch JN, Damrau C, Paudyal A, et al. Genetic interactions between planar cell polarity genes cause diverse neural tube defects in mice. *Dis Model Mech*. Oct 2014;7(10):1153-63. doi:10.1242/dmm.016758

93. Nakata M, Honda H, Iwama A, et al. Wnt5a plays a critical role in anal opening in mice at an early stage of embryonic development. *Pediatr Surg Int*. May 2022;38(5):743-747. doi:10.1007/s00383-022-05103-4

94. Narisawa A, Komatsuzaki S, Kikuchi A, et al. Mutations in genes encoding the glycine cleavage system predispose to neural tube defects in mice and humans. *Hum Mol Genet*. Apr 01 2012;21(7):1496-503. doi:10.1093/hmg/ddr585

95. Nerurkar NL, Mahadevan L, Tabin CJ. BMP signaling controls buckling forces to modulate looping morphogenesis of the gut. *Proc Natl Acad Sci U S A*. Feb 28 2017;114(9):2277-2282. doi:10.1073/pnas.1700307114

96. Nichol PF, Corliss RF, Tyrrell JD, Graham B, Reeder A, Saijoh Y. Conditional mutation of fibroblast growth factor receptors 1 and 2 results in an omphalocele in mice associated with disruptions in ventral body wall muscle formation. *J Pediatr Surg*. Jan 2011;46(1):90-6. doi:10.1016/j.jpedsurg.2010.09.066

97. Nishita M, Yoo SK, Nomachi A, et al. Filopodia formation mediated by receptor tyrosine kinase Ror2 is required for Wnt5a-induced cell migration. *J Cell Biol*. Nov 20 2006;175(4):555-62. doi:10.1083/jcb.200607127

98. Nomi M, Oishi I, Kani S, et al. Loss of mRor1 enhances the heart and skeletal abnormalities in mRor2-deficient mice: redundant and pleiotropic functions of mRor1 and mRor2 receptor tyrosine kinases. *Mol Cell Biol*. Dec 2001;21(24):8329-35. doi:10.1128/MCB.21.24.8329-8335.2001

99. Nottoli T, Hagopian-Donaldson S, Zhang J, Perkins A, Williams T. AP-2-null cells disrupt morphogenesis of the eye, face, and limbs in chimeric mice. *Proc Natl Acad Sci U S A*. Nov 10 1998;95(23):13714-9. doi:10.1073/pnas.95.23.13714

100. Ogi H, Suzuki K, Ogino Y, et al. Ventral abdominal wall dysmorphogenesis of Msx1/Msx2 double-mutant mice. *Anat Rec A Discov Mol Cell Evol Biol*. May 2005;284(1):424-30. doi:10.1002/ar.a.20180

101. Oh WJ, Westmoreland JJ, Summers R, Condie BG. Cleft palate is caused by CNS dysfunction in Gad1 and Viaat knockout mice. *PLoS One*. Mar 19 2010;5(3):e9758. doi:10.1371/journal.pone.0009758

102. Ohmura T, Shioi G, Hirano M, Aizawa S. Neural tube defects by NUAK1 and NUAK2 double mutation. *Dev Dyn*. Aug 2012;241(8):1350-64. doi:10.1002/dvdy.23816

103. Ohtola J, Myers J, Akhtar-Zaidi B, et al. beta-Catenin has sequential roles in the survival and specification of ventral dermis. *Development*. Jul 2008;135(13):2321-9. doi:10.1242/dev.021170

104. Orioli D, Henkemeyer M, Lemke G, Klein R, Pawson T. Sek4 and Nuk receptors cooperate in guidance of commissural axons and in palate formation. *EMBO J*. Nov 15 1996;15(22):6035-49.

105. Oshima M, Oshima H, Taketo MM. TGF-beta receptor type II deficiency results in defects of yolk sac hematopoiesis and vasculogenesis. *Dev Biol*. Oct 10 1996;179(1):297-302. doi:10.1006/dbio.1996.0259

106. Patzek S, Liu Z, de la O S, et al. Loss of Fgf9 in mice leads to pancreatic hypoplasia and asplenia. *iScience*. Apr 21 2023;26(4):106500. doi:10.1016/j.isci.2023.106500

107. Paudyal A, Damrau C, Patterson VL, et al. The novel mouse mutant, chuzhoi, has disruption of Ptk7 protein and exhibits defects in neural tube, heart and lung development and abnormal planar cell polarity in the ear. *BMC Dev Biol*. Aug 12 2010;10:87. doi:10.1186/1471-213X-10-87

108. Pearson HB, Perez-Mancera PA, Dow LE, et al. SCRIB expression is deregulated in human prostate cancer, and its deficiency in mice promotes prostate neoplasia. *J Clin Invest*. Nov 2011;121(11):4257-67. doi:10.1172/JCI58509

109. Person AD, Beiraghi S, Sieben CM, et al. WNT5A mutations in patients with autosomal dominant Robinow syndrome. *Dev Dyn*. Jan 2010;239(1):327-37. doi:10.1002/dvdy.22156

110. Piedrahita JA, Oetama B, Bennett GD, et al. Mice lacking the folic acid-binding protein Folbp1 are defective in early embryonic development. *Nat Genet*. Oct 1999;23(2):228-32. doi:10.1038/13861

111. Pospichalova V, Tureckova J, Fafilek B, et al. Generation of two modified mouse alleles of the Hic1 tumor suppressor gene. *Genesis*. Mar 2011;49(3):142-51. doi:10.1002/dvg.20719

112. Purandare SM, Ware SM, Kwan KM, et al. A complex syndrome of left-right axis, central nervous system and axial skeleton defects in Zic3 mutant mice. *Development*. May 2002;129(9):2293-302. doi:10.1242/dev.129.9.2293

113. Pütz S, Barthel LS, Frohn M, et al. Caldesmon ablation in mice causes umbilical herniation and alters contractility of fetal urinary bladder smooth muscle. *J Gen Physiol*. Jul 05 2021;153(7)doi:10.1085/jgp.202012776

114. Pyrgaki C, Liu A, Niswander L. Grainyhead-like 2 regulates neural tube closure and adhesion molecule expression during neural fold fusion. *Dev Biol*. May 01 2011;353(1):38-49. doi:10.1016/j.ydbio.2011.02.027

115. Qian C, Wong CWY, Wu Z, et al. Stage specific requirement of platelet-derived growth factor receptor-α in embryonic development. *PLoS One*. 2017;12(9):e0184473. doi:10.1371/journal.pone.0184473

116. Qu S, Niswender KD, Ji Q, et al. Polydactyly and ectopic ZPA formation in Alx-4 mutant mice. *Development*. Oct 1997;124(20):3999-4008. doi:10.1242/dev.124.20.3999

117. Qu S, Tucker SC, Zhao Q, deCrombrugghe B, Wisdom R. Physical and genetic interactions between Alx4 and Cart1. *Development*. Jan 1999;126(2):359-69. doi:10.1242/dev.126.2.359

118. Radek KA, Taylor KR, Gallo RL. FGF-10 and specific structural elements of dermatan sulfate size and sulfation promote maximal keratinocyte migration and cellular proliferation. *Wound Repair Regen*. 2009;17(1):118-26. doi:10.1111/j.1524-475X.2008.00449.x

119. Ramírez-Solis R, Zheng H, Whiting J, Krumlauf R, Bradley A. Hoxb-4 (Hox-2.6) mutant mice show homeotic transformation of a cervical vertebra and defects in the closure of the sternal rudiments. *Cell*. Apr 23 1993;73(2):279-94. doi:10.1016/0092-8674(93)90229-j

120. Rauch F, Prud'homme J, Arabian A, Dedhar S, St-Arnaud R. Heart, brain, and body wall defects in mice lacking calreticulin. *Exp Cell Res*. Apr 10 2000;256(1):105-11. doi:10.1006/excr.2000.4818

121. Rifat Y, Parekh V, Wilanowski T, et al. Regional neural tube closure defined by the Grainy head-like transcription factors. *Dev Biol*. Sep 15 2010;345(2):237-45. doi:10.1016/j.ydbio.2010.07.017

122. Roebroek AJ, Umans L, Pauli IG, et al. Failure of ventral closure and axial rotation in embryos lacking the proprotein convertase Furin. *Development*. Dec 1998;125(24):4863-76. doi:10.1242/dev.125.24.4863

123. Saito K, Kakizaki T, Hayashi R, et al. The physiological roles of vesicular GABA transporter during embryonic development: a study using knockout mice. *Mol Brain*. Dec 30 2010;3:40. doi:10.1186/1756-6606-3-40

124. Sasaki A, Masuda Y, Ohta Y, Ikeda K, Watanabe K. Filamin associates with Smads and regulates transforming growth factor-beta signaling. *J Biol Chem*. May 25 2001;276(21):17871-7. doi:10.1074/jbc.M008422200

125. Schatteman GC, Morrison-Graham K, van Koppen A, Weston JA, Bowen-Pope DF. Regulation and role of PDGF receptor alpha-subunit expression during embryogenesis. *Development*. May 1992;115(1):123-31. doi:10.1242/dev.115.1.123

126. Schorle H, Meier P, Buchert M, Jaenisch R, Mitchell PJ. Transcription factor AP-2 essential for cranial closure and craniofacial development. *Nature*. May 16 1996;381(6579):235-8. doi:10.1038/381235a0

127. Serin N, Dihazi GH, Tayyeb A, et al. Calreticulin Deficiency Disturbs Ribosome Biogenesis and Results in Retardation in Embryonic Kidney Development. *Int J Mol Sci*. May 30 2021;22(11)doi:10.3390/ijms22115858

128. Shen J, Bronson RT, Chen DF, Xia W, Selkoe DJ, Tonegawa S. Skeletal and CNS defects in Presenilin-1-deficient mice. *Cell*. May 16 1997;89(4):629-39. doi:10.1016/s0092-8674(00)80244-5

129. Shi J, Wei L. Rho Kinases in Embryonic Development and Stem Cell Research. *Arch Immunol Ther Exp (Warsz)*. Jan 19 2022;70(1):4. doi:10.1007/s00005-022-00642-z

130. Shih HP, Gross MK, Kioussi C. Expression pattern of the homeodomain transcription factor Pitx2 during muscle development. *Gene Expr Patterns*. Feb 2007;7(4):441-51. doi:10.1016/j.modgep.2006.11.004

131. Shimizu Y, Thumkeo D, Keel J, et al. ROCK-I regulates closure of the eyelids and ventral body wall by inducing assembly of actomyosin bundles. *J Cell Biol*. Mar 14 2005;168(6):941-53. doi:10.1083/jcb.200411179

132. Simón-Carrasco L, Graña O, Salmón M, et al. Inactivation of Capicua in adult mice causes T-cell lymphoblastic lymphoma. *Genes Dev*. Jul 15 2017;31(14):1456-1468. doi:10.1101/gad.300244.117

133. Singh AP, Castranio T, Scott G, et al. Influences of reduced expression of maternal bone morphogenetic protein 2 on mouse embryonic development. *Sex Dev*. 2008;2(3):134-41. doi:10.1159/000143431

134. Smits P, Bolton AD, Funari V, et al. Lethal skeletal dysplasia in mice and humans lacking the golgin GMAP-210. *N Engl J Med*. Jan 21 2010;362(3):206-16. doi:10.1056/NEJMoa0900158

135. Snowball J, Ambalavanan M, Cornett B, Lang R, Whitsett J, Sinner D. Mesenchymal Wnt signaling promotes formation of sternum and thoracic body wall. *Dev Biol*. May 15 2015;401(2):264-75. doi:10.1016/j.ydbio.2015.02.014

136. Sock E, Rettig SD, Enderich J, Bösl MR, Tamm ER, Wegner M. Gene targeting reveals a widespread role for the high-mobility-group transcription factor Sox11 in tissue remodeling. *Mol Cell Biol*. Aug 2004;24(15):6635-44. doi:10.1128/MCB.24.15.6635-6644.2004

137. Soriano P. The PDGF alpha receptor is required for neural crest cell development and for normal patterning of the somites. *Development*. Jul 1997;124(14):2691-700. doi:10.1242/dev.124.14.2691

138. Sun J, Liu YH, Chen H, et al. Deficient Alk3-mediated BMP signaling causes prenatal omphalocele-like defect. *Biochem Biophys Res Commun*. Aug 17 2007;360(1):238-43. doi:10.1016/j.bbrc.2007.06.049

139. Suzuki N, Labosky PA, Furuta Y, et al. Failure of ventral body wall closure in mouse embryos lacking a procollagen C-proteinase encoded by Bmp1, a mammalian gene related to Drosophila tolloid. *Development*. Nov 1996;122(11):3587-95. doi:10.1242/dev.122.11.3587

140. Szumska D, Pieles G, Essalmani R, et al. VACTERL/caudal regression/Currarino syndrome-like malformations in mice with mutation in the proprotein convertase Pcsk5. *Genes Dev*. Jun 01 2008;22(11):1465-77. doi:10.1101/gad.479408

141. Takahashi M, Tamura M, Sato S, Kawakami K. Mice doubly deficient in. *Dis Model Mech*. Oct 25 2018;11(10)doi:10.1242/dmm.034611

142. Thumkeo D, Shimizu Y, Sakamoto S, Yamada S, Narumiya S. ROCK-I and ROCK-II cooperatively regulate closure of eyelid and ventral body wall in mouse embryo. *Genes Cells*. Aug 2005;10(8):825-34. doi:10.1111/j.1365-2443.2005.00882.x

143. Tsang SM, Oliemuller E, Howard BA. Regulatory roles for SOX11 in development, stem cells and cancer. *Semin Cancer Biol*. Dec 2020;67(Pt 1):3-11. doi:10.1016/j.semcancer.2020.06.015

144. Tullio AN, Accili D, Ferrans VJ, et al. Nonmuscle myosin II-B is required for normal development of the mouse heart. *Proc Natl Acad Sci U S A*. Nov 11 1997;94(23):12407-12. doi:10.1073/pnas.94.23.12407

145. Van Otterloo E, Milanda I, Pike H, et al. AP-2α and AP-2β cooperatively function in the craniofacial surface ectoderm to regulate chromatin and gene expression dynamics during facial development. *Elife*. Mar 25 2022;11doi:10.7554/eLife.70511

146. Wang J, Nakamura F. Identification of Filamin A Mechanobinding Partner II: Fimbacin Is a Novel Actin Cross-Linking and Filamin A Binding Protein. *Biochemistry*. Nov 26 2019;58(47):4737-4743. doi:10.1021/acs.biochem.9b00101

147. Wang X, Bolotin D, Chu DH, Polak L, Williams T, Fuchs E. AP-2alpha: a regulator of EGF receptor signaling and proliferation in skin epidermis. *J Cell Biol*. Jan 30 2006;172(3):409-21. doi:10.1083/jcb.200510002

148. Wojcik SM, Katsurabayashi S, Guillemin I, et al. A shared vesicular carrier allows synaptic corelease of GABA and glycine. *Neuron*. May 18 2006;50(4):575-87. doi:10.1016/j.neuron.2006.04.016

149. Yadav SP, Sharma NK, Liu C, Dong L, Li T, Swaroop A. Centrosomal protein CP110 controls maturation of the mother centriole during cilia biogenesis. *Development*. May 01 2016;143(9):1491-501. doi:10.1242/dev.130120

150. Yamada R, Mizutani-Koseki Y, Koseki H, Takahashi N. Requirement for Mab21l2 during development of murine retina and ventral body wall. *Dev Biol*. Oct 15 2004;274(2):295-307. doi:10.1016/j.ydbio.2004.07.016

151. Yamaguchi TP, Bradley A, McMahon AP, Jones S. A Wnt5a pathway underlies outgrowth of multiple structures in the vertebrate embryo. *Development*. Mar 1999;126(6):1211-23. doi:10.1242/dev.126.6.1211

152. Yan Y, Frisén J, Lee MH, Massagué J, Barbacid M. Ablation of the CDK inhibitor p57Kip2 results in increased apoptosis and delayed differentiation during mouse development. *Genes Dev*. Apr 15 1997;11(8):973-83. doi:10.1101/gad.11.8.973

153. Zhang J, Hagopian-Donaldson S, Serbedzija G, et al. Neural tube, skeletal and body wall defects in mice lacking transcription factor AP-2. *Nature*. May 16 1996;381(6579):238-41. doi:10.1038/381238a0

154. Zhang L, Li H, Yu J, et al. Ectodermal Wnt signaling regulates abdominal myogenesis during ventral body wall development. *Dev Biol*. Mar 01 2014;387(1):64-72. doi:10.1016/j.ydbio.2013.12.027

155. Zhang P, Liégeois NJ, Wong C, et al. Altered cell differentiation and proliferation in mice lacking p57KIP2 indicates a role in Beckwith-Wiedemann syndrome. *Nature*. May 08 1997;387(6629):151-8. doi:10.1038/387151a0

156. Zhao GQ, Eberspaecher H, Seldin MF, de Crombrugghe B. The gene for the homeodomain-containing protein Cart-1 is expressed in cells that have a chondrogenic potential during embryonic development. *Mech Dev*. Dec 1994;48(3):245-54. doi:10.1016/0925-4773(94)90063-9

157. Zhu X, Huang S, Zhang L, et al. Constitutive activation of ectodermal β-catenin induces ectopic outgrowths at various positions in mouse embryo and affects abdominal ventral body wall closure. *PLoS One*. 2014;9(3):e92092. doi:10.1371/journal.pone.0092092
